# Selective blockade of microRNA-31-5p−calcitonin receptor interaction reverses established atrial fibrosis and atrial arrhythmia burden

**DOI:** 10.1038/s44161-026-00897-1

**Published:** 2026-10-30

**Authors:** Jasha Trompf, Kathryn Cox, Mohit Hulsurkar, Lucia M. Moreira, Chi Him Kendrick Yiu, Aaron M. Johnston, Satadru K. Lahiri, Shuai Zhao, Paul Robinson, Roddy Hiram, Mozhdeh Mehdizadeh, Lorena Perez Carrillo, Rana Sayeed, George Krasopoulos, Vivek Srivastava, Nicholas Walcot, Shakil Farid, Antonios Kourliouros, Priya Sastry, David A. Menassa, Benjamin Davies, Rita A. Schack, Robia G. Pautler, Keith M. Channon, Craig Lygate, Stanley Nattel, Xander H. T. Wehrens, Svetlana Reilly

**Affiliations:** 1 Division of Cardiovascular Medicine, Radcliffe Department of Medicine, British Heart Foundation Centre of Research Excellence, University of Oxford, John Radcliffe Hospital, Oxford, UK; 2 Cardiovascular Research Institute, Baylor College of Medicine, Houston, TX USA; 3 Department of Integrative Physiology, Baylor College of Medicine, Houston, TX USA; 4 Department of Pharmacology and Physiology, Faculty of Medicine and Research Centre, Montreal Heart Institute and University of Montreal, Montreal, Quebec Canada; 5 Cardiothoracic Surgery, Oxford Heart Centre, John Radcliffe Hospital, Oxford, UK; 6 Clinical Neurology, Nuffield Department of Clinical Neurosciences, University of Oxford, John Radcliffe Hospital, Oxford, UK; 7 School of Cardiovascular & Metabolic Health, University of Glasgow, Glasgow, UK; 8 Department of Pharmacology and Therapeutics, McGill University, Montreal, Quebec Canada; 9 Institute of Pharmacology, West German Heart and Vascular Center, University Duisburg-Essen, Essen, Germany; 10 IHU LIRYC and Fondation Bordeaux Université, Bordeaux, France

**Keywords:** Cell signalling, Non-coding RNAs

## Abstract

Atrial fibrillation (AF) is the most common sustained cardiac arrhythmia and is promoted by atrial fibrosis, a form of structural remodelling for which effective therapies are lacking. MicroRNA-31-5p (miR-31-5p), an aging-associated, atrial-enriched microRNA, has been linked to AF, but its role in atrial fibrosis is unknown. Here we show that atrial fibroblasts from patients with persistent AF exhibit increased miR-31-5p expression and reduced expression of the calcitonin receptor (CTR, encoded by *CALCR*). MiR-31-5p directly represses CTR and promotes pro-fibrotic fibroblast responses, whereas its inhibition restores CTR expression. Fibroblast-specific overexpression of miR-31 in mice reduces atrial CTR expression, increases atrial fibrosis and enhances arrhythmia susceptibility. Selective disruption of miR-31-5p−*CALCR* binding using a locked nucleic acid target site blocker restores atrial CTR expression, prevents fibrosis progression and reduces arrhythmia burden. In mice with established atrial remodeling, this strategy promotes fibrosis regression, identifying the miR-31-5p−CTR axis as a potential therapeutic target in AF.

## Abstract (web summary)

Trompf, Cox, Hulsurkar et al. show that the selective disruption of the miR-31-5p−CTR interaction in atrial fibrillation reverses established atrial fibrosis and reduces atrial fibrillation susceptibility, highlighting a promising RNA-based therapeutic strategy.

## Main

AF, the most common cardiac arrhythmia in humans, is a growing health concern with an estimated incidence predicted to double by 2060, largely due to population aging^1,2^. Aging is the strongest risk factor for AF and contributes to the atrial remodeling that promotes arrhythmia development and progression^2^. AF is a major contributor to global mortality and morbidity, mainly due to stroke (AF alone is responsible for approximately 30−40% of all strokes) and heart failure^1,2^. Treatment of AF remains a major clinical challenge because of the underlying myocardial electrical and structural remodeling^3,4^. Electrical remodeling, caused by changes in ion channel expression/function, is largely reversible with maintenance of a normal sinus rhythm (SR) with the currently available anti-arrhythmic drugs, cardioversion and catheter ablation. By contrast, structural remodeling, hallmarked by atrial fibrosis, has not been successfully targeted by currently available therapies and is of particular concern in the clinic, as atrial fibrosis hampers AF treatment and worsens prognosis^5,6^. Thus, there is a quest to identify players in atrial fibrosis and cardiac remodeling to allow the development of more effective therapeutics for patients with this serious condition^7^.

Characterized by increased extracellular matrix (ECM) deposition without a significant loss of atrial cardiomyocytes (ACMs), atrial fibrosis increases as AF progresses^4^ and is a key feature of cardiac remodeling in AF^8^. The degree of atrial fibrosis negatively correlates with the success of one of the most successful therapeutic options: catheter ablation^9,10^. Despite extensive prior work, which uncovered key signaling pathways such as those linked to transforming growth factor β1 (TGFβ1), galectin-3 and the renin−angiotensin−aldosterone system in atrial fibrosis, this insight has failed to yield clinically effective therapeutics to reverse atrial fibrosis. Therefore, there remains a major need to identify new players in atrial remodeling that can be targeted to generate more effective therapeutics for patients with AF^7,9,10^.

Atrial fibrosis is facilitated by complex interactions between cellular and neurohormonal mediators^11,12^, including a recently discovered cardioendocrine calcitonin (CT) system^13^. CT is a well-established thyroid hormone that has traditionally been recognized for its role in regulating calcium homeostasis and inhibiting bone resorption^14^. In previous work, we demonstrated that ACM-derived CT acts in a paracrine manner via binding to calcitonin receptors (CTRs) on neighboring atrial fibroblasts; this limits fibroblast activation and fibrosis, highlighting its protective role in atrial structural remodeling^13^. Although the existence of the CT−CTR axis and its dysregulation in persistent AF has been established, the upstream mechanisms responsible for reduced CTR expression remained unknown. Therefore, in the present study, we investigated whether miR-31-5p regulates CTR abundance in human atrial fibroblasts and whether selective blockade of the miR-31-5p−CTR interaction can reverse established atrial fibrosis and arrhythmia in vivo.

This axis is dysfunctional in the atria of patients with advanced atrial structural and electrical remodeling, such as in persistent atrial fibrillation (persAF)^13^. Cardiac CT produced by human ACMs controls proliferation, migration and ECM production of atrial cardiofibroblasts (ACFs)^13^. Disrupted signaling along this paracrine axis promotes collagen deposition, atrial fibrosis and AF in vivo in mice^13^. Patients with persAF (defined as AF episodes lasting longer than 7 days) have six-fold lower atrial CT production, reduced CTR expression and increased receptor internalization, facilitating enhanced collagen deposition by ACFs^13^. Thus, restoring cardiac CT−CTR signaling represents an attractive strategy to overcome major challenges in controlling cardiac remodeling in AF. Although deficient CT can be replenished with exogenous CT analogues (for example, miacalcin, salmon CT) already in clinical use (for example, for Paget’s disease, hypercalcemia, osteoporosis^14^), strategies to restore atrial CTR expression in persAF, a prerequisite for CT-mediated atrial anti-fibrotic and anti-arrhythmic effects, do not exist.

Given the reduced CTR protein in the absence of changes in CTR mRNA in ACFs from patients with persAF^13^, it is plausible that microRNAs (miRs) may be involved in repressing CTR signaling. MiRs are endogenous small (19−27 nucleotides) noncoding RNAs which, via binding to a (partially) complementary sequence of 3′ untranslated mRNA region (3′ UTR or, less commonly, 5′ UTR/coding sequence regions), mediate gene silencing by accelerating mRNA degradation or repressing translation^15^. Previous work reported numerous miRs implicated in AF^16^, including atrial-enriched miR-31*-*5p^17,18^, which, alongside three other miRs, is strongly predicted to bind the 3′ UTR of the human CTR gene (*CALCR*). MiR-31*-*5p is implicated in ACM electrical remodeling in patients with persAF^18^, in aging (the strongest risk factor for AF^19^) and in age-related/senescence-related processes such as cellular senescence, cancer and osteoporosis^20–22^ (a condition associated with suppressed CT signaling). Our pilot work demonstrated that ACFs from patients with AF, which are characterized by suppressed CTR protein levels, produce twice the amount of miR-31*-*5p. Therefore, the present study aimed to explore (1) the role of miR-31*-*5p as an upstream regulator of CTR signaling in remodeled atrial myocardium from patients with persAF and (2) the therapeutic potential of targeting the miR-31*-*5p−CTR mRNA interaction to prevent and/or reverse atrial fibrosis and arrhythmia. Although previous work^13^ established the existence of the CT−CTR axis and its dysregulation in persAF, the present study identifies the upstream regulatory mechanism—miR-31-5p—that controls CTR abundance in human atrial fibroblasts and demonstrates that selective blockade of the miR-31-5p−CTR interaction can reverse established atrial fibrosis and arrhythmia in vivo.

## Results

### *MiRNA-*31*-*5*p* regulates CTR abundance in human ACFs

**Fig. 1:**
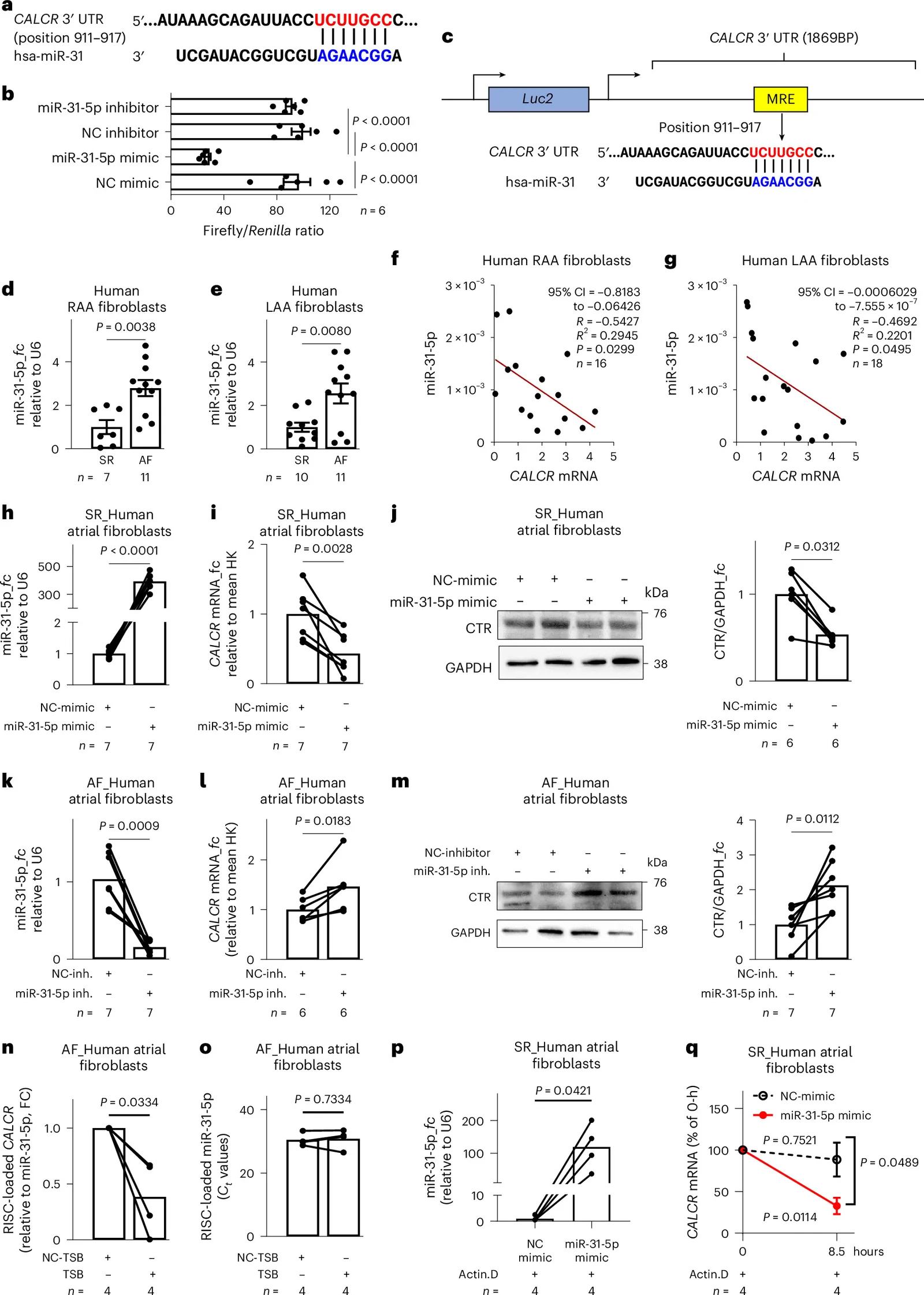
Atrial-specific miR-31*-*5p controls CTR expression in human atrial fibroblasts. **a**, In silico predicted miRNA recognition element (MRE) between hsa-miR*-*31*-*5p and 3′ UTR of CTR mRNA (*CALCR)*. **b**, Luciferase reporter assay in HEK293 cells transfected with miR-31 mimic, miR-31*-*5p inhibitor or respective negative controls (NC mimic, NC inhibitor). **c**, Diagram of the luciferase reporter construct used in **b**. **d**,**e**, Level of miR-31*-*5p expression in RAA (**d**) and LAA (**e**) hACFs collected from patients with persAF versus control in SR. **f**,**g**, Correlation between miR-31*-*5p and *CALCR* in human RAA (**f**) and LAA (**g**) ACFs. **h**−**m**, Efficient transfection of human atrial fibroblasts from patients in SR with miR-31-5p mimic (**h**) or patients with persAF with miR-31-5p inhibitor (**k**). Effect of miR-31 overexpression on CTR (*CALCR*) gene (**i**) and CTR protein (**j**) expression in SR fibroblasts and effect of miR-31-5p inhibition on *CALCR* gene (**l**) and protein (**m**) expression in AF fibroblasts. **n**,**o**, Prevention of miR-31 binding to *CALCR* with MRE-specific miR-31/CTR-TSB (versus NC-TSB) reduces loading of *CALCR* (**n**) into RISC, without altering miR-31*-*5p RISC loading (**o**). **p**,**q**, Effect of miR-31*-*5p overexpression (**p**) or NC mimic on *CALCR* (normalized to a 0-hour (0-h) control) in human SR ACFs (**q**) pre-treated with actinomycin D (Actin.D); *P* value shows interaction for miR-31 mimic treatment. Data are expressed as mean ± s.e.m., except median with interquartile range in **j**. *P* values were calculated by one-way ANOVA with Holm−Sidak test (**b**), unpaired *t*-test (**d**,**e**), Pearson’s correlation test (**f**,**g**), paired *t*-test (**h** **i**,**k**−**m**,**o**,**p**), one-sample *t*-test (**n**), Wilcoxon test (**j**) or two-way repeated-measures ANOVA (**q**). CI, confidence interval; FC, fold change of control group; HK, housekeeping gene; *n*, biologically independent donors. *n* values are shown in the graphs. All statistical tests were two-sided unless otherwise stated. Source data

**Extended Data Fig. 1:**
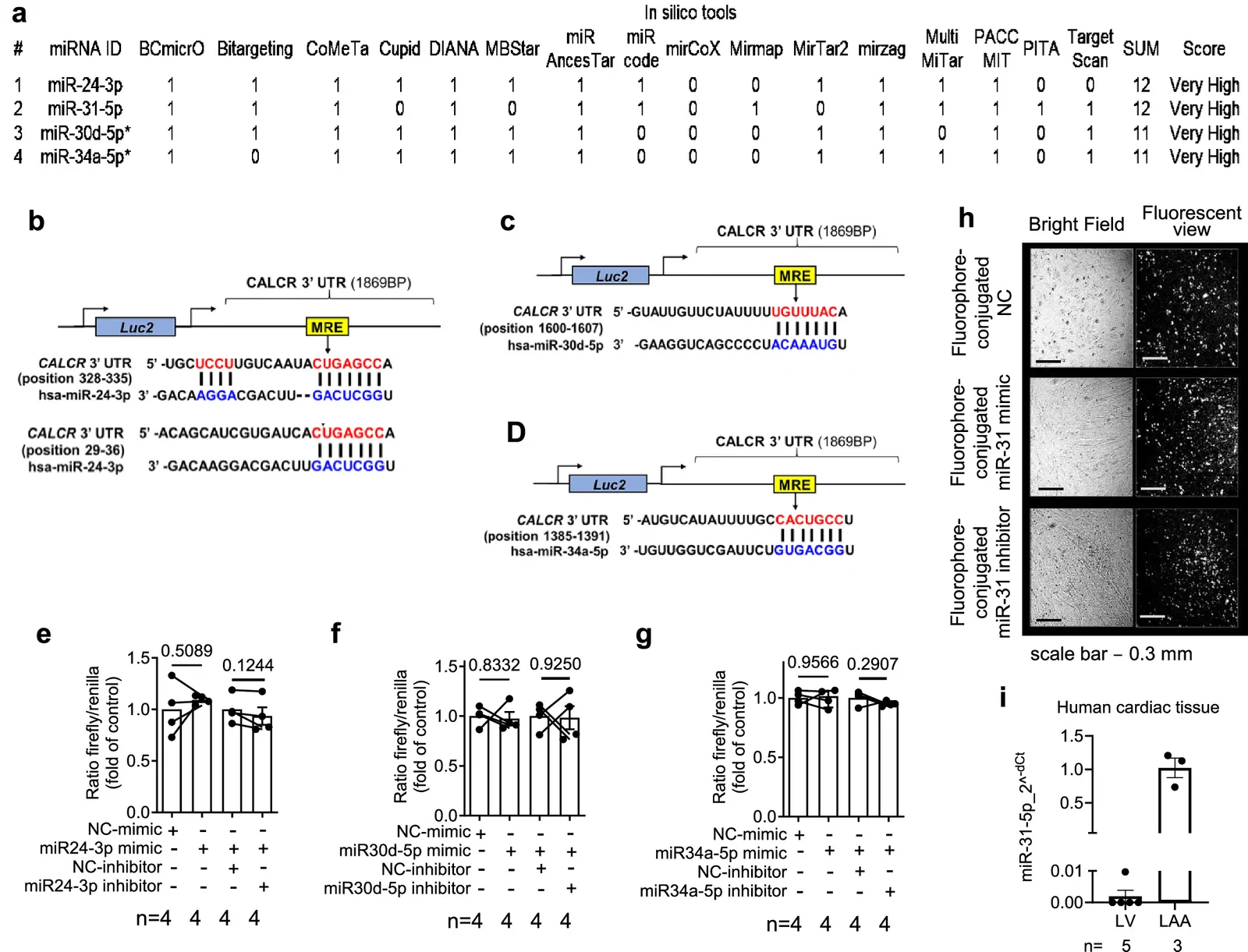
Validation of miR-31-5p as a regulator of human atrial fibroblasts (hACFs). (**a**) List of the top miRs predicted (by *mirDIP* tool) to bind human CTR mRNA (*CALCR*). (**b-d**) Schematic diagrams of the constructs used in the reporter assay for *hsa-miR-*24*-*3*p, -* 30*d-*5*p*, and -34*a-*5*p*; binding sites are shown in blue-red font; MRE, miRNA response element. (**e-g**) Reporter assay in HEK293 cells co-transfected (24 h) with mimic for *hsa-miR-*24*-*3*p, -* 30*d-*5*p*, and *-*34*a-*5*p* and a construct contacting CTR-mRNA 3′UTR). (**h**) Representative bright field and fluorescence images of hACFs 24- hour post-transfection with fluorophore-conjugated oligonucleotides. (**i**) Expression levels (qPCR) of miR- 31-5p in human ventricular and atrial myocardium. Data are expressed as mean ± SEM. P-values are calculated by paired t test; FC, fold change of control group; n, individual donors. Source data

Using published reports (Supplementary Table 1) of the miRs altered in atrial tissue or cells in AF, we identified, using in silico mirDIP analysis at https://ophid.utoronto.ca/mirDIP/, four miRs (hsa-miR-24*-*3p, *-*30d-5p, *-*31*-*5p and -34a-5p), which are strongly predicted to bind CTR mRNA (*CALCR*) 3′ UTR (Extended Data Fig. 1a). A reporter assay showed no binding between *CALCR* 3′ UTR and hsa-miR-24*-*3p, *-*30d-5p or *-*34a-5p in HEK293 cells co-transfected with pmiRGLO luciferase/*Renilla*-expressing plasmid containing full-length *CALCR* 3′ UTR and the respective miR mimic/inhibitor (versus mimic/inhibitor non-targeting negative controls (NCs)) (Extended Data Fig. 1b–h). By contrast, the reporter assay showed approximately 60% reduction of the luciferase/*Renilla* ratio (*P* < 0.0001) in cells transfected with miR-31*-*5p mimic (versus NC mimic) that was fully reversed by miR-31*-*5p inhibition, indicating that miR-31*-*5p binds the *CALCR* 3′ UTR (Fig. 1a–c). Although upregulation of miR-31*-*5p has been reported in human ACMs and noted to be relevant to electrical remodeling in AF^17,18^, its role in ACFs and its potential contributions to AF fibrogenesis is unknown. We found that, in patients (Supplementary Table 2) with persAF, compared to SR controls, ACFs from both right (RAA; *P* = 0.0038) and left (LAA; *P* = 0.008) atrial appendages had 2.7-fold and 2.5-fold increased miR-31*-*5p expression, respectively (Fig. 1d,e); notably, miR-31-5p expression was barely detectable in the left ventricular myocardium (Extended Data Fig. 1i). Moreover, miR-31*-*5p levels negatively correlated with *CALCR* expression in these ACFs (RAA: *P* = 0.0299; LAA: *P* = 0.0495; Fig. 1f,g). To test the effect of miR-31-5p on CTR gene and protein expression in native ACFs, ACFs isolated from patients with SR and from patients with persAF were transfected with miR-31*-*5p mimic (or NC mimic) and miR-31*-*5p inhibitor (or NC inhibitor), respectively. Overexpression of miR-31*-*5p (Fig. 1h) halved CTR mRNA (*P* = 0.0028) and protein (*P* = 0.0312) (Fig. 1i,j), whereas miR-31*-*5p inhibition (Fig. 1k) increased CTR mRNA by approximately 30% (*P* = 0.0183) and nearly doubled CTR protein (*P* = 0.0112) versus NC-treated ACFs (Fig. 1l,m). These results indicate that miR-31*-*5p inhibition is sufficient to restore the decreased ACF CTR expression previously observed in persAF^13^.

To confirm the endogenous interaction between miR-31*-*5p and *CALCR* (that is, occupancy of miR-31-5p on *CALCR* 3′ UTR), we assessed, with previously validated RNA-binding protein immunoprecipitation (RIP) assay and quantitative polymerase chain reaction (qPCR)^17^, the loading of both targets into the RNA-induced silencing complex (RISC) in AF ACFs. Although both *CALCR* and miR-31*-*5p were present in Ago2 RISC pulldowns (Fig. 1n,o), a selective blockade of the miR-31*-*5p−*CALCR* interaction with miRCURY LNA miRNA Power Target Site Blocker (miR-31/CTR-TSB) decreased *CALCR*, but not miR-31*-*5p, RISC content by approximately 60% (*P* = 0.0334) (Fig. 1n,o), further confirming an endogenous miR-31*-*5p−*CALCR* interaction in human AF ACFs. To elucidate the mechanism (enhanced mRNA degradation or translational silencing) by which miR-31*-*5p regulates *CALCR*, we compared the effects of miR-31*-*5p mimic (or NC mimic) (Fig. 1p) on *CALCR* levels in human atrial cardiofibroblasts (hACFs) pre-treated with 5 µg ml^−1^ actinomycin D, a potent, non-selective transcription inhibitor^18^; *CALCR* diminished faster in the presence of miR-31*-*5p mimic (versus NC mimic) (*P* = 0.0114), suggesting that enhanced mRNA degradation contributes to the regulation of CTR expression by miR-31*-*5p in hACFs (Fig. 1q).

### *MiRNA-*31*-*5*p* regulates CTR expression in atrial fibroblasts in mice

**Fig. 2:**
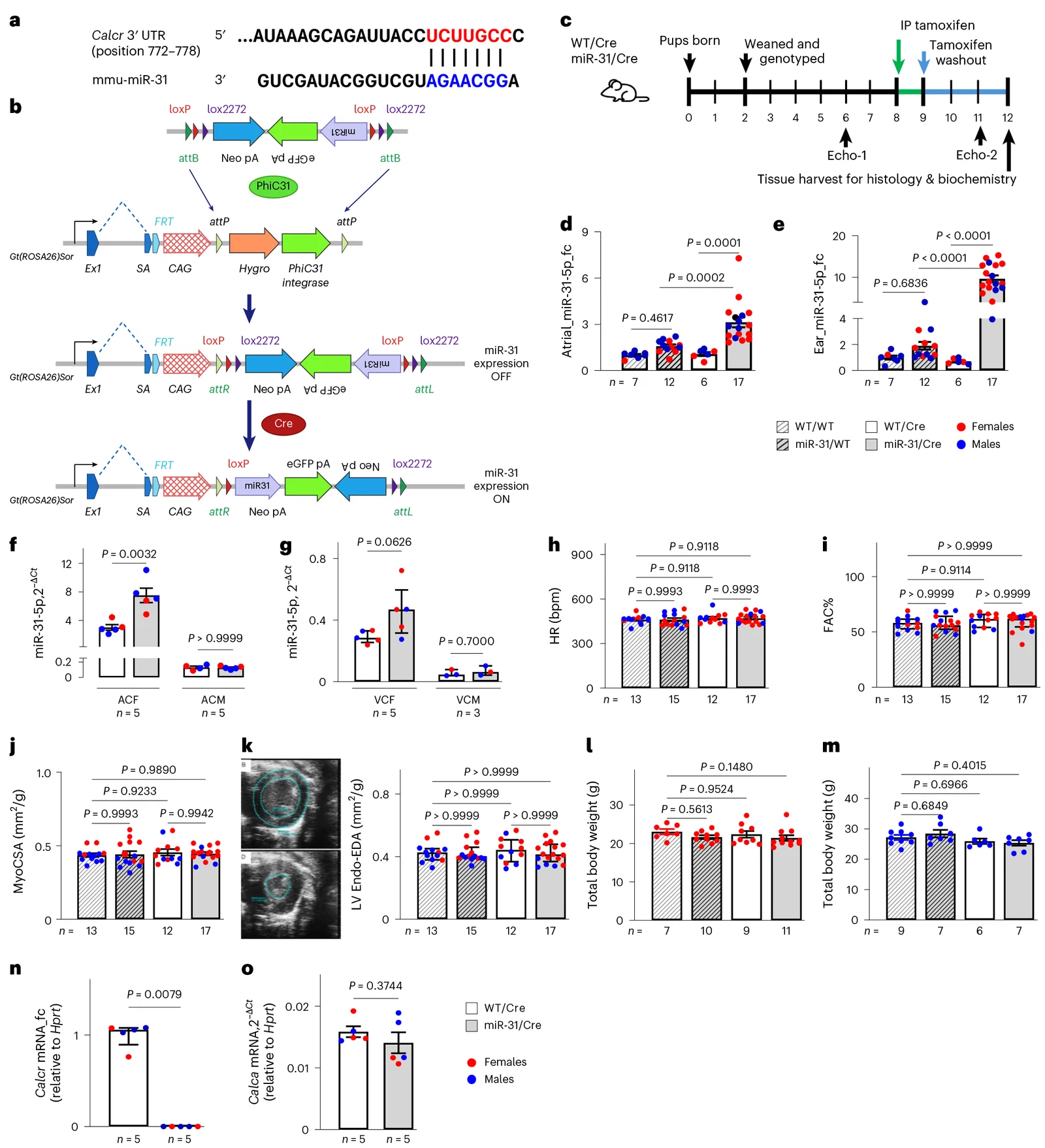
Induced fibroblast-targeted miR-31 overexpression inhibits CTR expression in mice. **a**, In silico predicted miRNA recognition element (MRE) between mmu-miR*-*31*-*5p and 3′ UTR of CTR mRNA (*Calcr*). **b**, Schematic representation of the constructs used to generate fibroblast-specific miR-31/Cre mice. **c**, Experimental timeline for miR-31/Cre mice studies. **d**,**e**, Validation of generation of mice overexpressing miR-31: atrial tissue (**d**) and ear (**e**) expression of mmu-miR-31*-*5p in all four genotypes (WT/WT, WT/Cre, miR-31/WT and miR-31/Cre) used to generate the final miR-31/Cre and WT/Cre lines; males are shown in blue and females in red. **f**,**g**, Upregulation of mmu-miR-31*-*5p in atrial (**f**) and left ventricular (**g**) cardiofibroblasts (ACFs, VCFs) and cardiomyocytes (ACMs, VCMs) in WT/Cre and miR-31/Cre male (in blue) and female (in red) mice. **h**, Heart rate in WT/WT, WT/Cre, miR-31/WT and miR-31/Cre genotypes. **i**−**k**, Echocardiography showing unaltered FAC, MyoCSA and LV-Endo-EDA among all four mouse genotypes. Short-axis images in **k** are examples of end-diastolic view of the end-diastolic endocardial and epicardial areas (top panel) and short-axis echocardiograms of the endocardial end-systolic area (bottom panel); data in **j** and **k** are normalized to total body weight; tibia or femur length was not measured in this study. **l**,**m**, Total body weight of females in red (**l**) and males in blue (**m**) in respective genotypes. **n**,**o**, Gene expression of CTR (*Calcr*) and CT (*Calca*) in atrial fibroblasts and cardiomyocytes (qPCR), respectively, in miR-31/Cre and WT/Cre mice. Data are expressed as mean ± s.e.m. or median with interquartile range (**i**,**k**,**l**,**o**). *P* values were calculated by one-way ANOVA with Holm−Sidak test (**d**,**e**,**h**,**j**) or Dunnett’s post hoc test (**m**), Kruskal−Wallis with Dunn’s post hoc test (**i**,**k**,**l**), unpaired *t*-test (**f**, ACFs; **g**, VCFs; **o**) and Mann−Whitney test (**f**, ACMs; **g**, VCMs; **o**). bpm, beats per minute; FC, fold change of control group; HR, heart rate; IP, intraperitoneal; *n*, biologically independent mice. *n* values are shown in the graphs. All statistical tests were two-sided unless otherwise stated. miR-31/Cre and control mice were 12 weeks old. Source data

**Extended Data Fig. 2:**
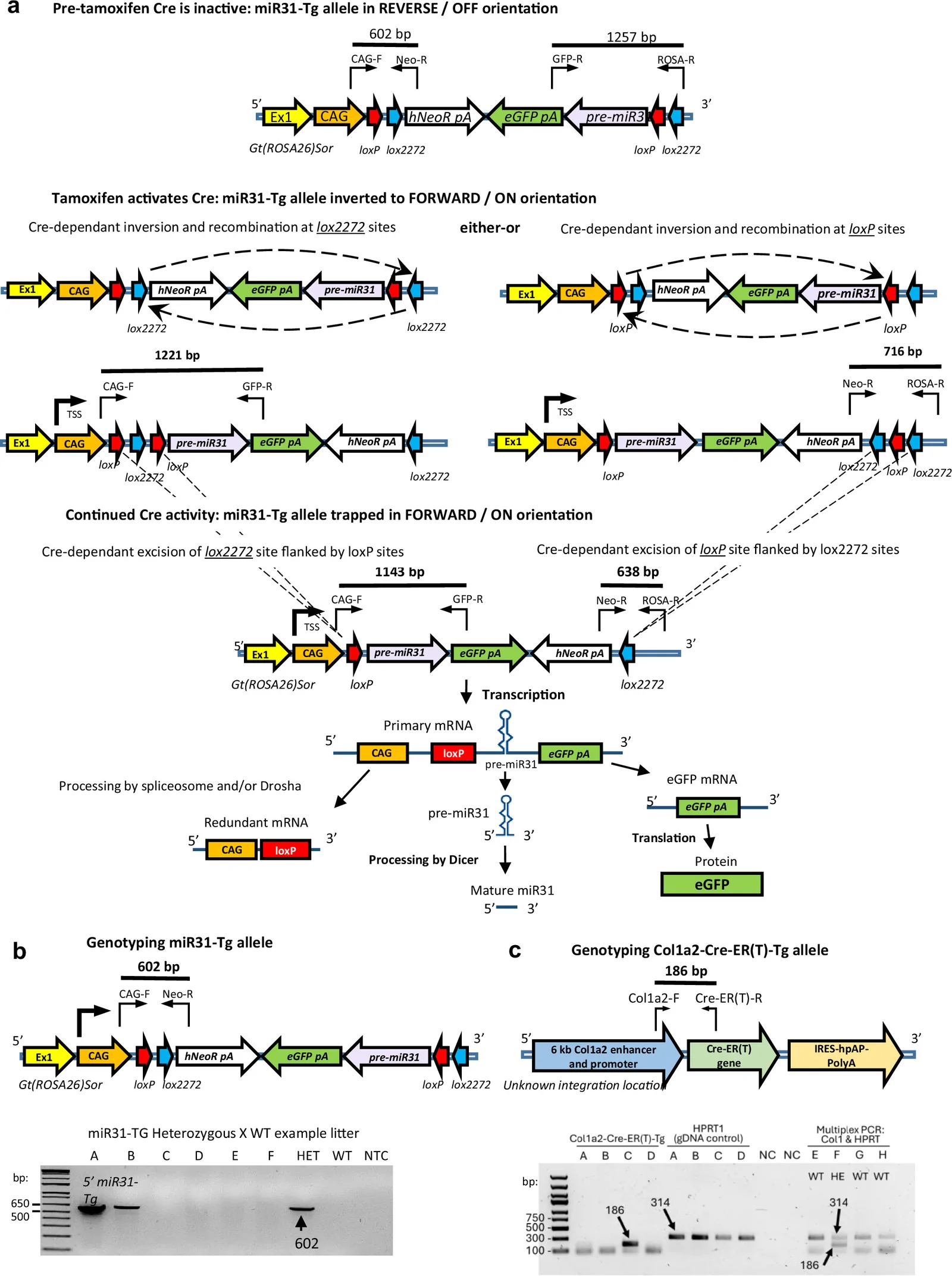
Principle of Cre-recombinase dependent inversion switch at the targeted Gt(ROSA26)Sor locus and breeding strategy to maintain separate miR-31-Tg and Col1A2-Cre-ER(T)-Tg colonies. (**a**) In the nascent state, the transgenic construct containing the pre miR-31 sequence sits inverted with respect to the CAG promoter and is flanked by two pairs of opposing loxP variants. Following tamoxifen treatment and Cre activation, recombination occurs between opposing pairs of variant loxP sites of the same type (loxP or lox2272) and causes the intervening sequence to be inverted and linked to the CAG promoter allowing transcription. Inversion occurs in either of two possible ways and following inversion, Cre excises the short sequence separating parallel loxP sites thereby trapping the entire construct in the forward orientation and inhibiting repeated flipping. The primary mRNA transcript contains CAG and loxP (now redundant), pre miR-31 and eGFP sequences. Processing by the spliceosome and/or Drosha liberates eGFP mRNA and pre miR-31, processed by Dicer, to generate mature miR-31. Genotyping amplicon lengths are depicted and used to track the progress of the miR-31-Tg allele from its nascent to inverted and trapped states. (**b**) Schematic shows the miR-31-Tg allele, primer binding sites and genotyping amplicon length (602 bp). miR-31-Tg colonies were maintained by crossing with WT mice, an example litter is shown with mouse pups labelled A-F. Heterozygous (HET), wild type (WT) and no-template (NTC) controls were used. (**c**) Col1A2-Cre-ER(T) colonies were maintained by breeding hemizygous (HEM) with WT mice and the schematic shows the randomly integrated Col1a2-Cre-ER(T) transgene with primer binding sites and amplicon length (186 bp). Genotyping PCR was performed on an example litter of pups A-L and done as a multiplex with additional primers recognising the WT Hprt1 gene (314 bp) to validate presence of genomic DNA. Source data

**Extended Data Fig. 3:**
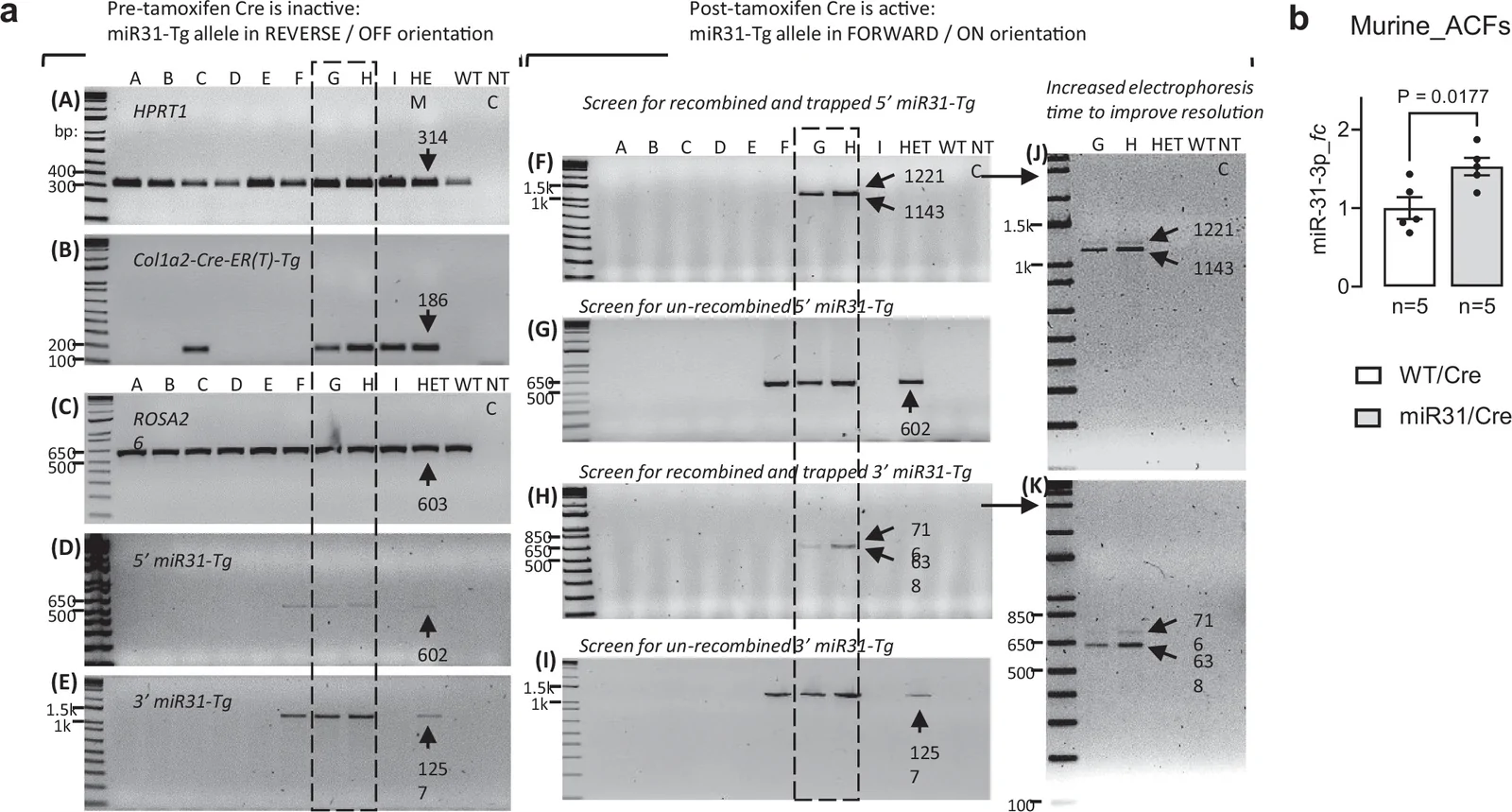
PCR validation of *miR-*31*-Tg* allele inversion and trapping following tamoxifen treatment. (**a**) Heterozygous *miR-*31*-Tg* and hemizygous C*ol*1*A*2*-Cre-ER(T)* mice were crossed and ear gDNA samples were assessed before (left gels) and after (middle-right gels) tamoxifen treatment for littermates (A–I). (A) validates presence of genomic DNA with endogenous HPRT1 gene amplicon (314 bp), (**b**) C*ol*1*A*2*-Cre-ER(T)* gene amplicon (186 bp), (**c**) WT ROSA26 allele (603 bp), (**d**) 5′ end of the nascent *miR-*31*-Tg* (602 bp), (**e**) 3′ end of *miR-*31*-Tg* (1257 bp). Littermates G and H (left dotted box) showed presence of both transgenes. (**f**) Following tamoxifen treatment, the 5′ end of the *miR-*31*-Tg* allele was screened for inversion (1221 bp) and trapping (1143 bp). (**g**) Shows remaining un-recombined 5′ miR-31-Tg (602 bp). (**h** and **i**) shows 3′ miR-31-Tg recombination (716 bp), trapped (638 bp) and remaining un-recombined (1257 bp). Only littermates G and H carry both transgenes and demonstrate the tamoxifen-inducible inversion switch (right dotted box). (**j** and **k**) show samples littermates G and H re-run and electrophoresed for 30 minutes longer to better resolve and visualise band doublets at both the 5′ and 3′ ends of the *miR-*31*-Tg*. Heterozygous (HET), hemizygous (HEM), wild type (WT) and no-template (NTC) controls were used. (b) miR-31-3p expression in atrial fibroblasts (ACFs) in miR-31/Cre and WT/Cre control mice. Data are expressed as mean ± SEM. P-value is calculated by unpaired t test; FC, fold change of control group; n, independent mice. Source data

**Extended Data Fig. 4:**
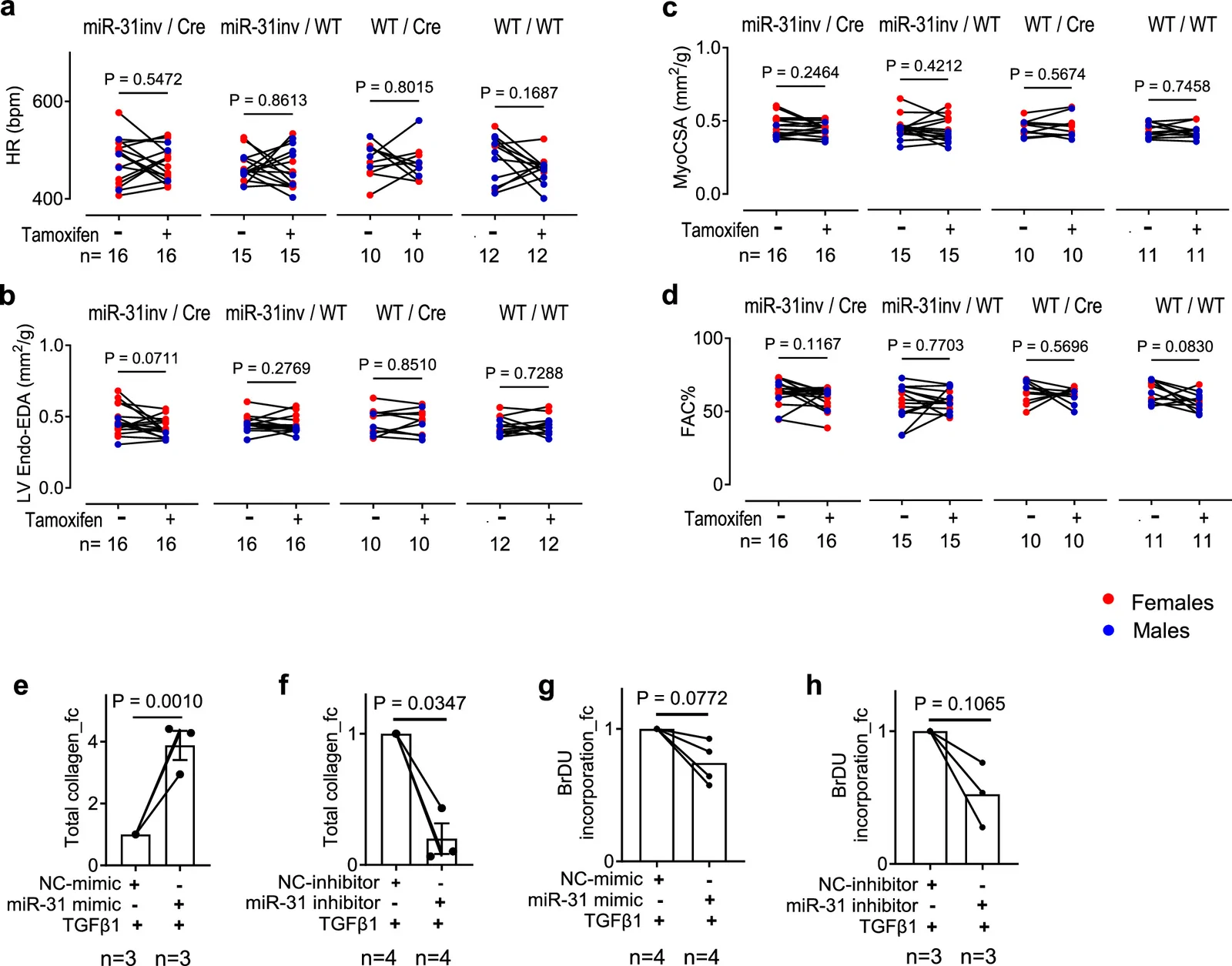
Effect of tamoxifen administration on cardiac function in mice and of miR-31 modulation on TGFβ1-stimulated fibroblast responses. (**a**) Heart rate (HR) is unchanged in mice of all four genotype variations following intraperitoneal tamoxifen administration. (**b-d**) Select echocardiographic parameters are not altered by tamoxifen administration regardless of genotype. (**e-h**) Effect of miR-31 mimic and miR-31 inhibitor or respective NC controls on 72-hour TGFβ1-stimulated total collagen production, and 24-hour cell proliferation of hACFs. Data are expressed as mean ± SEM, except as median with IQR in (b - miR-31inv/Cre; c - for miR-31inv/WT; d – for miR-31inv/Cre and WT/WT). P-values calculated by paired t test (a; b, except ‘miR-31inv/Cre’; c, except ‘miR-31inv/WT’; d, except ‘miR-31inv/Cre’ and ‘WT/WT’; e-h), Wilcoxon test (b - miR-31inv/Cre; C - for miR-31inv/WT; D – for miR-31inv/Cre and WT/WT); FC, fold change of control group; n, biologically independent mice (a-d) or human donors (e-h), as appropriate. FAC: fractional area change; myocardial cross-sectional area. Source data

**Extended Data Fig. 5:**
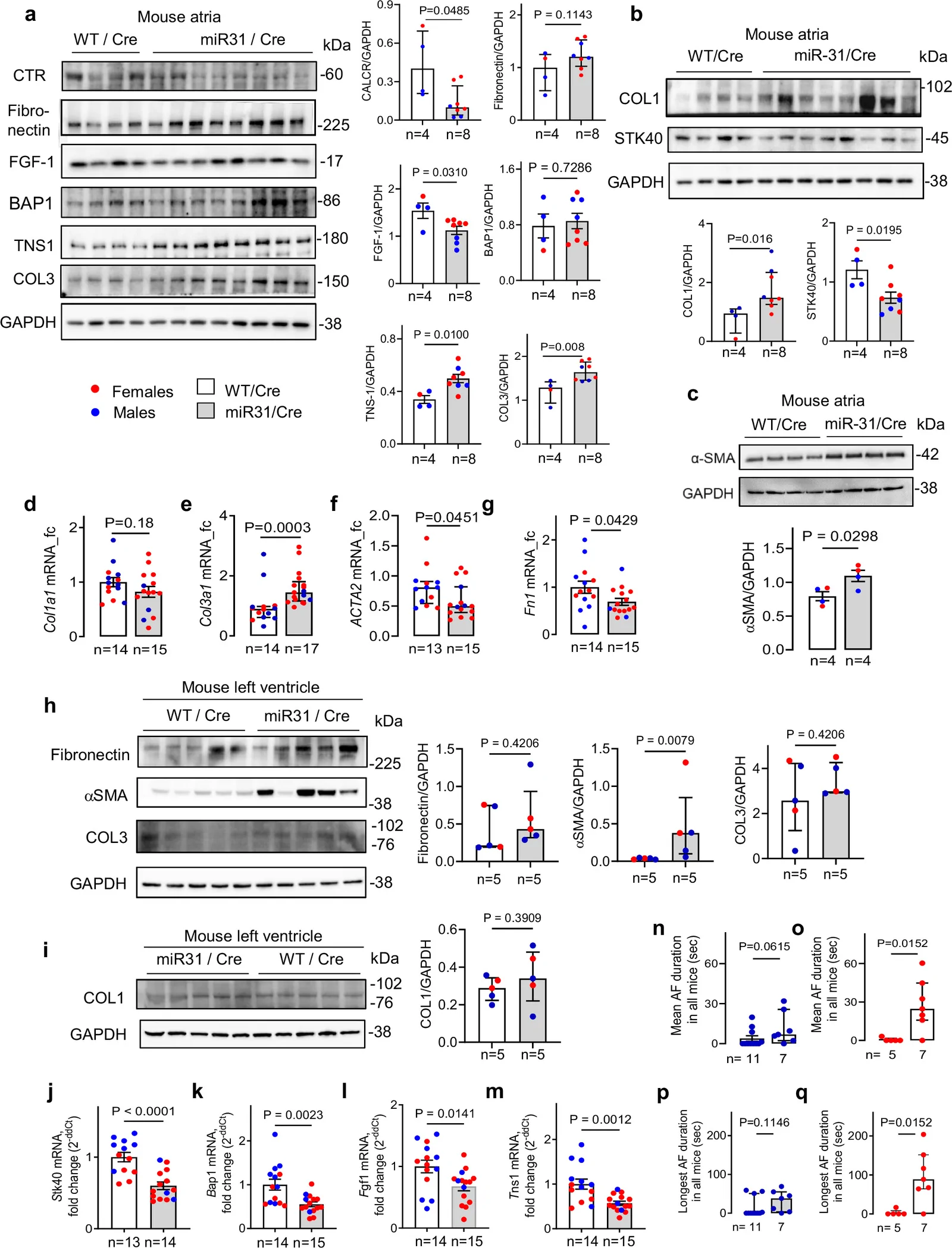
Validation of *miR-*31*-*5*p* targets in *miR-*31*/Cre* mice in the atrial and ventricular myocardium. (**a-c**) Effect of fibroblast-targeted miR-31 overexpression in female and male mice on protein content (Western blots) of CTR, fibronectin, FGF-1, BAP1, TNS1, collagen 3 (COL3), collagen 1(COL1), STK40 and αSMA in atrial tissue. (**d-i**) Effect of fibroblast-targeted miR-31 overexpression in female and male mice on mRNA levels of collagen 1, collagen 3, αSMA (encoded by *Acta*2) and fibronectin in atrial and left ventricular (**h, i**) tissue. (**j-m**) Effect of fibroblast-targeted miR-31*-*5*p* overexpression in mice on gene expression of selected previously validated *miR-*31*-*5*p* targets, *FGF-*1*, BAP*1*, STK*40, and *TNS-*1 in atrial tissue. (**n-q**) Mean and longest AF episode duration analysed by sex (males are in blue, females – in red). Data are expressed as mean ± SEM, except a (CALCR, COL3), b (COL1), f, c, h – expressed as median with IQR. P-values calculated by unpaired t test, except in a (CALCR, COL3), b (COL1), f, h, n-q - by Mann Whitney test; FC, fold change of control group; n, individual mice. Source data

Similar to humans, the mouse CTR (*Calcr*) mRNA also contains a conserved miR-31*-*5p binding site in its 3′ UTR (Fig. 2a). To test the effect of miR-31*-*5p−*Calcr* binding in vivo, we generated a tamoxifen-inducible fibroblast-targeted miR-31-transgenic mouse model (miR-31/Cre) using *Col*1*a*2*-*Cre-ER^T^ transgene (Fig. 2b,c and Extended Data Figs. 2 and 3a). A qPCR confirmed successful overexpression of mouse miR-31*-*5p in atrial and ear (a positive fibroblast-enriched control) tissue lysates (Fig. 2d,e) in miR-31/Cre mice versus control WT/Cre, or other relevant genotypes (WT/WT and miR-31/WT), in both female and male animals. The miR-31*-*3p strand, which does not have a binding site for CTR mRNA 3′ UTR, was also (although to a lesser extent) upregulated (by approximately 30% (Extended Data Fig. 3b) compared to approximately two-fold for miR-31*-*5p (Fig. 2f)) in mouse ACFs. Analysis of miR-31*-*5p expression in isolated atrial and ventricular cardiofibroblasts (CFs) and cardiomyocytes (CMs) showed a 2.5-fold and 1.6-fold increased expression in atrial CFs (*P* = 0.0032) and ventricular CFs (VCFs), although it did not reach statistical significance in VCFs (*P* = 0.062), with unchanged levels in atrial CMs (*P* > 0.999) and ventricular CMs (*P* = 0.700) (Fig. 2f,g). These results indicate the successful overexpression of the miR-31 transgene CFs. Neither miR-31 transgene expression nor tamoxifen administration affected ventricular function in mice in either genotype in males or females (Fig. 2h−m, Extended Data Fig. 4a−d and Supplementary Tables 3–5). Similar to hACFs, mouse miR-31 overexpression significantly decreased atrial CTR protein and atrial fibroblast mRNA (Fig. 2n and Extended Data Fig. 5a) compared to the control WT/Cre animals. This was associated with unchanged expression of the CTR ligand CT transcript (*Calca*) in mouse ACMs (Fig. 2o).

### Effect of miR-31*-*5p on hACF functions

**Fig. 3:**
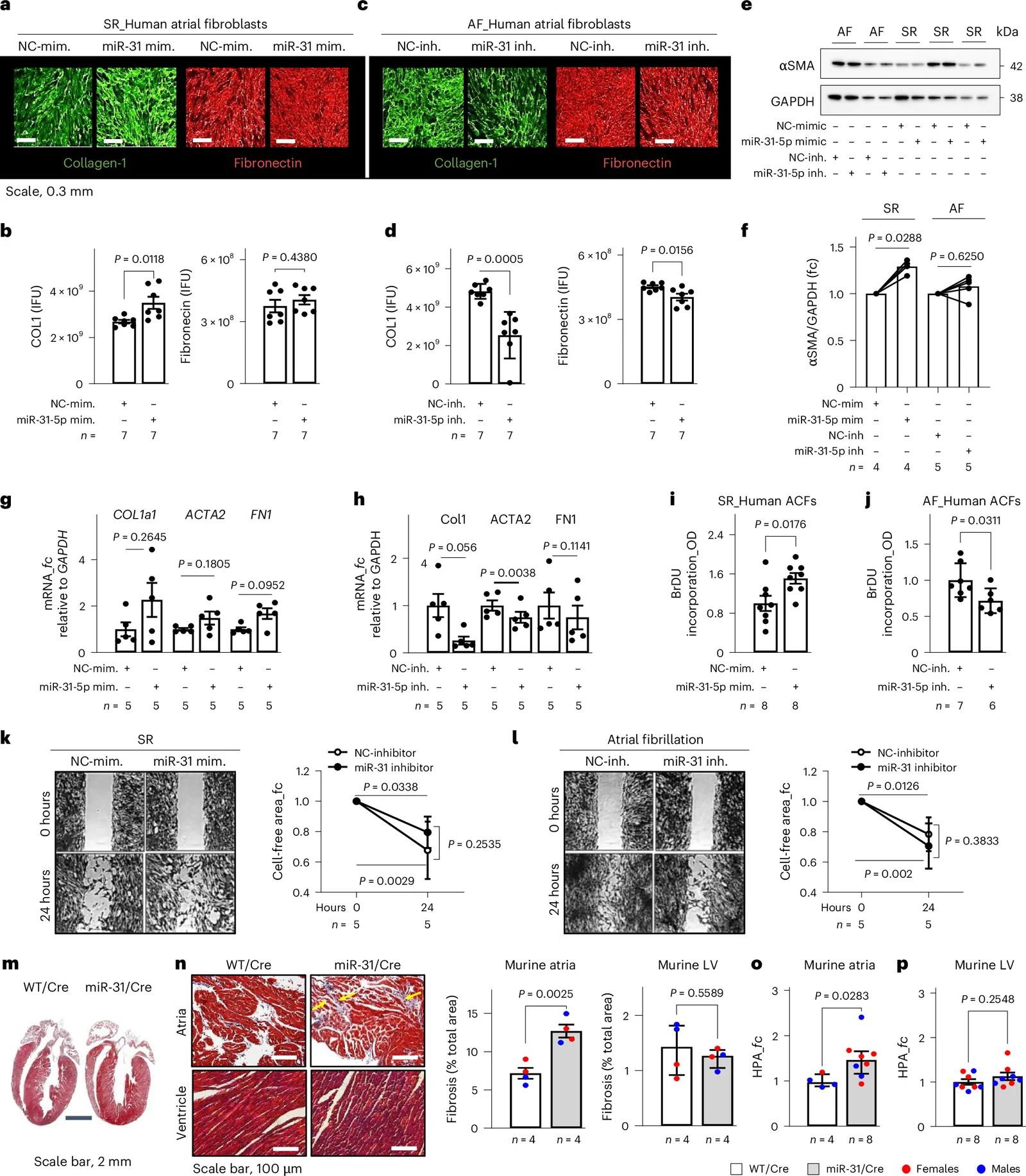
MiR-31 exacerbates profibrotic responses in human atrial fibroblasts and promotes atrial fibrosis in vivo in mice. **a**−**d**, Effect of miR-31 overexpression (**a**,**b**) or inhibition (**c**,**d**) on collagen and fibronectin production (scar-in-a-jar) in human ACFs obtained from control patients in SR (**a**,**b**) and with persAF (**c**,**d**). Scale bar, 0.3 mm. Representative images are shown from biologically independent donors (*n* = 7 per group) included in the corresponding quantification. **e**,**f**, Representative blots (**e**) and quantification (**f**) of αSMA protein in ACFs obtained from patients in SR and from patients with persAF transfected with miR-31 mimic and inhibitor, respectively. Immunoblot experiments were repeated independently twice. **g**,**h**, Gene expression (qPCR) of collagen-1 (*COL*1), αSMA (*ACTA*2) and fibronectin (*FN*1) in human ACFs transfected with *miR-*31 mimic and inhibitor in SR (**g**) and AF (**h**) groups, respectively. **i**−**l**, Effect of miR-31*-*5p mimic (mim), miR-31-5p inhibitor (inh) or respective negative controls (NC-mim, NC-inh) on cell proliferation (BrDU assay; **i**−**j**) and cell migration (scratch assay; **k**,**l**) in human ACFs from patients in SR (**i**, **k**) and from patients with persAF (**j**,**l**). **m**−**p**, Effect of induced miR-31 overexpression on atrial and ventricular fibrosis assessed by Masson’s trichrome staining (**m**,**n**) and HPA (**o**,**p**) in 12-week old miR-31/Cre and WT/Cre mice. Representative Masson’s trichrome images are shown from biologically independent mice included in the corresponding quantification. Data are expressed as mean ± s.e.m., except median with interquartile range in **o**. *P* values were calculated by unpaired *t*-test (**b**,**d**,**i**,**j**,**n**,**p**), paired *t*-test (**f**,**g**,**h**), Mann−Whitney test (**o**), Wilcoxon test and two-way repeated-measures ANOVA with Sidak’s test (**k**,**l**). FC, fold change of control group; LV, left ventricle; IFU, immunofluorescence unit; *n*, biologically independent donors or mice as appropriate; OD, optical density. *n* values are shown in the graphs. All statistical tests were two-sided unless otherwise stated. Source data

To evaluate the functional responses of hACFs to miR-31 overexpression or inhibition, we performed in vitro studies in these cells. ECM collagen-1 accumulation (assessed by scar-in-a-jar assay) was approximately 30% increased (*P* = 0.0118) by 72-hour overexpression of miR-31*-*5p mimic in SR hACFs (Fig. 3a,b). By contrast, inhibition of miR-31*-*5p with miR-31*-*5p inhibitor halved collagen-1 content in ACFs from patients with persAF (Fig. 3c,d). Similar effects were obtained in the presence of TGFβ1 stimulation (5 ng ml^−1^; Extended Data Fig. 4e,f). MiR-31*-*5p mimic had no effect on fibronectin protein in hACFs (Fig. 3a,b); by contrast, miR-31-5p inhibition reduced fibronectin in fibroblasts from patients with persAF (Fig. 3c,d). Levels of α-smooth muscle actin (αSMA) protein increased (*P* = 0.0288) in miR-31*-*5p-overexpressing SR hACFs, whereas it did not change after miR-31*-*5p inhibition in AF hACFs (Fig. 3e,f). Overexpression of miR-31*-*5p did not alter transcript expression of *COL*1, *ACTA*2 and *FN*1; miR-31-5p inhibition reduced *ACTA*2 (*P* = 0.0038) with a trend toward a reduction for *COL*1 (*P* = 0.056) and unchanged *FN*1 in hACFs (Fig. 3g,h). Cell proliferation was 50% higher (*P* = 0.0176) in miR-31*-*5p mimic SR hACFs (Fig. 3i) and reduced by approximately 29% (*P* = 0.0311) by miR-31*-*5p inhibition in AF ACFs (Fig. 3j). However, it remained unchanged in TGFβ1-activated hACFs in both miR-31*-*5p mimic and inhibitor-treated groups (Extended Data Fig. 4g,h), likely attributable to maximal ACF activation and proliferation response to TGFβ1. Neither miR-31*-*5p mimic (versus NC mimic; Fig. 3k) nor miR-31*-*5p inhibitor (versus NC inhibitor; Fig. 3l) affected the migration of hACFs. These results suggest that overexpression of miR-31*-*5p in vitro is sufficient to activate some key fibrotic responses (collagen-1 and αSMA production and cell proliferation) in hACFs, which, in the presence of persAF, are significantly suppressed by miR-31*-*5p inhibition.

### Upregulation of miR-31 in fibroblasts promotes structural and arrhythmogenic substrate in vivo

**Fig. 4:**
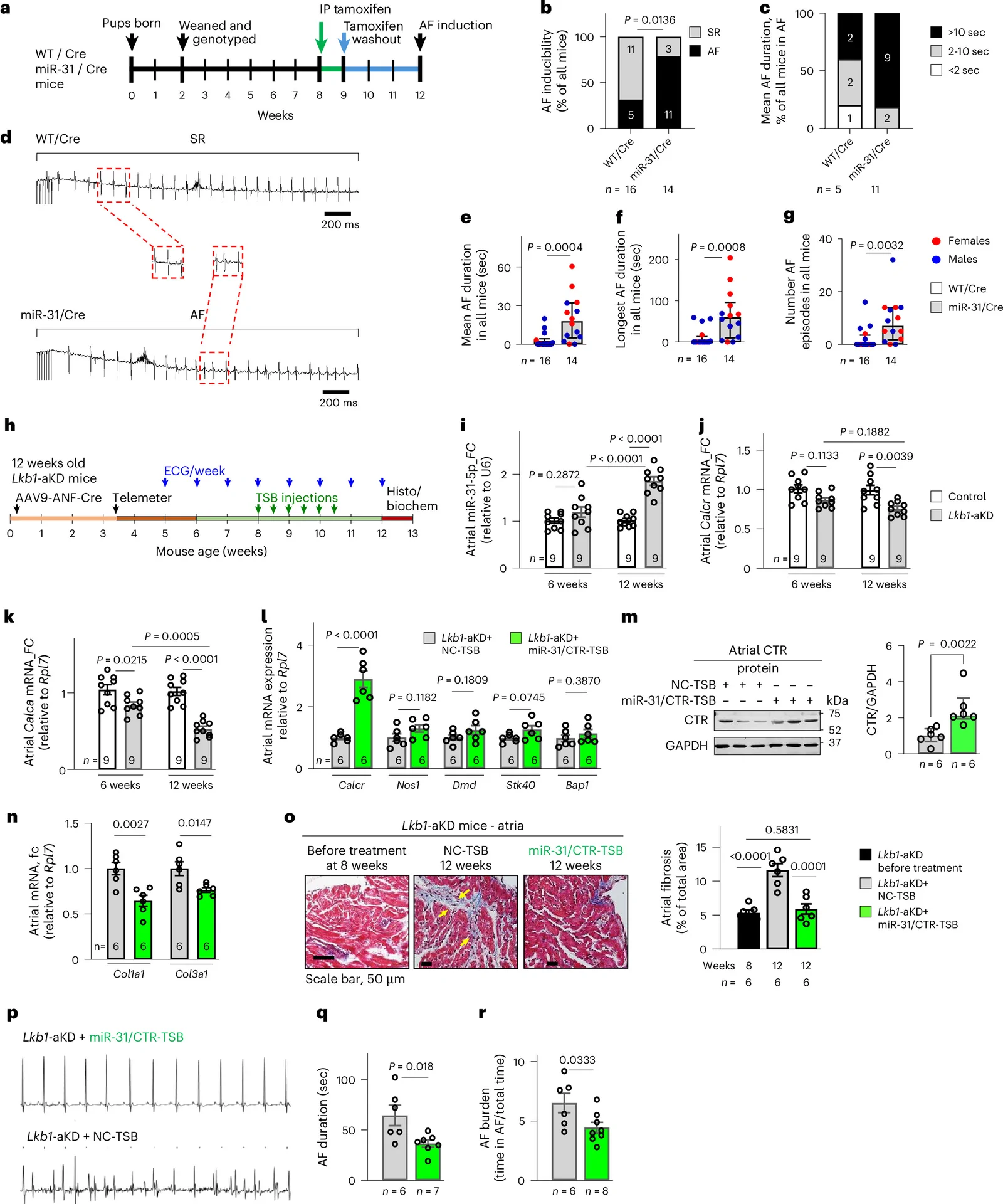
MiR-31 overexpression induces atrial fibrosis and increases AF susceptibility in vivo in mice prevented by targeted disruption of the miR-31−*Calcr* interaction. **a**, Experimental timeline in miR-31/Cre mouse studies. **b**−**g**, Atrial burst pacing-induced AF (**b**; % of all mice), the mean (**c**,**e**) and longest (**f**) AF episode duration and the number of AF episodes (**g**) in miR-31/Cre versus Cre/WT mice; example of the typical ECG recording is in **d**. **h**, Experimental timeline in 12-week-old *Lkb*1-aKD mice. **i**−**k**, Changes in atrial tissue miR-31*-*5p, CTR (*Calcr*) and CT (*Calca*) mRNA expression in *Lkb*1-aKD (versus controls) mice at 6 weeks and 12 weeks. **l**, Effect of miR-31/CTR-TSB on *CALCR* mRNA and previously validated miR-31 targets *Nos*1, *Dmd*, *Stk*40 and *Bap*1 in mouse atrial tissue. **m**, Effect of miR-31/CTR-TSB on atrial CTR protein in 12-week old *Lkb*1-aKD mice. **n**, Effect of miR-31/CTR-TSB or NC-TSB on atrial expression of collagens *Col*1 and *Col*3*a*1 in 12-week old *Lkb*1-aKD mice. **o**, Atrial fibrosis (by Masson’s trichrome staining) in 12-week old *Lkb*1-aKD mice treated with miR-31/CTR-TSB or NC-TSB control. Representative Masson’s trichrome images are shown from biologically independent mice included in the corresponding quantification. **p**−**r**, Representative ECG recordings (**p**), the AF episodes, the duration of the longest AF episodes recorded by ECG telemetry (**q**) and AF burden (**r**) in *Lkb*1-aKD mice treated with miR-31/CTR-TSB or NC-TSB. Data are expressed as mean ± s.e.m., except median with interquartile range in **e**−**g**. *P* values were calculated by unpaired *t*-test (**l**,**n**,**q**,**r**), except for the two-sided Fisher’s exact test (**b**), Mann−Whitney test (**e**−**g**,**m**), two-way ANOVA (**i**−**k**) and one-way ANOVA with Holm−Sidak test (**o**). FC, fold change of control group; IP, intraperitoneal; sec, seconds. All statistical tests were two-sided unless otherwise stated. *n* values are indicated in the figures and represent biologically independent mice; *n* values are shown in the graphs. Source data

To test whether the atrial fibrotic remodeling in persAF caused by the loss of CTR is secondary to miR-31 upregulation, we used the fibroblast-specific miR-31-overexpressing mice (Fig. 2). These mice (aged 12 weeks) displayed a doubled (*P* = 0.0025) amount of atrial (but not ventricular) fibrosis as assessed by Masson’s trichrome staining (Fig. 3m,n) and hydroxyproline assay (HPA) (*P* = 0.0283) (Fig. 3o,p) compared to the WT/Cre controls. These alterations were accompanied by approximately 40% increase in atrial protein levels of collagen-1 (*P* = 0.0162), collagen-3 (*P* = 0.0081) and αSMA (*P* = 0.0298) (Extended Data Fig. 5a−c), whereas mRNA was unchanged for collagen-1, increased for collagen-3 and reduced for αSMA (Extended Data Fig. 5d−f). Atrial fibronectin protein remained unchanged (despite decrease in its mRNA; *P* = 0.0429; Extended Data Fig. 5a,g). By contrast, left ventricular collagen-1, collagen-3 and fibronectin proteins remained unaltered, whereas αSMA increased (Extended Data Fig. 5h,i). Overexpression of miR-31 also caused a reduction in the mRNA (Extended Data Fig. 5j−m) and, in some cases, protein expression (with the exception of BAP1, which remained unchanged, and TNS1, which increased) expression in the atria of previously validated miR-31 targets, FGF-1, BAP1, STK40 and TNS1 (Extended Data Fig. 5a,b). Increased atrial fibrosis in mice was associated with a two-fold higher AF inducibility (*P* = 0.0136) (Fig. 4a,b), prolonged AF duration (Fig. 4c−f) (which was more pronounced in females (Extended Data Fig. 5n−q)) and more frequent AF episodes (Fig. 4g). As miR-31 overexpression in mice did not differ between males and females and had no correlation with sex, and there were no sex-dependent differences in heart rate, ventricular function or AF duration/number of AF episodes, we did not analyze male and female results separately in the downstream experiments. Our findings suggest that a clinically relevant two-fold upregulation of miR-31 in atrial fibroblasts (Fig. 2f) is sufficient to promote the formation of an AF substrate in vivo.

### *MiR-*31*-*5*p* alters the transcriptional program of hACFs

**Extended Data Fig. 6:**
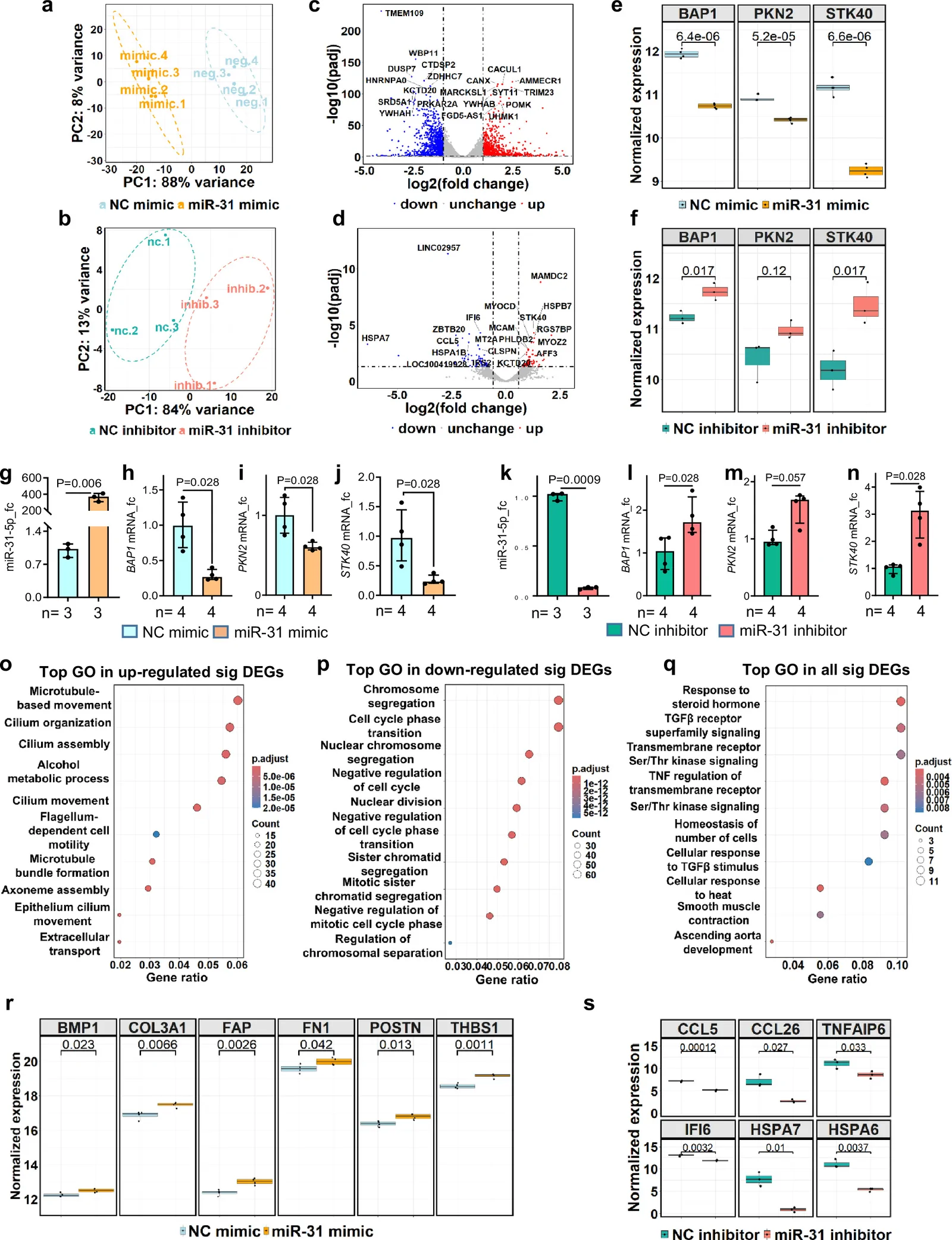
*MiR-*31*-*5*p* alters transcriptional programme in human atrial cardiofibroblasts (hACFs). (**a-b**) Principal component analysis of the variances between hACFs from SR patients transfected with *miR-*31*-*5*p* mimic (**a**) vs non-targeting control (NC) mimic, and AF-hACFs transfected with *miR-*31*-*5*p* inhibitor (**b**) vs NC-inhibitor. (**c-d**) Volcano plots showing the distribution of gene expression changes in miR-31 mimic (**c**) and miR-31 inhibitor (**d**) groups vs respective negative controls. Red dots - significantly up-regulated differentially expressed genes (DEGs); adjusted p-value < 0.05 and log2FoldChange (FC) > 1 (mimic) or 0.585 (inhibitor), blue dots - significantly down-regulated DEGs; grey dots - non-significant DEGs. (**e-f**) Expression (RNAseq) of BAP1, PKN2, and STK40 in miR-31-mimic (**e**) and –inhibitor (**f**) groups vs respective NC. (**g-n**) Effect of *miR-*31*-*5*p* mimic (**g**) and inhibitor (**k**) transfection on gene expression (qPCR) of previously validated *miR-*31*-*5*p* targets, *BAP*1*, PKN*2 and *STK*40 in human atrial cardiofibroblasts (hACFs) from control patients in SR (**g-j**) or persAF (**k-n**). (**o-q**) Dot plot of the top 10 enriched Gene Ontology (GO) biological processes (BPs) in significantly up-regulated (**o**) and down-regulated (**p**) DEGs comparing *miR-*31*-*5*p* mimic to NC-mimic, as well as all significant DEGs (**q**) comparing *miR-*31*-*5*p* inhibitor to NC-inhibitor. X-axis corresponds to gene ratio (that is, the number of genes in the input list annotated in the given GO term) and Y-axis corresponds to the name of enriched BP. Dot color indicates the adjusted p-value. (**r-s**) Normalized expression of profibrotic genes with significant difference between miR-31 mimic and NC mimic (r), or between miR-31 inhibitor vs NC-inhibitor (s). Ellipses in (a) indicates 95% confidence intervals based on multivariate T distribution; ellipses in (b) were estimated using the Khachiyan algorithm to demonstrate group clustering. Normalization (e, f, r, s) was performed with DESeq2 (v1.44.0) using size factors, followed by log2 transformation. Data in (c, d) and (o-q) are adjusted for multiple testing using the Benjamini-Hochberg FDR method. GO analysis used clusterProfiler (v4.12.3) with the human genome background. P-values were calculated by unpaired t test (e, f, r, s), otherwise Mann-Whitney (g-n); fc, fold change of control group; n, individual donors. Source data

To assess whether miR-31*-*5p alters other potential target genes and the broader transcriptional program of hACFs, control SR ACFs were transfected with miR-31*-*5p mimic, and AF ACFs with miR-31*-*5p inhibitor (or respective NCs), and submitted for bulk RNA sequencing (RNA-seq) (Extended Data Fig. 6). Principal component analysis revealed clustering of different groups by treatment and a distinct pattern in differentially expressed genes (DEGs) between miR-31*-*5p mimic and inhibitor versus NC mimic/NC inhibitor groups (Extended Data Fig. 6a−d). Although miR-31*-*5p overexpression significantly upregulated 1,023 genes and downregulated 1,009 genes, miR-31*-*5p inhibition upregulated 70 genes and downregulated 70 genes (Extended Data Fig. 6c,d). RNA-seq and qPCR confirmed concordant changes in previously reported miR-31 gene targets *BAP*1, *PKN*2 and *STK*40 (refs. ^23,24^) in hACFs transfected with *miR-*31*-*5*p* mimic and inhibitor (Extended Data Fig. 6e−n). DEG pathway analysis found that miR-31*-*5p overexpression leads to the activation of microtubule movement/formation as well as pathways involved in cilium organization and assembly (Extended Data Fig. 6o) while inhibiting cell cycle and nuclear chromosome organization and function (Extended Data Fig. 6p). These transcriptional changes may contribute to the miR-31-mediated fibrotic responses in AF hACFs^25^. By contrast, miR-31*-*5p inhibition in AF hACFs alters genes involved in TGFβ, TNF and steroid hormone signaling (Extended Data Fig. 6q). Notably, miR-31*-*5p mimic treatment elevated the expression of several fibrosis-relevant genes (for example, *BMP*1, *COL*3, *FAP*, *FN*1, *POSTN* and *THBS*1; Extended Data Fig. 6r), the relevance of which should be explored in future work. MiR-31*-*5p inhibition was also associated with suppressed inflammation-related DEGs *CCL*5, *CCL*26, *TNFAIP*6, *IFI*6 and *HSPA*6/*HSPA*7 (Extended Data Fig. 6s). The concordant changes between miR-31*-*5p and these DEGs suggest an indirect effect of miR-31*-*5p on them.

### Selective disruption of miR-31*-*5p−*Calcr* interaction reverses atrial fibrosis and arrhythmia in vivo

Locked nucleic acids (LNAs) are modified nucleic acids with enhanced binding affinity for their target RNA^26^. Using miRCURY LNA-miR-TSB in vivo to selectively block the miR-31*-*5p−*Calcr* interaction, we tested causality among the miR-31*-*5p−*Calcr* interaction, atrial fibrosis and AF development in the previously validated *Lkb*1-aKD mouse model of spontaneous AF (Fig. 4h)^13,27^. Similar to the changes observed in patients with persAF^13^ (Fig. 1b–e), the *Lkb*1-aKD mice displayed almost doubled levels of miR-31*-*5p (Fig. 4i) and reduced (by approximately 30%) *Calcr* mRNA and *Calca* mRNA (Fig. 4j,k) (versus *Lkb*1^fl/fl^ animals) at 12 weeks (and, for *Calca*, as early as 6 weeks) of age. Thus, *Lkb*1-aKD mice represent a useful experimental model in the context of the AF-associated activation of miR-31*-*5p−CTR signaling.

We initially injected 8-week-old *Lkb*1-aKD mice with miR-31/CTR-TSB or respective NC-TSB and monitored structural remodeling (atrial fibrosis) and AF burden/duration for 4 weeks. As 8-week-old mice do not exert established fibrosis and structural remodeling, they were used to test the preventive value of the TSB treatment on structural remodeling and arrhythmogenesis. We observed that miR-31/CTR-TSB treatment rescued atrial CTR mRNA (*P* = 0.0001) and protein (*P* = 0.0076) (Fig. 4l,m) without changes in other previously validated targets of miR-31 (*Nos*1, dystrophin (*Dmd*), *Stk*40 and *Bap*1; Fig. 4l), thus confirming the specificity of the TSB approach to disrupt the miR-31*-*5p−*Calcr* interaction. Compared to NC-TSB, the miR-31/CTR-TSB treatment suppressed expression of the atrial collagen-1 (by approximately 40%, *P* = 0.0027) and collagen-3 (by approximately 25%, *P* = 0.0147) (Fig. 4n), prevented the development of atrial fibrosis (*P* = 0.0001) assessed with Masson’s trichrome stain (Fig. 4o) and reduced AF duration by approximately 45% (*P* = 0.018) and burden by approximately 32% (*P* = 0.0333) (Fig. 4p−r). Therefore, selective blockade of miR-31−*Calcr* binding is sufficient to preclude atrial fibrogenesis and control atrial arrhythmogenesis in vivo in mice.

**Fig. 5:**
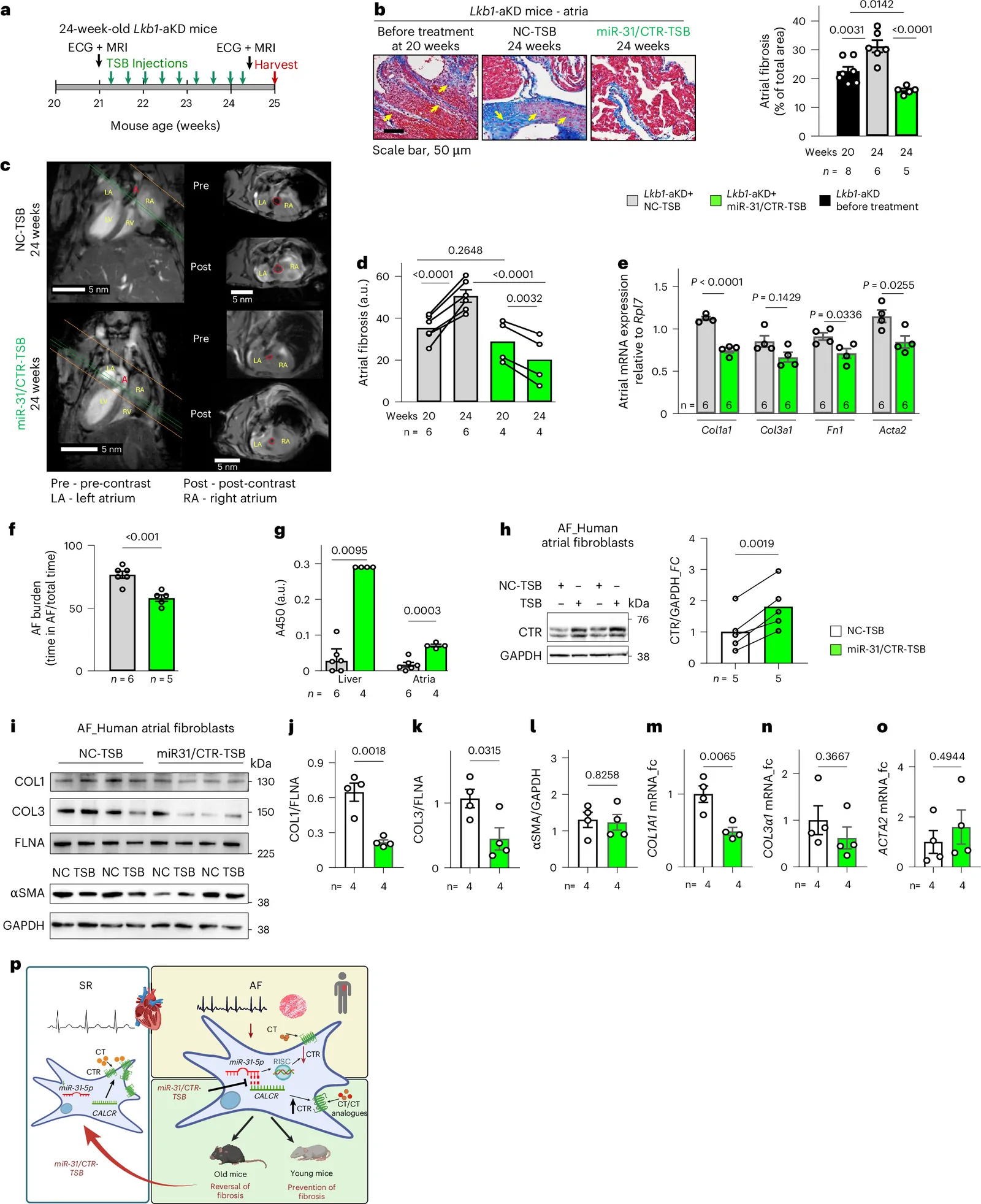
Selective blockade of the miR-31−*Calcr* interaction with miR-31/CTR-TSB reduces established atrial fibrosis and arrhythmogenesis in vivo in mice. **a**, Experimental timeline in 24-week old *Lkb*1-aKD mice. **b**, Atrial fibrosis (by Masson’s trichrome staining) in 24-week-old *Lkb*1-aKD mice treated with miR-31*-*5*p*/CTR-TSB or NC-TSB control. Representative Masson’s trichrome images are shown from biologically independent mice included in the corresponding quantification. **c**,**d**, Effect of miR-31-5p/CTR-TSB or NC-TSB on atrial fibrosis (by MRI, assessed in the same animals at 20 weeks and 24 weeks of age, pre-TSB and post-TSB treatment, respectively) in *Lkb*1-aKD mice. Representative MRI images from the 24-week post-TSB treatment; the orange and green lines indicate the area where the slices were acquired and the short axis, respectively; red circle outlines aorta. Representative MRI images are shown from biologically independent mice included in the corresponding analyses. **e**, Gene expression (by qPCR) of collagen-1 (*Col*1*a*1), collagen-3 (*Col*3*a*1), fibronectin (*Fn*1) and αSMA (*Acta*2) in atrial tissue of mice after miR-31*-*5p/TSB (or NC-TSB). **f**, AF burden in *Lkb*1-aKD mice assessed between 20 weeks and 24 weeks of age. **g**, Hybridization assay for the post-TSB treatment levels of miR-31*-*5p/CTR-TSB (versus NC-TSB) in mouse liver (positive control) and atrial tissue in 24-week-old *Lkb*1-aKD mice. **h**, Effect of miR-31*-*5p/CTR-TSB or NC-TSB on CTR protein in human ACFs. Representative immunoblots are shown from biologically independent donor samples included in the corresponding quantification. **i**−**o**, Effect of miR-31*-*5p/CTR-TSB or NC-TSB on protein and mRNA levels of selected fibrotic markers, collagen-1 (COL1), collagen-3 (COL3) and αSMA (normalized to filamin A, FLNA or GAPDH as annotated) in ACFs from patients with persAF. Representative immunoblots are shown from biologically independent donor samples included in the corresponding quantification. **p**, Diagram depicting the main findings of the study. Data are expressed as mean ± s.e.m., except median and interquartile range (**g**, liver). *P* values were calculated by unpaired *t*-test, except for one-way ANOVA (**b**), two-way repeated-measures ANOVA (**d**), Mann−Whitney test (**g**, liver) or paired *t*-test (**h**). FC, fold change of control group; *n* values are indicated in the figures and represent biologically independent human donors (**h**−**o**) or mice (**b**−**g**), as appropriate; *n* values are shown in the graphs. All statistical tests were two-sided unless otherwise stated. Schematic in **p** created in BioRender; Ma, S.C. https://biorender.com/fmy4cte (2026). Source data

Although the prevention of atrial fibrosis was achievable in younger mice by therapy beginning before the development of fibrosis, we next questioned whether selective blockade of miR-31-5p−*Calcr* binding could exert a therapeutic effect on established atrial fibrosis (commonly observed in patients with persAF) and arrhythmia. Thus, we subjected *Lkb*1-aKD mice at 20.5 weeks of age that displayed approximately 26% baseline atrial fibrosis, similar to the level observed in patients with persAF^28^, to a 3-week treatment with miR-31/CTR-TSB, or NC-TSB (25 mg kg^−1^, intraperitoneal, three times per week) (Fig. 5a). Atrial arrhythmias and fibrosis were monitored at baseline (20 weeks) and after TSB treatment (24 weeks) by electrocardiogram (ECG) and magnetic resonance imaging (MRI) (using a collagen-1-binding gadolinium-diethylenetriamine pentaacetic acid dye, EP3533), respectively^29^. Baseline atrial fibrosis did not differ significantly between groups; however, it decreased by approximately 30% (compared to the pre-treatment 20-week fibrosis levels) after TSB treatment (confirmed by Masson’s trichrome staining; Fig. 5b), whereas, in the NC-TSB control group, atrial fibrosis progressed by approximately 46% above the 20-week baseline fibrosis (*P* < 0.0001) (Fig. 5c,d). The miR-31/CTR-TSB-mediated reversal of atrial fibrosis was accompanied by a reduction in atrial mRNA expression of collagen-1 (by 33%, *P* = 0.0001), fibronectin (by 22%, *P* = 0.0336) and αSMA (by 26%, *P* = 0.0255) with unchanged collagen-3 (Fig. 5e) in TSB-treated (versus NC-TSB) mice. ECG revealed reduced AF burden (by approximately 16%; *P* = 0.033) in miR-31/CTR-TSB-treated mice (Fig. 5f). A sandwich hybridization assay with a biotinylated LNA capture probe showed efficient delivery of the miR-31/CTR-TSB to the atrial myocardial, with 4.2-fold and 8.1-fold increase (versus NC) in the atria and liver, respectively (Fig. 5g)^30^. The miR-31/CTR-TSB treatment had no effect on blood pressure, heart rate or cardiac contractility (Supplementary Table 5). In hACFs, the TSB-mediated selective inactivation of miR-31−*Calcr* binding approximately doubled CTR protein (*P* = 0.0125) (Fig. 5h) and suppressed mRNA and protein levels of collagen-1 (by approximately 68%, *P* = 0.0018, and 51%, *P* = 0.0065, respectively) and reduced collagen-3 protein (but not mRNA) by 56% (*P* = 0.0315) without changes in αSMA expression (Fig. 5i−o). These observations confirm that the actions of miR-31/CTR-TSB in the in vivo mouse model are relevant to human atrium, and they demonstrate that selective deactivation of the miR-31−CTR signaling axis with miR-31/CTR-TSB reverses the established atrial fibrosis in vivo.

## Discussion

In this study, we identified miR-31*-*5p as an upstream regulator of CTR signaling, whereby miR-31*-*5p binds the CTR 3′ UTR to enhance its degradation/reduce translation and suppress CTR expression in human atrial fibroblasts and promotes atrial fibrosis and arrhythmogenesis in vivo. Activation of this signaling cascade in the presence of heart disease, such as AF, or in an miR-31-transgenic mouse model, induces fibrotic fibroblast activity leading to excessive atrial fibrogenesis and increased arrhythmia susceptibility. Patients with persAF, characterized by established atrial fibrosis and decreased CTR expression^13^, have doubled miR-31*-*5p levels and increased miR-31*-*5p−*CALCR* interaction in ACFs. Animal studies showed that selective disruption of this signaling axis, with an miR-31*-*5p/CTR-TSB, was sufficient to prevent new (in 12-week-old mice) and reverse advanced (in 24-week-old mice) atrial fibrosis and arrhythmogenesis in vivo. These findings are schematically summarized in Fig. 5p.

While previous studies, including our earlier work, characterized the CT−CTR paracrine system in atrial remodeling^13^, the present study provides three important insights. First, we identify miR-31-5p as a key upstream regulator of CTR in human atrial fibroblasts. Second, we show that selective blockade of miR-31-5p−CTR binding not only prevents but also reverses established atrial fibrosis in vivo. Third, these findings provide proof of concept for a selective RNA-based therapeutic strategy targeting atrial fibrosis. Previous work showed that miR-181b antagonism reduces atrial fibrosis and AF vulnerability through an endothelial-to-mesenchymal transition pathway^31^. Our present findings complement this concept by identifying the fibroblast-specific miR-31-5p−CTR axis and demonstrating that selective TSB can promote regression of established atrial fibrosis and reduce arrhythmia burden in vivo.

A recently discovered endogenous, cardio-endocrine CT signaling system in human atrial myocardium controls atrial fibrosis and arrhythmogenesis^13^. Impaired CT−CTR signaling, as in patients with persAF, promotes atrial fibrogenesis and the formation of an arrhythmogenic substrate. Although patients with persAF have suppressed CT production and CTR expression/internalization, they are unlikely to benefit from clinically approved CT analogues unless CTR expression and function is restored. Furthermore, using CT analogues may further reduce cellular CTR surface expression through ligand-induced receptor internalizationm, which we previously observed in human atrial fibroblasts^13^, a phenomenon commonly observed with G-protein-coupled receptors^32^. Thus, in light of the therapeutic potential of this signaling system for atrial fibrosis and arrhythmia, we searched for upstream mechanisms regulating CTR abundance in hACFs. We found that both right and left ACFs from patients with persAF have doubled levels of miR-31*-*5p (associated with aging and shown to play a role in ischemic and coronary heart diseases)^33,34^ that negatively correlated with CTR mRNA (*CALCR*) expression, pointing toward a potential regulatory role for miR-31*-*5p on *CALCR* abundance. This was supported by the results of the reporter assay, which demonstrated changes in CTR gene/protein in hACFs after activation or suppression miR-31*-*5p and by the confirmation of endogenous miR-31*-*5p−CTR mRNA binding by RISC pulldown in human AF ACFs. Both miR-31*-*5p inhibition and selective disruption of miR-31*-*5p−CTR binding with TSB in vitro in human ACFs was sufficient, through restoring CTR expression, to repress some key fibrotic responses of ACFs—for example, collagen production and proliferation.

Previous studies suggested transcriptional regulation of CTR expression^35^. However, CTR mRNA expression is unchanged in patients with AF^13^, so transcriptional alterations are unlikely to play a major role in AF hACFs in the intact human heart, despite *miR-*31*-*5*p*-mediated changes in CTR mRNA expression in the in vitro studies using supra-physiological miR-31*-*5p levels. This discrepancy is likely due to a more complex endogenous environment and potential compensatory feedback in the endogenous myocardial environment of patients with AF.

Although we found that miR-31*-*5p regulates *CALCR* abundance, it is common for a single gene to be regulated by multiple miRs^36^; hence, CTR abundance in AF ACFs might be controlled by other miRs. We tested the potential role of three other miRs previously documented to play a role in persAF that had a high predicted score for binding to CTR 3′ UTR, but we found no regulatory effects of those miRs on CTR. Notably, the logistic regression analysis showed that miR-31*-*5p was significantly associated with a higher risk of AF (Supplementary Table 6; optical density = 3.02, *P* = 0.025), suggesting that the upregulation of miR-31 in AF is not influenced by other confounding factors, highlighting its specific contribution to atrial fibrosis and AF pathogenesis.

In vivo studies confirmed the miR-31*-*5p-mediated regulation of CTR in fibroblast-targeted miR-31-overexpressing mice with a clinically relevant approximately 2.5-fold (similar to that observed in patients with persAF) miR-31*-*5p upregulation. These mice had raised expression of both miR-31*-*5p and miR-31*-*3p; however, as the latter has no binding site for CTR 3′ UTR, it was not studied further. Furthermore, the ability of miR-31-5p/CTR-TSB treatment to reverse atrial fibrosis and arrhythmogenesis indicates that the miR-31-5p–CTR interaction is a major driver of these phenotypes, despite the concomitant increase in miR-31-3p expression. Notably, the changes observed in these mice were primarily driven by miR-31*-*5p produced by atrial (not ventricular) CFs, as ventricular ACF fibrotic markers and left ventricular function did not differ between groups. Consistent with previous reports^17,18^, we observed greater levels of miR-31*-*5p in the atria, whereas it was barely detectable in ventricular myocardium (10-fold increase in atrial CFs versus ventricular CFs). Accordingly, we found that fibroblast-targeted miR-31*-*5p overexpression in mice caused an increase in atrial fibrosis associated with a significant prolongation of arrhythmic episodes and increased AF susceptibility. These changes seem to be independent of sex (we observed no differences between males and females) and developed in the absence of left atrial dilatation or changes in left ventricular systolic or diastolic function in miR-31/Cre mice. Therefore, a fibroblast-selective miR-31 expression increase, similar to that seen in patients with persAF, is alone sufficient to promote structural, fibrotic remodeling that translates into enhanced arrhythmia-maintaining substrate and vulnerability to AF. Additional sex-stratified analyses indicated that the increase in AF duration was more pronounced in female mice, although the present study was not designed or powered to investigate sex-specific mechanisms. Notably, miR-31*-*5p has also been shown to regulate the electrical properties of atrial CMs in persAF^17,18^; therefore, it is plausible that miR-31*-*5p (via distinct gene targets) represents a common master driver of electrical and structural remodeling of the atrial myocardium in AF.

To test the relevance of the miR-31*-*5p−CTR mRNA interaction to atrial remodeling, we utilized a previously characterized *Lkb*1-aKD mouse model of spontaneous AF^27^. These mice (similar to patients with AF) have two-fold upregulated miR-31*-*5p and reduced CTR expression and, thus, represent a clinically relevant (in terms of miR-31-5p−CTR signaling) model. Selective blockade of the miR-31*-*5p−*Calcr* interaction in mouse atria with miRCURY LNA^26^ miRNA Power miR-31/CTR-TSB, which was successfully delivered to the atrial myocardium, specifically (without altering the expression of other miR-31 targets) increased atrial CTR expression, confirming an endogenous miR-31*-*5p−CTR interaction in vivo. Furthermore, 3-week TSB therapy in young (8-week-old) mice fully prevented the development of atrial fibrosis and significantly reduced both AF burden and duration. Although young mice displayed an atrial fibrotic burden (approximately 6% of total area) similar to that of non-transgenic age-matched mice, this does not reflect the typical clinical presentation of patients with established structural remodeling, like in persAF, who typically exhibit advanced (approximately 22%) atrial fibrosis^28^. Thus, we administered either miR-31/CTR-TSB or NC-TSB (NC oligonucleotide) to 20-week-old mice with established (approximately 26%) atrial fibrosis using a higher frequency treatment regimen (25 mg kg^−1^ per injection intraperitoneally, three times per week for 3 weeks). Pre-treamtent and post-treatment (at 20 weeks and 24 weeks) MRI and histological assessment with Masson’s trichrome staining showed a reversal (by approximately 30% below the 20-week baseline) of atrial fibrosis in *Lkb*1-aKD mice in vivo. As in mice, miR-31/CTR-TSB treatment of human AF ACFs suppressed production of some key fibrotic markers (for example, collagen-1/collagen-3). Hence, miR-31/CTR-TSB administration might offer a new approach to reverse advanced structural remodeling in the atrial myocardium, such as that encountered in patients with persAF. However, these findings should be interpreted in the context of the specific fibrosis phenotype studied. The *Lkb*1-aKD model is characterized predominantly by diffuse interstitial atrial fibrosis, and, therefore, our data demonstrate reversal of established interstitial remodeling rather than all forms of atrial fibrosis. Whether miR-31/CTR-TSB therapy can similarly reverse other fibrosis patterns, including more advanced replacement fibrosis or scar-related remodeling, remains to be established in future studies. In addition, the *Lkb*1-aKD mouse represents a specific genetic model of atrial cardiomyopathy and, therefore, does not fully capture the heterogeneous and multifactorial mechanisms that contribute to AF in patients. Although this model reproduces several hallmarks of persAF, including atrial fibrosis, spontaneous atrial arrhythmias and activation of the miR-31-5p−CTR pathway, it is restricted to the atria and does not incorporate potential ventricular influences on atrial remodeling. Consequently, further studies in complementary models of AF will be required to determine the generalizability of these findings. Several translational challenges also remain. In the present study, miR-31/CTR-TSB was administered systemically rather than by cell-type-specific delivery and required repeated dosing to achieve efficacy. In addition, the durability of the anti-fibrotic response after treatment cessation remains unknown. Future studies will be required to optimize delivery strategies, define the duration of therapeutic benefit and determine whether fibrosis recurs after treatment withdrawal.

In a canine model of heart-failure-induced atrial fibrosis and AF, complete reversal of heart failure does not lead to reversal of fibrosis and the AF substrate^37^, so active therapy is likely needed to decrease already established fibrosis. Given that miR-31 upregulation in persAF is atrial specific^17^, such therapy may help to circumvent off-target effects on ventricular myocardium, a common limitation of many current AF therapies^38^. Furthermore, use of selective miR-mRNA-TSB agents may help to minimize the concerns of off-target effects presented by global miR inhibition^38^—for example, changes in multiple (including physiologically important) miR-31*-*5p targets identified by RNA-seq. Encouragingly, miR-31/CTR-TSB administration had no effect on previously identified miR-31*-*5p targets (for example, *Nos*1, *Dmd*, *Stk*40 and *Bap*1), confirming the high selectivity of the miR-31*-*5p/CTR-TSB approach in hACFs and mouse atrial myocardium. Notably, 3-week pulse therapy with miR-31/CTR-TSB (25 mg kg^−1^ per injection, three injections per week; compared to NC-TSB) was effective not only in preventing fibrosis from developing but also in reversing (by 30%) advanced atrial fibrosis in vivo in 24-week-old *Lkb*1-aKD mice with established atrial remodeling. However, these findings should be interpreted in the context of the specific conditional *Lkb*1-aKD model used, which reflects a defined genetic mechanism of atrial remodeling. Further studies in additional models and settings will be required to determine the broader applicability and translational potential of this approach.

Although our present study demonstrates that selective blockade of miR-31-5p−CTR interaction restores CTR expression and reverses established atrial fibrosis, we did not directly test combined CT analogue therapy. Previous work from our group showed that enhancement of CT signaling suppresses atrial fibrogenesis and arrhythmia through CTR-dependent mechanisms in human atrial fibroblasts and mouse models of AF^13^. Therefore, whether CT replacement provides additional benefit beyond restoration of CTR expression remains to be determined. In addition, the durability of TSB-mediated anti-fibrotic effects after treatment cessation remains unknown. Longer-term studies will be required to determine whether fibrosis recurs after therapy withdrawal and to define the timecourse of any recurrence. Given that aging-related/senescence-related processes^20–22^, as well as ischemic^33^ or coronary heart disease^34^, can lead to miR-31 upregulation, it is conceivable that miR-31 upregulation in AF might be secondary to the underlying cardiac pathology, or aging, rather than driven by AF itself. However, the latter cannot be excluded, as miR-31 rises in *Lkb*1-aKD mice that display aging-related remodeling localized to atrial (but not ventricular) myocardium. Intriguingly, both upregulation of miR-31*-*5p and impaired CT−CTR signaling are also linked to the age-associated processes in bones and joints: osteoporosis and osteoarthritis^20,39–41^. Thus, the role of the miR-31*-*5p−CTR axis and the miR-31*-*5p−CTR blocking strategies may be of relevance and interest to the (patho)physiology of the skeletal system.

We also noted that a prominent reduction in atrial fibrosis was associated with a relatively modest decrease in AF burden in 24-week-old mice. This might be due to the relatively short (3 weeks) TSB treatment period or the insufficient CTR activation by its ligand CT, which is progressively suppressed in *Lkb*1-aKD mice. The results presented here, taken in the context of the existing evidence, demonstrate the specificity of the miR-31/*Calcr*-TSB approach and position *Calcr* as an important miR-31-5p downstream target in the context of fibrosis and arrhythmia. Together, these data suggest enrichment of this miR in the atrial myocardium compared to the left ventricle. Furthermore, given that miR-31-5p is highly enriched in the atrial myocardium, strategies to manipulate miR-31-5p can offer primarily atrial-targeted therapeutic options, allowing to circumvent the non-specific effects on the ventricular myocardium—a serious limitation of the current anti-arrhythmic drugs.

In summary, we have identified an miR-31*-*5p−*CALCR*-mRNA regulatory mechanism upstream of the cardiac CT system in human atrial myocardium. Furthermore, we found that advanced atrial fibrosis, commonly seen in persAF, is characterized by hACFs enriched for miR-31*-*5p, which, via binding to the *CALCR* 3′ UTR, activates fibroblast proliferation and collagen production. Selective disruption of miR-31*-*5p−*Calcr*-mRNA binding reversed atrial fibrosis and decreased AF burden in vivo in a clinically relevant model of progressive atrial fibrosis and arrhythmia. Thus, selective blockade of the miR-31*-*5p−CTR-mRNA interaction with miR-31/*Calcr*-TSB treatment may offer a safer, atrial-selective strategy to restore CT signaling and reestablish control over atrial fibrogenesis and prevent associated arrhythmia susceptibility in patients with a history of AF. MiR-31/CTR-TSB offers a target-selective approach in miR therapeutics, which will have to be further validated in human AF in clinical trials.

## Methods

### Patient cohorts

Investigations using human samples were approved by the South Central-Berkshire B Research Ethics Committee (REF: 18/SC/0404). All patients gave informed written consent. A total of 145 patients (70 control patients in normal SR and 75 with persAF, duration more than 7 days) were included in the study; all patients underwent cardiac surgery (coronary artery bypass or valve repair/replacement) in the John Radcliffe Hospital, Oxford. Detailed patient characteristics are shown in Supplementary Table 2. RAA and LAA biopsies were collected during cardiac surgery, mostly before or shortly after (for LAA samples) cardiopulmonary bypass, and immediately processed for cell isolation (described below) or snap frozen until use in subsequent experiments (for example, mRNA expression and immunoblotting). Age and sex distributions of the study participants are shown in Supplementary Table 2. Participants did not receive financial compensation for participation in this study. Individual experiments were performed using subsets of the overall cohort, with the corresponding sample sizes indicated in the figures and figure legends. Correlation analyses reported in Fig. 1f,g were performed only in samples with paired miR-31-5p and *CALCR* measurements available from the same biological specimen, which resulted in smaller sample sizes than the corresponding expression analyses.

### Animals

Animal breeding, handling and experimental work were carried out in two centers—Baylor College of Medicine (USA) and the University of Oxford (UK)—according to the local Home Office standards. All animal work was performed in accordance with the UK Home Office Animals (Scientific Procedures) Act 1986 incorporating Directive 2010/63/EU of the European Parliament and in accordance with National Institutes of Health (NIH) guidelines. Animals were maintained under a 12-hour light/dark cycle (06:00−18:00) at controlled temperature (68−72 °F; 20−22°C) and approximately 50% humidity. The total number of mice used in all animal studies was 175, consisting of 66 (35 males and 31 females) *Lkb*1-aKD and 109 (51 males and 58 females) miR-31/Cre mice. Animals of both sexes were used in the experiments. Mice were age matched and sex matched between experimental groups. The age and genotype of animals used in individual experiments are indicated in the corresponding Methods sections and figure legends. Where sex-specific analyses were performed, male and female data are presented separately in the relevant figures.

### Generation of *Lkb*1-aKD mice

*Lkb*1^fl/fl^ mice (The Jackson Laboratory, strain no. 014143; C57BL/6J background) were used to generate the atrial-specific *Lkb*1-aKD model. In brief, AAV9-ANF-Cre was injected into 2−5-day-old *Lkb*1^fl/fl^ pups (5 × 10^11^ genome copies, subcutaneous injection), generating atrial-specific knockdown of *Lkb*1 (*Lkb*1-aKD). Based on our previous studies^13,27^, these mice develop ectopic activities from week 5−6 that can be detected by surface ECGs. A 15-minute surface ECG was performed weekly to confirm ectopic activity and/or the onset of AF (from week 8 onwards). These mice were then treated with NC or miR-31/CT-TSB at either 8 weeks or 20 weeks (for the MRI cohort). Mice were studied at either 12 weeks of age (young cohort) or 24 weeks of age (MRI cohort), as indicated in the corresponding figure and figure legend.

### Generation of miR-31/Cre mice

The miR-31/Cre mice were generated using a PhiC31 integrase-mediated cassette exchange system (Extended Data Fig. 2). Pre-miR-31-Inv transgenic mice were generated using PhiC31 integrase that catalyzes unidirectional recombination between specific sequences and is used for insertion of transgenic constructs into defined genomic locations^42^. Consequently, pre-miR-31-Inv heterozygous mice carry a single copy of the pre-miR-31-Inv transgene at the *Gt(ROSA*26*)Sor* locus, which has benefits including that miR-31 dosage is more predictable and closer to endogenous levels and that dosage can be doubled in homozygous animals if needed. In addition, this approach avoids the random insertion of transgenes that can otherwise disrupt endogenous gene expression by causing unintended mutations.

A 550-bp region of the mouse genome encoding the miR-31 sequence was amplified from mouse embryonic stem cell DNA and cloned upstream of an enhanced green fluorescent protein (eGFP) expression cassette, and together this was cloned into an integrase-mediated cassette exchange vector. The vector was transfected into RS-PhiC31 embryonic stem cells, which contain a modified *Gt(ROSA*26*)Sor* locus expressing PhiC31 integrase. The PhiC31 integrase system then catalyzes the unidirectional insertion of inverted miR-31/eGFP transgenic constructs between specific attB and attP sequences at the modified *Gt(ROSA*26*)Sor* locus^42^. Recombinant clones were obtained, which harbor an inverted transgene, and were injected into blastocysts. Chimeras were then generated, which were crossed with C57BL/6J strain mice to obtain F1 heterozygotes. Once heterozygotes carrying the inverted miR-31/eGFP transgene were generated, these mice were then crossed with the tamoxifen-inducible fibroblast-specific *Col*1*a*2*-*Cre-ER^T^ mouse strain^43^. Mice carrying both transgenes were treated (intraperitoneally) with 2 mg tamoxifen/30 g mouse (tamoxifen was dissolved in corn oil) for seven consecutive days and compared to wild-type vehicle controls. Induced Cre activity results in inversion of the miR-31/GFP transgene linking it to the CAG promoter, leading to overexpression of miR-31. The *Col*1*a*2*-*Cre-ER^T^-transgenic mouse strain was generated by pro-nuclear injection, which results in random integration of the transgenic allele by homologous recombination at varying copy number^44^. Therefore, *Col*1*a*2*-*Cre-ER^T^ mouse colonies were maintained by crossing hemizygous transgene carriers with wild-type mice to control for *cre* inheritance and transgene dosage. The tamoxifen-inducible fibroblast-specific *Col*1*a*2*-*Cre-ER^T^ strain (Tg(*Col*1*a*2*-cre/ERT*,*-ALPP*)7Cpd/J) was originally obtained from The Jackson Laboratory and maintained on a C57BL/6J background.

For miR-31/Cre studies, age-matched and sex-matched 12-week-old mice were used in this study. Mice were compared to the respective control littermates; females and males were analyzed separately for some experiments (depicted in the figures and figure legends). All animal work was performed in accordance with the local (Baylor College of Medicine and Oxford University) animal care and ethics committee guidance and in accordance with NIH guidelines.

Transgenic approaches for analyzing miRNA function involve driving either short hairpin RNAs (shRNAs) or endogenous primary transcripts (pri-miRNAs) from ubiquitous, tissue-specific or inducible promoters^45^. Such approaches, however, rely upon random insertion of the expression construct into the genome, and at variable copy number, necessitating careful analysis of multiple and individual founder lines. Problems associated with random integration can be avoided by relying on targeted transgenic approaches where transgenic constructs are integrated into safe harbor loci, for example *Gt(ROSA*26*)Sor* locus, known to be permissive for transgenic expression and where integration of a transgene is not associated with any detrimental phenotype. To provide further flexibility, targeted transgenic shRNA systems have been engineered with Cre recombinase switches, which allow tissue-specific control of expression through the introduction of a tissue-specific Cre recombinase transgene.

**Extended Data Fig. 7:**
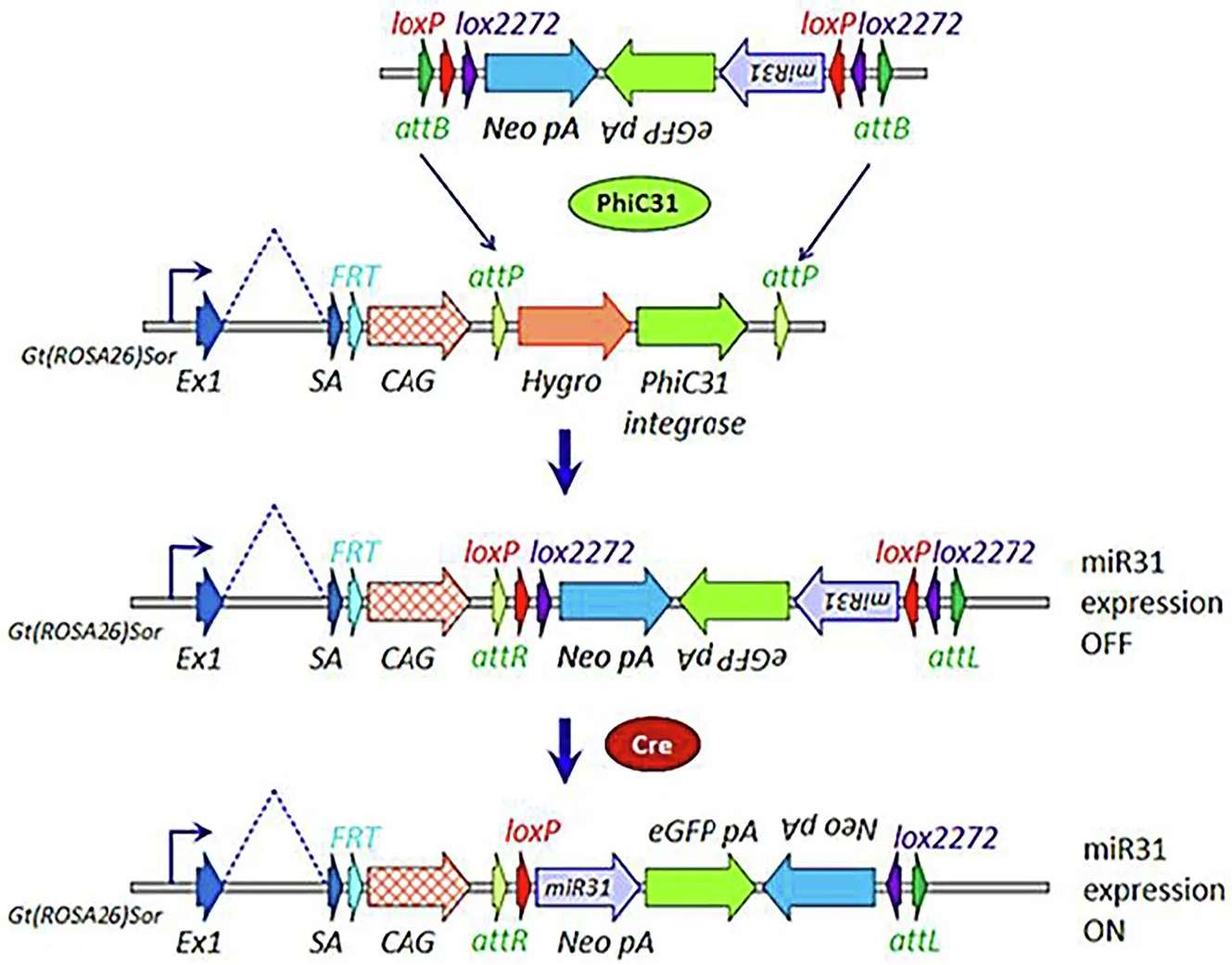
Schematic representation of phi integrase system used in generation of miR-31/Cre mice. *Pre-miR-*31*-Inv* transgenic mice were generated using *PhiC*31 integrase that catalyses unidirectional recombination between specific sequences used for insertion of transgenic constructs into *defined* genomic locations.

With respect to transgenic expression of endogenous miRNAs, a Cre recombinase-dependent inversion strategy has been reported, which allows tissue-specific expression of pri-miRNA^42^. Initially, the miRNA is incorrectly oriented relative to its promoter, but, through the use of heterotypic lox sites, the act of Cre recombinase inverts the pri-mRNA, allowing its correct expression. Here, we combine this Cre inversion strategy with a PhiC31 integrase (Extended Data Fig. 7)-mediated cassette exchange at the *Gt(ROSA*26*)Sor* locus^42^ to allow reliable conditional expression of an endogenous miRNA.

A synthetic linker containing the attB sites, necessary for PhiC31 integrase-mediated targeted integration, flanking an inverted array of loxP and lox2272 sites, necessary for the Cre recombinase-dependent inversion, together with a polylinker of restriction enzyme sites to facilitate the downstream cloning, was generated and cloned into a standard plasmid vector. Into this vector, an open reading frame for the neomycin phosphotransferase gene, with a polyadenylation signal, was cloned immediately downstream of the 5′ loxP and lox2272 sites. An approximately 450-bp region of genomic sequence encompassing the human miR-31 gene was amplified from human genomic DNA using the primers (5′- TCTAGAGGCTGTATTCATCGTTGTCAG-3′ and 5′- GGATCCCAGCCAAGAACATCCCCAAAAGG-3) and cloned downstream of this neomycin selection cassette via XbaI and BamHI restriction sites, which were incorporated into the 5′ ends of the PCR primers. Lastly, an eGFP open reading frame with a rabbit β-globin polyadenylation signal, derived from a standard eGFP expression vector (pCX-eGFP), was cloned downstream of the miR-31 fragment via unique BamHI and HindIII sites.

Five micrograms of the completed vector was electroporated into 1 × 10^6^ RS-PhiC embryonic stem cells, a C57BL/6N JM8F6 derived line^42^, using the Neon transfection system (Life Technologies, NEON18SK) (3 × 1,400 V, 10 ms), and selected in 210 µg ml^−1^ G418 for 7 days. Resistant colonies were isolated, expanded and screened for correct cassette exchange at the *Gt(ROSA*26*)Sor* locus using primers CAG-F (5′-CAGCCATTGCCTTTTATGGT-3′) and ExNeo2 (5′-GTTGTGCCCAGTCATAGCCGAATAG-3′) to verify the 5′ integration event and GFP-NR3 (5′-GCACTAGTTCTAGAGCGATCCCC-3′) and 3HR-R1 (5′-CGGGAGAAATGGATATGAAGTACTGGGC-3′) to verify the 3′ integration event. Recombinant embryonic stem cells were injected into albino C57BL/6J blastocysts, and the resulting chimeras were bred with albino C57BL/6J mice to confirm germline transmission.

### Isolation and culture of primary hACFs

Human ACFs were isolated and cultured from atrial biopsies obtained from patients who underwent cardiac surgery. Tissue biopsies were cut into small (2−3-mm^3^) pieces and repeatedly digested using 4 mg ml^−1^ Collagenase II and trypsin (0.0625%)^13^. Cells were washed twice with sterile PBS and plated onto six-well plates in FBM-3 medium (Lonza, CC-3131) containing 10% FBS and a supplement pack (Lonza, CC-4525) and kept in a humidified atmosphere at 37 °C and 5% CO_2_. The medium was renewed every 2−3 days. At approximately 80−90% confluency, cells were passaged using a standard trypsinization method. For the experiments with TGFβ1 stimulation, we used commercially available donors of human primary atrial fibroblasts (Lonza, CC-2903), which were maintained and cultured in the same medium as outlined above. All experiments were carried out at cell passages 3−4, to minimize effects of culture-induced dedifferentiation, and cells were cultured in serum-free media for 16 hours before intervention and treatment, unless specified otherwise.

### Sources of other human cells

In addition to the human primary cells, we used HEK293 cells (American Type Culture Collection (ATCC), CRL-1573), cultured and maintained according to the manufacturer’s instructions. Cells were authenticated by ATCC. Cultures were routinely monitored for mycoplasma contamination using DAPI staining. The HEK293 cell line was checked against the International Cell Line Authentication Committee (ICLAC) database of commonly misidentified cell lines.

### Transfection of primary hACFs

Human ACFs were transfected with miR-31*-*5p mimic, miR-31*-*5p inhibitor or respective negative controls (NC mimic, NC inhibitor) or with miR-31/CTR-TSB and NC-TSB. Specifically, blockade of miR-31*-*5p−CTR interaction was achieved with 25 nM or 50 nM antisense miRCURY LNA miRNA Power TSB (Qiagen, 339199) G*G*G*C*A*A*G*A*G*G*T*A*A*T*C*T or miRCURY antisense LNA Power TSB negative control A (NC-TSB; Qiagen, 339199) A*C*G*T*C*T*A*T*A*C*G*C*C*C*A; LNA is not shown, as this information is proprietary. Cells were transfected using Lipofectamine RNAiMAX transfection reagent (Invitrogen, 13778030) in antibiotic-deprived FBM-3 medium containing 2% FBS (Lonza).

Inhibition or overexpression of miR-31*-*5p was achieved by miRIDIAN miR-31*-*5p inhibitor (or NC inhibitor) or mimic (or NC mimic), respectively (all from Dharmacon: IH-300507-06, IN-001005-01, C-300507-05 and CN-001000-01), using Lipofectamine RNAiMAX transfection reagent (Invitrogen, 13778030) diluted in Opti-MEM medium (Gibco, 31985062), as described^17^. Efficient knockdown and overexpression were confirmed by real-time qPCR and visually by using the miRIDIAN Dy547-labeled transfection control (for experiments with miR-31*-*5p mimic or inhibitor).

### Isolation and culture of mouse atrial and ventricular cardiomyocytes and fibroblasts

Ventricular and atrial cardiomyocytes and fibroblasts were isolated from miR-31/Cre and Cre/WT mice using enzymatic digestion and mechanical dissociation of the cardiac tissue^1,7,8^. Ventricular and atrial samples were gently cut into small (approximately 1 mm) pieces and washed twice in low-calcium sodium (LCS) (NaCl 120 mM, KCl 1.2 mM, KH_2_PO_4_ 10 mM, MgCl_2_ 1.2 mM, glucose 10 mM, HEPES 10 mM, taurine 20 mM) for 10 minutes in a shaking water bath at 37 °C. After each wash, the mixture was passed through a sterile gauze filter, and the supernatant was discarded. Samples were then incubated twice with LCS containing collagenase (type II, 305 IU ml^−1^; Worthington) and protease (type XIV, ≥3.5 U mg^−1^; Sigma-Aldrich), gassed with 100% O_2_ for 12 minutes as above, and the mixture was passed through a sterile gauze filter; the supernatant was discarded. Thereafter, samples were incubated as above with LCS containing collagenase only, gassed with 100% O_2_ for 15 minutes, and the mixture (containing isolated cell) was then passed through a sterile gauze filter and collected; this process was repeated 4−6 times, with the supernatant collected and pooled with that of prior steps. Cardiomyocytes were collected from the pooled supernatant after a 5-minute centrifugation period at 700 r.p.m. (rotor: Eppendorf, 07/23, 5702/R, A-4-38, maximum 4,400 r.p.m.). Fibroblasts were collected by supernatant centrifugation at 1,300 r.p.m. for 3 minutes and cultured in FBM (Lonza) containing 20% FBS (Lonza).

### Western blot

Immunoblotting in human and mouse atrial homogenates and in hACFs was performed in tissue or cell samples, which were lysed in RIPA buffer or CelLytic M cell lysis reagent; electrophoresis was performed using pre-cast 4−12% NuPAGE gels (Invitrogen, NP0335BOX) and MOPS SDS running buffer (Invitrogen, NP0001) at 150 V. Proteins were transferred onto a 0.2-µm nitrocellulose membrane (Bio-Rad, 1620112) using transfer buffer (Invitrogen, NP0006). Immunodetection of the primary antibodies was performed using anti-IgG HRP-conjugated secondary antibodies (Supplementary Table 7). Membranes were developed using a ChemiDoc System (Bio-Rad) and analyzed with ImageJ software (versions 1.52a and 1.54g) or Image Lab 6.1 (Bio-Rad). For stripping, nitrocellulose membranes were submerged in Restore PLUS Stripping buffer (Thermo Fisher Scientific, 46430)^13^. The target protein and a loading control were always run on the same membrane.

### Colorimetric assays

Quantification of total secreted collagen in the cell culture supernatant was performed using a Sirius Red collagen detection kit (Chondrex, 9049)^1^. In brief, 100 μl of cell culture supernatant or collagen standard was incubated with 500 μl of Sirius Red solution for 20 minutes at room temperature. After centrifugation (10,000 r.p.m., 3 minutes), the collagen-bound Sirius Red pellet was washed, re-centrifuged and dissolved in extraction buffer. Aliquots were transferred to a 96-well plate and absorbance measured at 510−550 nm. Total collagen concentration was calculated from a collagen standard curve and used as a measure of ECM production and fibroblast fibrotic activity. Data are expressed as fold change of their paired NC samples.

The amount of total collagen in mouse atrial lysates was quantified by colorimetric detection of hydroxyproline using a QuickZyme Total Collagen Assay Kit (QuickZyme Biosciences, QZBTOTCOL1, lot 0795) in accordance with the manufacturer’s protocol. In brief, left ventricular or atrial cardiac tissue samples (approximately 15−30 mg) were hydrolyzed in 6 M HCl for 20 hours at 95 °C and centrifuged to remove insoluble material. Hydrolysates were diluted to 4 M HCl, and aliquots were transferred to a 96-well plate together with hydrolyzed collagen standards. After incubation with assay buffer and hydroxyproline detection reagents, samples were incubated for 60 minutes at 60 °C and cooled for 10 minutes, and absorbance was measured at 540 nm. Hydroxyproline concentrations were determined from the standard curve and expressed as fold change of control group.

### Scratch wound migration assay

Human ACF migration was determined using in vitro scratch wound assays. This assay was used to assess fibroblast migratory capacity by quantifying closure of a defined cell-free gap over time. Specifically, scratch wound assay was performed on confluent monolayers of cells (after 24-hour transfection with miR-31*-*5p mimic, miR-31*-*5p inhibitor or respective NCs) using chambers with two-well silicone insert with a defined cell-free gap (ibidi, 80206). In brief, 5 × 10^3^ cells were seeded into each chamber in 70 µl of complete medium (with 10% FBS, as described above). When cells attached and reached approximately 90% confluency, they were synchronized in serum-free medium for 16 hours, which was followed by the chamber insert removal; cells were monitored at different timepoints as depicted in corresponding figures. Changes in the wound area were imaged at the defined timepoints and quantified using ImageJ software^13^.

### Scar-in-a-jar assay

Collagen-1 accumulation by fibroblasts was assessed using a scar-in-a-jar assay. In brief, transfected with miR-31*-*5p mimic, miR-31*-*5p inhibitor or respective controls, cells were plated at 7,000 cells per well overnight in FBM-3 medium containing 10% FBS and supplement pack. The next morning, cells were starved in serum-free medium for approximately 1 hour and cultured for the next 72 hours in 0.5% FBS, 100 µM ascorbic acid (Sigma-Aldrich, A8960) and 37.5 mg ml^−1^ FICOLL 400 (Sigma-Aldrich, F4375) containing medium. After 72 hours, cells were fixed with ice-cold 100% MeOH for 10 minutes, washed three times with PBS and blocked with 3% BSA at room temperature for 1 hour. After an overnight incubation at 4 °C with primary (anti-collagen-1 (Sigma-Aldrich, C4526) and anti-fibronectin (Sigma-Aldrich, F3648)) antibodies and multiple washing steps, the secondary (Life Technologies, A-11001 and A-21245) antibodies and a fluorescent Hoechst solution (nuclear stain) were added for 1.5 hours. After washing steps (with PBS), imaging of cells using ×10 objective and nine fields per well was performed using Operetta software^13^.

### Assessment of cell proliferation

Cell proliferation at a single timepoint was assessed by ELISA using 5-bromo-2′-deoxyuridine (BrDU) DNA-binding probe (Sigma-Aldrich, QIA58) according to the manufacturer’s instructions. In brief, hACFs were plated in a sterile 96-well plate in a medium (FBM-3) containing 10% FBS and supplement pack. Cells were incubated overnight with BrdU (kit component no. JA1595) and fixed the next morning with the Fixative/Denaturing Solution (kit component no. JA1598). Anti-BrdU antibody (kit component no. JA1599) diluted 1:100 in antibody diluent (kit component no. JA1604) was added in each well and incubated for 1 hour at room temperature, followed by three washes with a wash buffer (kit component no. JA1617) before 30-minute incubation of cells with peroxidase goat anti-mouse IgG (kit component no. JA1618) reconstituted with conjugate diluent (kit component no. JA1615), followed by three more washes with wash buffer and ionized water. Cells were then incubated for 15 minutes in the dark at room temperature with the substrate solution and then with the stop solution. Spectrophotometric detection was performed at a wavelength of 450 nm (ref. ^13^).

### Assessment of gene expression with RT−qPCR

Atrial tissue samples were homogenized using an electric homogenizer, Polytron, with an appropriate lysis buffer. Total RNA was extracted with Nucleospin RNA II Kit (Macherey-Nagel, 740955.250) following the manufacturer’s instructions. Complementary DNA (cDNA) was synthesized from 250 ng of RNA with a High-Capacity cDNA Reverse Transcription Kit (Thermo Fisher Scientific, 4368814) according to the manufacturer’s protocol. Total RNA from hACFs was extracted with a *mir*Vana miRNA Isolation Kit (Invitrogen, AM1561). cDNA was synthesized from 100 ng of RNA using QuantiTect Reverse Transcription Kit (Qiagen, 205313). For miR-31*-*5p/3p reverse transcription, the TaqMan MicroRNA Reverse Transcription Kit (Applied Biosystems, 366596) and TaqMan MicroRNA assay reverse transcription primers were used.

Real-time qPCR was performed with TaqMan Fast Advanced Master Mix (Thermo Fisher Scientific, 4444557) or Power SYBR Green PCR Master Mix (Thermo Fisher Scientific, 4367659) accordingly. The gene and miR-31 expression in human samples were assessed using TaqMan gene expression assays or TaqMan MicroRNA assay (Supplementary Tables 8 and 9). To assess gene and miR-31 expression in mouse samples, the TaqMan probes or TaqMan MicroRNA assay and primers (Supplementary Tables 8, 10 and 11) were used.

Each reaction was performed in duplicate (in human samples) or single repeats (in mouse samples) using QuantStudio 7 Flex Real-Time PCR System and ABI 7900HT Detection System (Applied Biosystems) or QuantStudio 5 (Applied Biosystems). Relative quantification was obtained by using the comparative threshold cycle method (${2}^{-\Delta{C}_{t}}$), and expression of the target gene was normalized to GAPDH, RS18 or RLPR7 (for gene targets) or to U6 snRNA (for miR-31 expression) as indicated in each figure; some data are expressed as a fold change of the respective control group (depicted in the figures). For qPCR analyses, transcripts reported as undetermined by the instrument were considered below the limit of detection. These values were recorded as zero for graphical representation and statistical analysis.

### RIP with Ago2 in hACFs

Human ACFs from patients with persAF were transfected with miR-31/CTR-TSB or NC-TSB (25 nM) for 24 hours. The RIP assay was performed using the Magna RIP Kit (Sigma-Aldrich, 17-700). In brief, cells were washed in ice-cold PBS (two times) and lysed in 100 µl of complete RIP lysis buffer. Magnetic beads were conjugated with 5 µg of anti-Ago2 antibody (Abcam, ab32381) and subsequently incubated with the RIP lysates overnight at 4 °C with rotation. Immunoprecipitates were purified and kept overnight at −80 °C to precipitate the RNA and resuspended in RNase-free water. RNAs were subjected to RT−qPCR to measure the *CALCR* mRNA and miR-31 levels. *CALCR* gene expression was normalized to miR-31 and quantified using the comparative threshold cycle method (${2}^{-{\Delta}{C}_{t}}$) as indicated on the graphs and in source data^13^.

### Transcription inhibition with actinomycin D

Human cultured ACFs (see above protocol for cell culture) from patients in SR were transfected with 25 nM miRIDIAN miR-31*-*5p mimic or NC mimic (Supplementary Table 8) for 24 hours and then treated with 5 μg ml^−1^ actinomycin D (Sigma-Aldrich, A9415) for approximately 8.5 hours. Levels of *CALCR* (qPCR) are shown as percentage of the respective mRNA (${2}^{-{\Delta}{C}_{t}}$) at 0 hours, normalizing to *GAPDH* as the housekeeping gene.

### Dual-Glo firefly luciferase reporter assay in HEK293 cells

**Extended Data Fig. 8:**
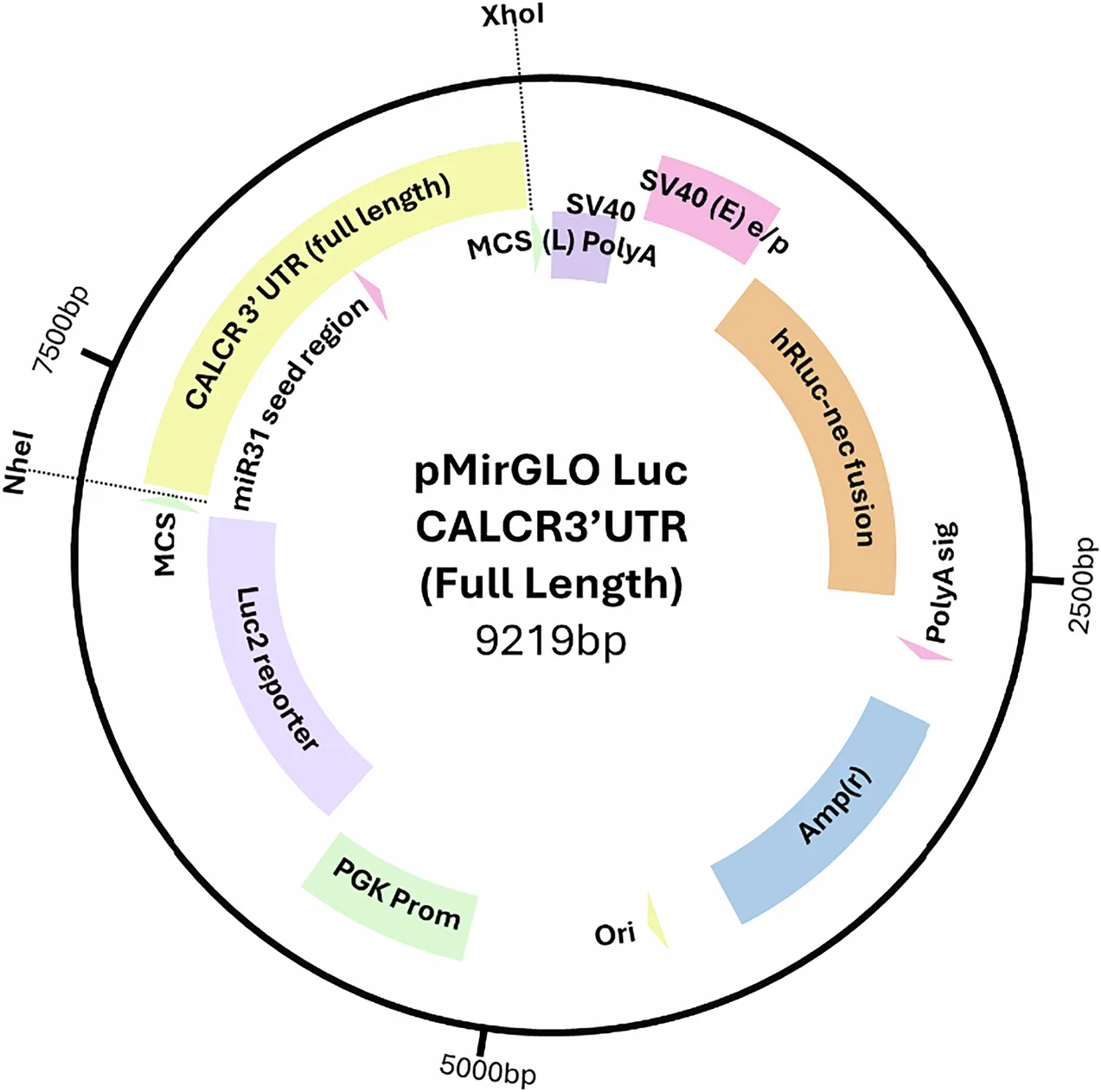
*CALCR* 3′UTR reporter construct used for luciferase assays. Schematic representation of the pmiRGLO dual-luciferase reporter construct containing the full-length human *CALCR* 3′UTR cloned downstream of the firefly luciferase gene. Predicted miRNA recognition elements within the *CALCR* 3′UTR were assessed by co-transfection with miRNA mimics or inhibitors in HEK293 cells.

The reporter construct was generated using the pmirGLO Dual-Luciferase miRNA Target Expression Vector (Promega, E1330). The whole length (1,869 bp) of *CALCR* 3′ UTR containing predicted binding sites for all miRs under investigation was cloned into pmirGLO dual-luciferase vector (Extended Data Fig. 8) using T4DNA ligase cloning with NheI and Xho1 restriction sites, and plasmid DNA was transformed into DH10β Ca^2+^-competent *Escherichia coli* and maxi-prepped using the Qiagen kit manufacturer’s protocol.

HEK293T cells were co-transfected with a reporter construct (250 ng) and a respective miRIDIAN miR-31, *-*34c, *-*30d and *-*24a mimic (a double-stranded sequence of RNA oligonucleotides) and respective inhibitors or mimic/inhibitor negative controls (negative control 1, 10 nM; Dharmacon) using a polyethylenimine transfection reagent (Invitrogen) in antibiotic-free DMEM containing 2% FBS. The firefly/*Renilla* signal was assessed using the Dual-Glo Luciferase Assay System (Promega, E2920) as per the manufacturer’s instructions. The data were expressed as a ratio firefly/*Renilla*.

### Histological assessment of cardiac fibrosis with Masson’s trichrome staining in mice

Staining for collagen was performed using Masson’s trichrome staining. Freshly excised hearts were arrested in diastole with 1 M l^−1^ KCl, fixed in 10% buffered formalin, embedded in paraffin and cut into 6-µm sections for Masson’s trichrome staining using a kit (Sigma-Aldrich, HT15-1KT) in miR-31/Cre and control mice. Sections were imaged at ×20, ×10 and ×4 magnification using Image Pro 7.0 and Zeiss Axio Observer; analysis of the myocardial collagen content was carried out using ImageJ software. All experiments and analyses were performed blinded.

### Transthoracic echocardiography

#### Echo in *Lkb*1-aKD mice (Houston site)

Mice were anesthetized with isoflurane (1.5−2% in 100% oxygen). After hair removal from the chest area using Nair cream, the animals were placed on a heated platform to maintain the body temperature within a range of 36.5−37.5 °C, monitored using a rectal probe. Ultrasound echocardiography was then performed using the VisualSonics Vevo F2 system with a 57-MHz probe (FUJIFILM VisualSonics). ECG readings were continuously observed throughout the procedure. Cardiac short-axis B-mode and M-mode images were obtained to assess ventricular dimensions and systolic function. The short-axis images were analyzed using Vevo LAB software to evaluate systolic function parameters. Additionally, cardiac long-axis B-mode images were captured to visualize the left atrium, and these images were subsequently analyzed to measure the left atrial area. Mice were monitored until they regained consciousness before being returned to their original cages.

#### Echo in miR-31/Cre mice (Oxford site)

Echocardiographic images were acquired with the VisualSonics Vevo 2100 system fitted with an ultra-high-frequency MS-550D MicroScan transducer (22−55 MHz) for miR-31/Cre mice. Parasternal short-axis and long-axis images were obtained at mid-papillary level; mice were then recovered from anesthesia in a heated incubator. Image analysis was blinded between control and treatment groups and performed using Vevo LAB, edition 1.7.1, Imaging System (VisualSonics). Short-axis B-mode images from 3−5 cardiac cycles were averaged and used to estimate left ventricular epicardial end-diastolic area (LV-Epi-EDA) and left ventricular endocardial end-diastolic area (LV-Endo-EDA) and end-systolic area (LV-Endo-ESA). LV-Endo-EDA measurements are used as a surrogate for left ventricular end-diastolic volume (LVEDV) and, hence, dilation. Furthermore, myocardial cross-sectional area (myoCSA) was calculated as a measure of hypertrophy (as it is a surrogate for left ventricular mass) as well as fractional area change (FAC%), as a measure of ejection fraction:

$$\mathrm{MyoCsa}\left({\mathrm{mm}}^{2}\right)=\mathrm{LV}\,\mathrm{Epi}\,\mathrm{EDA}-\mathrm{LV}\,\mathrm{Endo}\,\mathrm{EDA}$$

$$\mathrm{FAC} \% =\frac{\mathrm{LV}\,\mathrm{Endo}\,\mathrm{EDA}-\mathrm{LV}\,\mathrm{Endo}\,\mathrm{ESA}}{\mathrm{LV}\,\mathrm{Endo}\,\mathrm{EDA}}\times 100$$

Echo measurements were similar between two experimenters who independently carried out echocardiography in the same mice (Extended Data Fig. 10).

### In vivo transesophageal burst pacing in miR-31/Cre and WT/Cre mice

AF inducibility in miR-31/Cre and WT/Cre mice was assessed with transesophageal burst pacing. Mice were anesthetized with isoflurane (2%), and an octapolar catheter (World Precision Instruments, CIB’er Mouse EP Catheter) was advanced into the esophagus to capture the atria and deliver electrical stimulation. Intradermal electrodes were implanted to monitor ECG signals throughout. All electrical signals were pre-amplified and recorded using Spike2 software (version 7.09). Each burst was composed of 40 external stimulations at 1.5 times the threshold for atrial capture, starting from a cycle length of 60 ms and decreasing at each successive burst by 2 ms, to a cycle length of 10 ms. Each series of 25 bursts was repeated in triplicate per mouse. An AF episode was defined as a period of rapid irregular rhythm lasting at least 2 seconds. If one or more bursts in the series of burst evoked an arrhythmia/AF episode, AF was considered to be inducible. AF duration was defined as the mean duration of all induced AF episodes. The experimenters were blinded to the genotype throughout the protocol and analysis^17,46^.

### Telemetry studies

Telemetry studies were performed using telemeters (Indus Instruments) implanted subcutaneously in *Lkb*1-aKD mice at 4 weeks of age, and mice were allowed to recover for 2 weeks. Mice were anesthetized with isoflurane (2−3%) during telemeter implantation. For analgesia, we used a combination of 1 mg kg^−1^ SR Buprenorphine and 4 mg kg^−1^ SR Meloxicam for up to 5 days after surgery. Twenty-four-hour ECGs were recorded once a week beginning at week 6. AF was defined as a lack of P-waves and irregularly irregular R−R intervals for more than 10 seconds. AF burden was calculated as time spent in AF versus total time analyzed.

### MRI in mice

**Extended Data Fig. 9:**
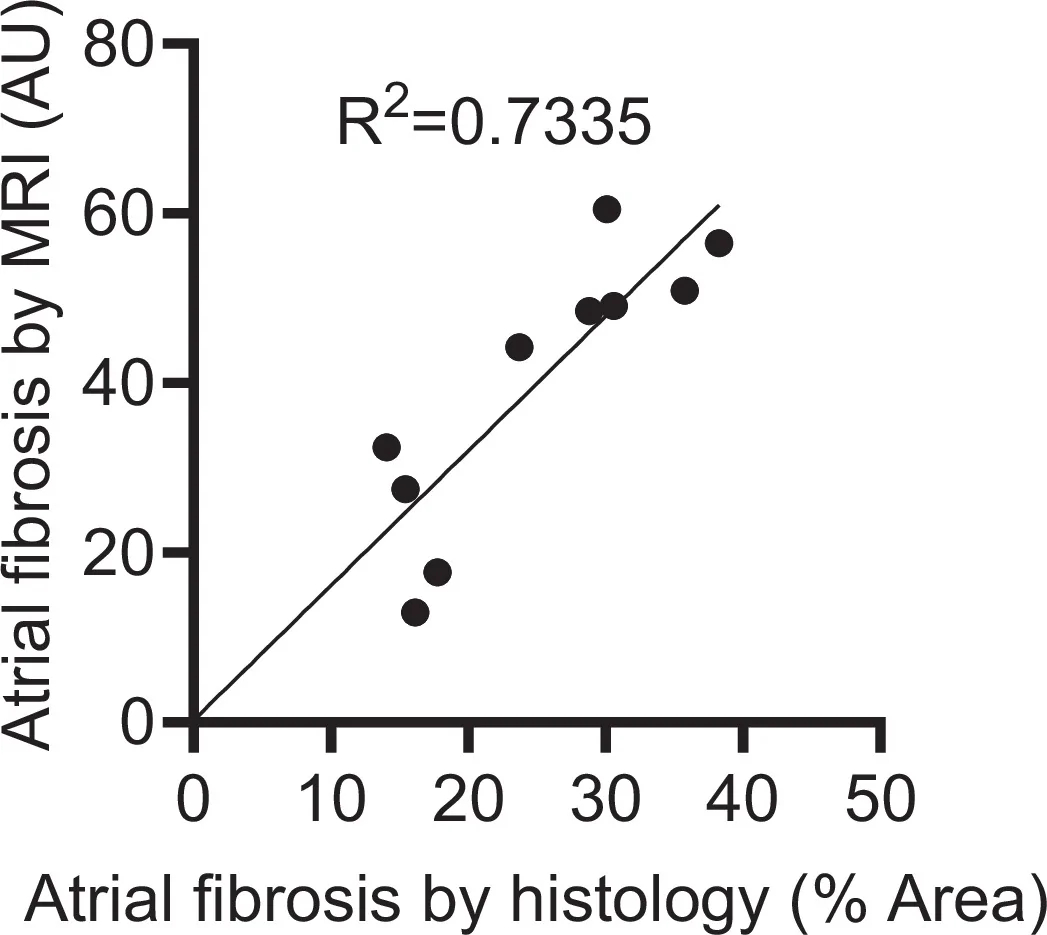
Pearson correlation between atrial fibrosis assessed by MRI and histology (with Masson’s trichrome staining). Data collected from *Lkb*1-aKD mice and plotted using Pearson correlation analysis. Source data

Mice underwent MRI at two timepoints: before miR-31/CTR-TSB or NC-TSB treatment and 3 weeks later. All MRI data were acquired using a Bruker Biospec 9.4 T scanner, 20-cm bore, AV NEO/Paravision 360 (Bruker BioSpin MRI GmbH), at the Small Animal Imaging Facility at Texas Childrenʼs Hospital. Mice were induced with 5% isoflurane mixed with oxygen and then maintained on 0.5−2% isoflurane during imaging. Mice were kept warm utilizing an MRI-compatible heating system by SA Instruments. Respiration rate and temperature were continuously monitored during image acquisition (MRI-compatible INC system; SA Instruments). To assess fibrosis, mice were imaged twice during each imaging session: before and after injection with a T_1_ collagen-binding MRI contrast agent at 4 ml kg^−1^ that detects fibrosis, developed by P. Caravan’s group^47–50^ (Collagen Medical, EP3533). After two scout scans to obtain coronal axis four-chamber and two-chamber view images, high-resolution axial retrospectively gated cine images were obtained using a cine bright-blood intragate fast low-angle shot (FLASH) sequence and imaging parameters: TR/TE = 5.0/1.6 ms; flip angle = 15°; matrix size = 30 × 30 mm^2^; field of view (FOV) = 256 × 256 mm^2^; five slices with a slice thickness of 1.0 mm; and number of averages = 2. Next, retrospectively gated cine black-blood intragate FLASH sequence with the following scanning parameters was acquired: TR/TE = 5.0/1.9 ms; flip angle = 20°; matrix size = 30 × 30 mm^2^; FOV = 128 × 128 mm^2^; five slices with a slice thickness of 1.0 mm; number of averages = 4; tagging resolution of 0.3 mm with 0.6 mm separation; and tag time 16.24 ms. Black-blood images were analyzed using OsiriX software (Pixmeo SARL); atria were segmented, and signal intensities were measured before and after the injection of the contrast agent. The percentage change in signal was recorded and compared between the groups at the different timepoints. On the images, the orange lines span the area where five slices (each 1 mm thick) total were acquired to ensure coverage of the atria. The position of the three green lines is as indicated: the outer two green lines correspond to the edges of the 1-mm slice taken through the coronal view; the middle green line is the center of the 1-mm slice; and the middle green line goes through the center of slice 1 corresponding to the orange line at the bottom. The orange line (top part of the image) corresponds to the center of slice 5. The other slices do not have orange lines, but, because they are 1-mm thick, the green lines show which area of the coronal view they span. The correlation of atrial fibrosis measured by MRI and histological assessment with Masson’s trichrome staining is shown in Extended Data Fig. 9.

### In vivo miR-31/CTR-TSB administration in mice

Blockade of the miR-31−CTR interaction was achieved with antisense miRCURY LNA miRNA Power TSB (Qiagen, 339202) G*G*G*C*A*A*G*A*G*G*T*A*A*T*C*T or NC-TSB (Qiagen, 339202); LNA is not shown, as this information is proprietary (Supplementary Table 12). *Lkb*1-aKD mice were treated with negative- or miR-31/CTR-TSB at either 8 weeks (young cohort) or 20 weeks (older, MRI cohort) of age at doses of 25 mg kg^−1^. TSB was injected per mouse intraperitoneally—two (younger mice) or three (older mice) injections per week for 3 weeks. A 5 µg µl^−1^ solution was prepared in sterile saline, and the following formula was used to calculate volume to be injected: volume to be injected (µl) = 20 ×mouse weight (g) / 5.

### A sandwich hybridization assay of LNA-TSB

A sandwich hybridization assay was performed using a biotinylated LNA capture probe complementary to half of the LNA-miR-31/CTR-TSB to capture the LNA-TSB on streptavidin-coated magnetic beads. In brief, total RNA was extracted from homogenized tissues using the phenol:chloroform:isoamyl alcohol extraction method. A 2-pM biotinylated probe (Qiagen, CALCRMIRPROBE1) was added to the plate. After 30-minute incubation at room temperature, the plate was washed, and 100 µl of diluted extract was added to the plate, followed by a 30-minute incubation at room temperature and washes. Next, a 2-pM digoxigenin (DIG)-coupled complementary probe (Qiagen, CALCRMIRPROBE2) was added to the plate, followed by a 30-minute incubation at room temperature and washes. The captured LNA-TSBs were detected using the HRP-conjugated anti-DIG antibody (Roche, 11633716001) and detected at 450 nm using a standard plate reader.

### RNA-seq: sample processing and analysis

Total RNA was extracted from hACF cultures using a *mir*Vana miRNA Isolation Kit (Thermo Fisher Scientific, AM1560), as above. For SR-ACF miR-31 mimic dataset, RNA samples were treated with RNase-Free DNase Set (Qiagen, 79254), whereby 5 µl of Buffer RDD was added to each sample, followed by 1.25 µl of DNase I stock solution (2.7 Kunitz units per μl). RNA cleanup was then performed by precipitating RNA with 1.25 volumes of 100% ethanol, which was then applied to fresh *mir*Vana miRNA Isolation Kit columns (Thermo Fisher Scientific, AM1560) that were then washed as above before eluting clean RNA. Sample replicates with 260/230 and 260/280 NanoDrop spectrophotometric absorbance ratios of approximately 2 were selected. Then, 750 ng of RNA per sample was made up in 30 µl of nuclease-free water, which was then used for RNA-seq transcriptional profiling, which was performed as a service at the genomic core facility of the Wellcome Centre for Human Genetics (Oxford). Total RNA quantity and integrity were assessed there using the Quant-IT RiboGreen RNA Assay Kit (Thermo Fisher Scientific, R11490) and a TapeStation system (Agilent Technologies). Samples were then normalized to 100 ng. RNA was enriched for protein-encoding mRNA transcripts by using polyadenosine oligonucleotides conjugated to magnetic beads with the NEBNext Magnetic Isolation Module (New England Biolabs, E7490). Generation of double-stranded cDNA and library construction were performed using the NEBNext Ultra II Directional RNA Library Prep Kit (New England Biolabs, E7760L) with dual-indexing adapters and barcode tags, based on a previous report^51^. The concentrations used to generate the multiplex pool were determined by PicoGreen. The final size distribution of the pool was determined using a TapeStation (Agilent Technologies), and quantification was determined by Qubit (Thermo Fisher Scientific, Invitrogen) before sequencing on an Illumina NovaSeq 6000 as 150-bp paired end.

For the persAF ACF miR-31-5p inhibitor dataset, RNA samples were quantified using a Qubit 4.0 Fluorometer (Life Technologies), and RNA integrity was checked with the RNA Kit on an Agilent 5300 Fragment Analyzer (Agilent Technologies).

RNA sequencing libraries were prepared using the NEBNext Ultra II RNA Library Prep Kit for Illumina following the manufacturer’s instructions (New England Biolabs). In brief, mRNAs were first enriched with oligo(dT) beads. Enriched mRNAs were fragmented for 15 minutes at 94 °C. First-strand and second-strand cDNA was subsequently synthesized. cDNA fragments were end repaired and adenylated at 3′ ends, and universal adapters were ligated to cDNA fragments, followed by index addition and library enrichment by limited-cycle PCR. Sequencing libraries were validated using the NGS Kit on the Agilent 5300 Fragment Analyzer (Agilent Technologies) and quantified by using a Qubit 4.0 Fluorometer (Invitrogen).

The sequencing libraries were multiplexed and loaded on the flowcell on the Illumina NovaSeq instrument according to the manufacturer’s instructions. The samples were sequenced using a 2 × 150 paired-end configuration version 1.5. Image analysis and base calling were conducted by NovaSeq Control Software version 1.7 on the NovaSeq instrument. Raw sequence data (.bcl files) generated from Illumina NovaSeq were converted into fastq files and demultiplexed using Illumina bcl2fastq program version 2.20. One mismatch was allowed for index sequence identification. Paired-end 150-bp reads were trimmed with Trim Galore (version 0.6.7) (https://www.bioinformatics.babraham.ac.uk/projects/trim_galore/) and checked for quality using FastQC (version 0.12.1) (https://www.bioinformatics.babraham.ac.uk/projects/fastqc/). Reads were then aligned to the human genome (GRCh38.p14) using HISAT2 (ref. ^52^) (version 2.2.1) with an average of 95% of reads aligned uniquely using default parameters. Gene counts were determined by the number of uniquely aligned, unambiguous reads using Subread featureCounts^53^ (version 1.6.4) and annotated with GRCH38.111. Approximately 78% of reads in each sample were successfully assigned to genes. Raw counts were loaded into R (version 4.4.0), and pre-filtering was performed to keep only genes with a count of at least 10 in a minimal number of samples. Differential expression analysis was performed using DESeq2. The resulting count values were used for library size normalization, log_2_ transformation and downstream analysis. Statistically significant DEGs were determined by a Benjamini−Hochberg-adjusted *P* < 0.05 and log_2_ fold change > 1 or <−1 (for SR mimic dataset) and log_2_ fold change > 0.585 or <−0.585 (for AF inhibitor dataset) Functional enrichment analysis of significant DEGs was performed using clusterProfiler (version 4.12.0)^54^ in R. Additional plots were generated using in-house R scripts (available upon reasonable request). RNA-seq data have been deposited in the Gene Expression Omnibus (GEO) database under accession number GSE294333.

Plasmids and other materials generated during this study are available from the corresponding authors upon reasonable request.

### Statistics and reproducibility

The data distribution was tested by Kolmogorov−Smirnov or Shapiro−Wilk tests. The unpaired or paired Student’s *t*-test was used in two-group comparisons of the normally distributed data. Multiple groups of normally distributed data of similar variance were compared by one-way or two-way ordinary or repeated-measures ANOVA with Holm−Sidak or Sidak’s correction. When the normality assumption was not met, the data were analyzed by Mann−Whitney or Wilcoxon test (for independent or paired comparisons between two groups, respectively) or Kruskal−Wallis test with Dunn’s correction (for multiple group comparisons). Categorical variables were compared by Fisher’s exact test. All statistical analysis was performed using GraphPad Prism version 7, version 6.04, version 9.0 or version 10 software. A value of *P* < 0.05 was considered statistically significant. Pearson’s correlation test was used to test correlation between two normally distributed variables.

All experiments were performed using biologically independent samples unless otherwise stated. For studies using primary human atrial fibroblasts, biologically independent samples corresponded to cells isolated from different donors. For mouse studies, biologically independent samples corresponded to individual animals. Technical replicates were not treated as independent biological replicates for statistical analyses. Representative images, histological sections and immunoblots shown in the figures were selected from biologically independent samples included in the corresponding quantitative analyses and were representative of the overall findings. Exact *n* values are indicated in the figures and figure legends. Key findings were independently reproduced across multiple biological systems (humans and mice) and complementary experimental approaches. Independent replication confirmed the principal conclusions of the study across human primary cells and mouse models.

### Reporting summary

Further information on research design is available in the Nature Portfolio Reporting Summary linked to this article.

## Supplementary information

Reporting Summary

Supplementary Tables Tables 1−12, including clinical characteristics, echocardiographic analyses, antibodies, oligonucleotides, qPCR assays and antisense miRCURY LNA sequences.

## Source data

Source Data Fig. 1. Statistical source data underlying all graphs in Fig. 1.

Source Data Fig. 2. Statistical source data underlying all graphs in Fig. 2.

Source Data Fig. 3. Statistical source data underlying all graphs in Fig. 3.

Source Data Fig. 4. Statistical source data underlying all graphs in Fig. 4.

Source Data Fig. 5. Statistical source data underlying all graphs in Fig. 5.

Source Data Figs. 1−5 and Extended Data Figs. 2−5. Uncropped western blots and PCR gels corresponding to Figs. 1, 3, 4 and 5 and Extended Data Figs. 2−5.

Source Data Extended Data Fig. 1. Statistical source data.

Source Data Extended Data Fig. 3. Statistical source data.

Source Data Extended Data Fig. 4. Statistical source data.

Source Data Extended Data Fig. 5. Statistical source data.

Source Data Extended Data Fig. 6. Statistical source data.

Source Data Extended Data Fig. 9. Statistical source data.

**Extended Data Fig. 10:**
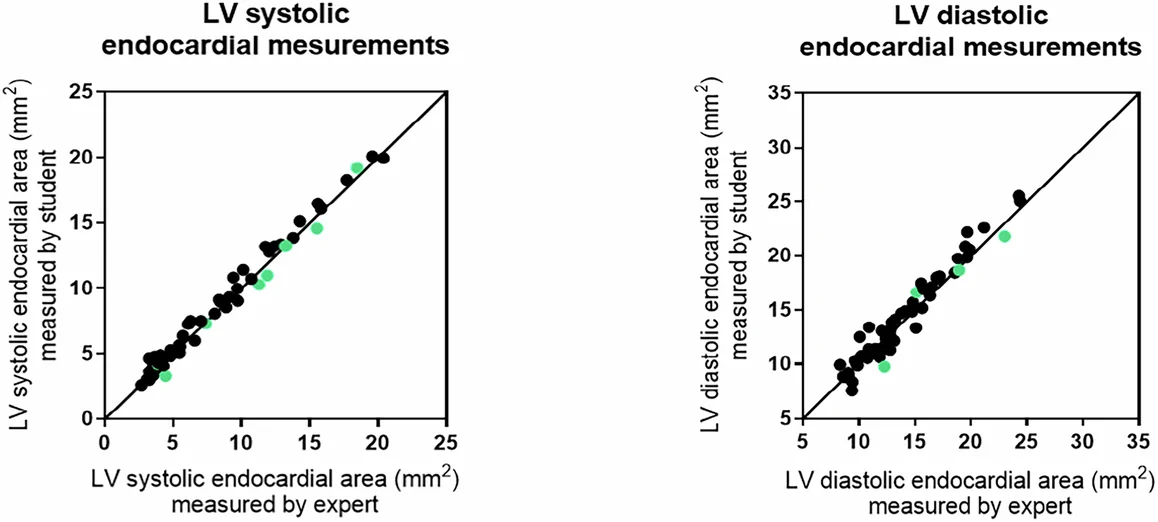
Pearson’s correlation for inter-experimenter reproducibility of selected echo parameters. Data points obtained by two independent experimenters shown by black and green dots. Source data

Source Data Extended Data Fig. 10. Statistical source data.

## Data Availability

All data generated or analyzed during this study are included in the article, source data files, extended data items and supplementary tables associated with the paper. RNA-seq data have been deposited in the GEO database under accession number GSE294333. Additional information supporting the findings of this study is available from the corresponding authors upon reasonable request.

## Code availability

No custom code was generated or used in this study.

## References

[1] Ball, J., Carrington, M. J., McMurray, J. J. & Stewart, S. Atrial fibrillation: profile and burden of an evolving epidemic in the 21st century. *Int. J. Cardiol.* **167**, 1807–1824 (2013). 10.1016/j.ijcard.2012.12.093 23380698

[2] Chugh, S. S. et al. Worldwide epidemiology of atrial fibrillation: a Global Burden of Disease 2010 Study. *Circulation* **129**, 837–847 (2014). 10.1161/CIRCULATIONAHA.113.005119 PMC4151302 24345399

[3] Wijffels, M. C., Kirchhof, C. J., Dorland, R. & Allessie, M. A. Atrial fibrillation begets atrial fibrillation. A study in awake chronically instrumented goats. *Circulation* **92**, 1954–1968 (1995). 10.1161/01.cir.92.7.1954 7671380

[4] Nattel, S. New ideas about atrial fibrillation 50 years on. *Nature* **415**, 219–226 (2002). 10.1038/415219a 11805846

[5] Clarnette, J. A. et al. Outcomes of persistent and long-standing persistent atrial fibrillation ablation: a systematic review and meta-analysis. *Europace* **20**, f366–f376 (2018). 10.1093/europace/eux297 29267853

[6] Chelu, M. G. et al. Atrial fibrosis by late gadolinium enhancement magnetic resonance imaging and catheter ablation of atrial fibrillation: 5-year follow-up data. *J. Am. Heart Assoc.* **7**, e006313 (2018). 10.1161/JAHA.117.006313 PMC6405558 30511895

[7] Heijman, J., Guichard, J. B., Dobrev, D. & Nattel, S. Translational challenges in atrial fibrillation. *Circ. Res.* **122**, 752–773 (2018). 10.1161/CIRCRESAHA.117.311081 29496798

[8] Nattel, S. Molecular and cellular mechanisms of atrial fibrosis in atrial fibrillation. *JACC Clin. Electrophysiol.* **3**, 425–435 (2017). 10.1016/j.jacep.2017.03.002 29759598

[9] Marrouche, N. F. et al. Association of atrial tissue fibrosis identified by delayed enhancement MRI and atrial fibrillation catheter ablation: the DECAAF study. *JAMA* **311**, 498–506 (2014). 10.1001/jama.2014.3 24496537

[10] Zhou, Q. et al. Circulating microRNA-21 correlates with left atrial low-voltage areas and is associated with procedure outcome in patients undergoing atrial fibrillation ablation. *Circ. Arrhythm. Electrophysiol.* **11**, e006242 (2018). 10.1161/CIRCEP.118.006242 PMC7661044 29848477

[11] Li, Y. et al. Increased expression of connective tissue growth factor and transforming growth factor-beta-1 in atrial myocardium of patients with chronic atrial fibrillation. *Cardiology* **124**, 233–240 (2013). 10.1159/000347126 23571482

[12] Rudolph, V. et al. Myeloperoxidase acts as a profibrotic mediator of atrial fibrillation. *Nat. Med.* **16**, 470–474 (2010). 10.1038/nm.2124 PMC2880896 20305660

[13] Moreira, L. M. et al. Paracrine signalling by cardiac calcitonin controls atrial fibrogenesis and arrhythmia. *Nature* **587**, 460–465 (2020). 10.1038/s41586-020-2890-8 33149301

[14] Zaidi, M., Inzerillo, A. M., Moonga, B. S., Bevis, P. J. R. & Huang, C. L. H. Forty years of calcitonin: where are we now? A tribute to the work of Iain Macintyre, FRS. *Bone* **30**, 655–663 (2002). 10.1016/s8756-3282(02)00688-9 11996901

[15] O’Brien, J., Hayder, H., Zayed, Y. & Peng, C. Overview of microRNA biogenesis, mechanisms of actions, and circulation. *Front. Endocrinol. (Lausanne)* **9**, 402 (2018). 10.3389/fendo.2018.00402 PMC6085463 30123182

[16] Briasoulis, A. et al. MicroRNAs in atrial fibrillation. *Curr. Med. Chem.* **26**, 855–863 (2019). 10.2174/0929867324666170920151024 28933293

[17] Reilly, S. N. et al. Up-regulation of miR-31 in human atrial fibrillation begets the arrhythmia by depleting dystrophin and neuronal nitric oxide synthase. *Sci. Transl. Med.* **8**, 340ra74 (2016). 10.1126/scitranslmed.aac4296 PMC4993239 27225184

[18] Reilly, S. et al. Evaluation of the role of miR-31-dependent reduction in dystrophin and nNOS on atrial-fibrillation-induced electrical remodelling in man. *Lancet* **385**, S82 (2015). 10.1016/S0140-6736(15)60397-X 26312904

[19] Soo, Y. et al. Age-specific trends of atrial fibrillation-related ischaemic stroke and transient ischaemic attack, anticoagulant use and risk factor profile in Chinese population: a 15-year study. *J. Neurol. Neurosurg. Psychiatry* **88**, 744–748 (2017). 10.1136/jnnp-2017-315735 28550068

[20] Xu, R. et al. MicroRNA-31a-5p from aging BMSCs links bone formation and resorption in the aged bone marrow microenvironment. *Aging Cell* **17**, e12794 (2018). 10.1111/acel.12794 PMC6052401 29896785

[21] Capri, M. et al. Identification of miR-31-5p, miR-141-3p, miR-200c-3p, and GLT1 as human liver aging markers sensitive to donor-recipient age-mismatch in transplants. *Aging Cell* **16**, 262–272 (2017). 10.1111/acel.12549 PMC5334540 27995756

[22] Cho, J. H., Dimri, M. & Dimri, G. P. MicroRNA-31 is a transcriptional target of histone deacetylase inhibitors and a regulator of cellular senescence. *J. Biol. Chem.* **290**, 10555–10567 (2015). 10.1074/jbc.M114.624361 PMC4400362 25737447

[23] Luan, L., Shi, J., Yu, Z. & Andl, T. The major miR-31 target genes STK40 and LATS2 and their implications in the regulation of keratinocyte growth and hair differentiation. *Exp. Dermatol.* **26**, 497–504 (2017). 10.1111/exd.13355 28419554

[24] Wang, N., Li, Y. & Zhou, J. miR-31 functions as an oncomir which promotes epithelial-mesenchymal transition via regulating BAP1 in cervical cancer. *Biomed. Res. Int.* **2017**, 6361420 (2017). 10.1155/2017/6361420 PMC5660773 29159179

[25] Villalobos, E. et al. Fibroblast primary cilia are required for cardiac fibrosis. *Circulation* **139**, 2342–2357 (2019). 10.1161/CIRCULATIONAHA.117.028752 PMC6517085 30818997

[26] Straarup, E. M. et al. Short locked nucleic acid antisense oligonucleotides potently reduce apolipoprotein B mRNA and serum cholesterol in mice and non-human primates. *Nucleic Acids Res.* **38**, 7100–7111 (2010). 10.1093/nar/gkq457 PMC2978335 20615897

[27] Hulsurkar, M. M. et al. Atrial-specific LKB1 knockdown represents a novel mouse model of atrial cardiomyopathy with spontaneous atrial fibrillation. *Circulation* **144**, 909–912 (2021). 10.1161/CIRCULATIONAHA.121.055373 PMC8442761 34516304

[28] Kuppahally, S. S. et al. Left atrial strain and strain rate in patients with paroxysmal and persistent atrial fibrillation: relationship to left atrial structural remodeling detected by delayed-enhancement MRI. *Circ. Cardiovasc. Imaging* **3**, 231–239 (2010). 10.1161/CIRCIMAGING.109.865683 20133512

[29] Yoo, S. P. et al. Gadolinium-functionalized peptide amphiphile micelles for multimodal imaging of atherosclerotic lesions. *ACS Omega* **1**, 996–1003 (2016). 10.1021/acsomega.6b00210 PMC5131325 27917409

[30] Hildebrandt-Eriksen, E. S. et al. A locked nucleic acid oligonucleotide targeting microRNA 122 is well-tolerated in cynomolgus monkeys. *Nucleic Acid Ther.* **22**, 152–161 (2012). 10.1089/nat.2011.0332 22545703

[31] Lai, Y. J. et al. miR-181b targets semaphorin 3A to mediate TGF-β-induced endothelial-mesenchymal transition related to atrial fibrillation. *J. Clin. Invest.* **132**, e142548 (2022). 10.1172/JCI142548 PMC9246393 35775491

[32] Calebiro, D. & Godbole, A. Internalization of G-protein-coupled receptors: Implication in receptor function, physiology and diseases. *Best Pract. Res. Clin. Endocrinol. Metab.* **32**, 83–91 (2018). 10.1016/j.beem.2018.01.004 29678288

[33] Martinez, E. C. et al. MicroRNA-31 promotes adverse cardiac remodeling and dysfunction in ischemic heart disease. *J. Mol. Cell. Cardiol.* **112**, 27–39 (2017). 10.1016/j.yjmcc.2017.08.013 28865712

[34] Huang, R., Chen, X., Long, Y. & Chen, R. MiR-31 promotes Th22 differentiation through targeting Bach2 in coronary heart disease. *Biosci. Rep.* **39**, BSR20190986 (2019). 10.1042/BSR20190986 PMC6753318 31501353

[35] Pondel, M. D., Jagger, C., Hebden, C., Partington, G. & Mould, R. Transcriptional regulation of the calcitonin receptor gene. *Biochem. Soc. Trans.* **30**, 423–427 (2002). 10.1042/bst0300423 12196107

[36] Wu, S. et al. Multiple microRNAs modulate p21Cip1/Waf1 expression by directly targeting its 3’ untranslated region. *Oncogene* **29**, 2302–2308 (2010). 10.1038/onc.2010.34 20190813

[37] Cha, T. J. et al. Dissociation between ionic remodeling and ability to sustain atrial fibrillation during recovery from experimental congestive heart failure. *Circulation* **109**, 412–418 (2004). 10.1161/01.CIR.0000109501.47603.0C 14718405

[38] Saljic, A., Heijman, J. & Dobrev, D. Emerging antiarrhythmic drugs for atrial fibrillation. *Int. J. Mol. Sci.* **23**, 4096 (2022). 10.3390/ijms23084096 PMC9029767 35456912

[39] Maleitzke, T. et al. The calcitonin receptor protects against bone loss and excessive inflammation in collagen antibody-induced arthritis. *iScience* **25**, 103689 (2021). 10.1016/j.isci.2021.103689 PMC8753130 35036874

[40] Gooi, J. H. et al. Decline in calcitonin receptor expression in osteocytes with age. *J. Endocrinol.* **221**, 181–191 (2014). 10.1530/JOE-13-0524 24516262

[41] Weilner, S. et al. Secreted microvesicular miR-31 inhibits osteogenic differentiation of mesenchymal stem cells. *Aging Cell* **15**, 744–754 (2016). 10.1111/acel.12484 PMC4933673 27146333

[42] Dolatshad, H. et al. A versatile transgenic allele for mouse overexpression studies. *Mamm. Genome* **26**, 598–608 (2015). 10.1007/s00335-015-9602-y PMC4653235 26369329

[43] Zheng, B., Zhang, Z., Black, C. M., de Crombrugghe, B. & Denton, C. P. Ligand-dependent genetic recombination in fibroblasts: a potentially powerful technique for investigating gene function in fibrosis. *Am. J. Pathol.* **160**, 1609–1617 10.1016/S0002-9440(10)61108-X (2002). PMC1850857 12000713

[44] Palmiter, R. & Brinster, R. Germ-line transformation of mice. *Ann. Rev. Genet.* **20**, 465–499 (1986). 10.1146/annurev.ge.20.120186.002341 PMC5553637 3545063

[45] Kunath, T. et al. Transgenic RNA interference in ES cell-derived embryos recapitulates a genetic null phenotype. *Nat. Biotechnol.* **21**, 559–561 (2003). 10.1038/nbt813 12679785

[46] Verheule, S. et al. Increased vulnerability to atrial fibrillation in transgenic mice with selective atrial fibrosis caused by overexpression of TGF-β1. *Circ. Res.* **94**, 1458–1465 (2004). 10.1161/01.RES.0000129579.59664.9d PMC2129102 15117823

[47] Moon, B. F. et al. Simultaneous positron emission tomography and molecular magnetic resonance imaging of cardiopulmonary fibrosis in a mouse model of left ventricular dysfunction. *J. Am. Heart Assoc.* **13**, e034363 (2024). 10.1161/JAHA.124.034363 PMC11292745 38979786

[48] Murphy, A. P. et al. Noninvasive quantification of fibrosis in skeletal and cardiac muscle in mdx mice using EP3533 enhanced magnetic resonance imaging. *Magn. Reson. Med.* **81**, 2728–2735 (2019). 10.1002/mrm.27578 30394578

[49] Spuentrup, E. et al. Molecular magnetic resonance imaging of myocardial perfusion with EP-3600, a collagen-specific contrast agent: initial feasibility study in a swine model. *Circulation* **119**, 1768–1775 (2009). 10.1161/CIRCULATIONAHA.108.826388 19307474

[50] Desogere, P., Montesi, S. B. & Caravan, P. Molecular probes for imaging fibrosis and fibrogenesis. *Chemistry* **25**, 1128–1141 (2019). 10.1002/chem.201801578 PMC6542638 30014529

[51] Lamble, S. et al. Improved workflows for high throughput library preparation using the transposome-based Nextera system. *BMC Biotechnol.* **13**, 104 10.1186/1472-6750-13-104 (2013). PMC4222894 24256843

[52] Kim, D., Paggi, J. M., Park, C., Bennett, C. & Salzberg, S. L. Graph-based genome alignment and genotyping with HISAT2 and HISAT-genotype. *Nat. Biotechnol.* **37**, 907–915 10.1038/s41587-019-0201-4 (2019). PMC7605509 31375807

[53] Liao, Y., Smyth, G. K. & Shi, W. The Subread aligner: fast, accurate and scalable read mapping by seed-and-vote. *Nucleic Acids Res.* **41**, e108 (2013). 10.1093/nar/gkt214 PMC3664803 23558742

[54] Yu, G., Wang, L. G., Han, Y. & He, Q. Y. clusterProfiler: an R package for comparing biological themes among gene clusters. *OMICS* **16**, 284–287 (2012). 10.1089/omi.2011.0118 PMC3339379 22455463

