# Supplementary material for: Selective blockade of microRNA-31-5p−calcitonin receptor interaction reverses established atrial fibrosis and atrial arrhythmia burden
Source: Nat Cardiovasc Res. 2026 Oct 30;5(11):1231–49. doi: 10.1038/s44161-026-00897-1 (PMC13759305; doi:10.1038/s44161-026-00897-1)
Supplement: Supplementary file 1 [file 44161_2026_897_MOESM1_ESM.pdf]

nature portfolio

Corresponding author(s): Svetlana Reilly, Xander H T Wehrens

Last updated by author(s): Sep 15, 2026

Reporting Summary

Nature Portfolio wishes to improve the reproducibility of the work that we publish. This form provides structure for consistency and transparency in reporting. For further information on Nature Portfolio policies, see our Editorial Policies and the Editorial Policy Checklist.

Statistics

For all statistical analyses, confirm that the following items are present in the figure legend, table legend, main text, or Methods section.

n/a	Confirmed	
☐	☒	The exact sample size (n) for each experimental group/condition, given as a discrete number and unit of measurement
☐	☒	A statement on whether measurements were taken from distinct samples or whether the same sample was measured repeatedly
☐	☒	The statistical test(s) used AND whether they are one- or two-sided<br>*Only common tests should be described solely by name; describe more complex techniques in the Methods section.*
☐	☒	A description of all covariates tested
☐	☒	A description of any assumptions or corrections, such as tests of normality and adjustment for multiple comparisons
☐	☒	A full description of the statistical parameters including central tendency (e.g. means) or other basic estimates (e.g. regression coefficient) AND variation (e.g. standard deviation) or associated estimates of uncertainty (e.g. confidence intervals)
☐	☒	For null hypothesis testing, the test statistic (e.g. F, t, r) with confidence intervals, effect sizes, degrees of freedom and P value noted<br>*Give P values as exact values whenever suitable.*
☒	☐	For Bayesian analysis, information on the choice of priors and Markov chain Monte Carlo settings
☒	☐	For hierarchical and complex designs, identification of the appropriate level for tests and full reporting of outcomes
☐	☒	Estimates of effect sizes (e.g. Cohen's d, Pearson's r), indicating how they were calculated

Our web collection on statistics for biologists contains articles on many of the points above.

Software and code

Policy information about availability of computer code

Data collection	Commercial software was used for data acquisition. Electrophysiological signals were recorded using Spike2 software (version 7.09). Echocardiographic images were acquired using VisualSonics Vevo systems and associated Vevo Lab software. MRI data were acquired using Bruker Biospec systems with Paravision software. No custom code was used for data collection.
Data analysis	Data analysis was performed using commercially available software, including GraphPad Prism (versions 6.04, 7, 9.0, and 10) for statistical analysis, ImageJ (version 1.52a, 1.54g) and Image Lab (version 6.1, Bio-Rad) for image and immunoblot quantification, OsiriX for MRI image analysis, and Vevo Lab software for echocardiographic analysis. RNA-sequencing data were analysed using established bioinformatics tools, including HISAT2, Subread featureCounts, DESeq2, and clusterProfiler implemented in R (version 4.4.0), with additional in-house R scripts used for visualization. No custom algorithms central to the study were developed.

For manuscripts utilizing custom algorithms or software that are central to the research but not yet described in published literature, software must be made available to editors and reviewers. We strongly encourage code deposition in a community repository (e.g. GitHub). See the Nature Portfolio guidelines for submitting code & software for further information.

Data

Policy information about availability of data

All manuscripts must include a data availability statement. This statement should provide the following information, where applicable:

- Accession codes, unique identifiers, or web links for publicly available datasets
- A description of any restrictions on data availability
- For clinical datasets or third party data, please ensure that the statement adheres to our policy

All data generated or analysed during this study are included in this published article. RNAseq data have been deposited in NCBI's Gene Expression Omnibus and are accessible through GEO Series accession number GSE294333 (https://www.ncbi.nlm.nih.gov/geo/query/acc.cgi?acc=GSE294333). Source data for all graphically expressed figures are provided with the paper.

Research involving human participants, their data, or biological material

Policy information about studies with human participants or human data. See also policy information about sex, gender (identity/presentation), and sexual orientation and race, ethnicity and racism.

Reporting on sex and gender	Sex was recorded as a biological attribute based on medical records. The study included 145 patients (103 men, 42 women). Gender identity data were not collected, and therefore gender-based analyses were not performed.
Reporting on race, ethnicity, or other socially relevant groupings	Data on race, ethnicity, or other socially relevant social categorizations were not collected in this study and therefore were not included in the analyses.
Population characteristics	The study population consisted of 145 patients, including 70 with sinus rhythm and 75 with persistent atrial fibrillation. Mean age was 68 ± 9 years in the sinus rhythm group and 71 ± 9 years in the persistent atrial fibrillation group. Overall, the cohort comprised 103 males (71%) and 42 females (29%).Clinical characteristics included prior cardiac surgery (CABG, AVR, MVR), cardiovascular risk factors (hypertension, diabetes mellitus, heart failure), prior myocardial infarction, and pulmonary disease (COPD/asthma). Medication use at baseline included anticoagulants, antiplatelets, β-blockers, statins, calcium-channel blockers, ACE inhibitors/ARBs, and diuretics.
Recruitment	Patients undergoing cardiac surgery (coronary artery bypass grafting or valve repair/replacement) at the John Radcliffe Hospital, Oxford, were recruited for this study. All participants provided written informed consent. The study cohort comprised 145 patients. Participants were recruited from a single tertiary referral cardiac surgery centre. The cohort therefore may not be representative of all patients with atrial fibrillation and may be enriched for individuals with structural heart disease requiring surgical intervention.
Ethics oversight	The study was approved by the South Central–Berkshire B Research Ethics Committee (REF #18/SC/0404), and all participants provided written informed consent.

Note that full information on the approval of the study protocol must also be provided in the manuscript.

Field-specific reporting

Please select the one below that is the best fit for your research. If you are not sure, read the appropriate sections before making your selection.

☒ Life sciences ☐ Behavioural & social sciences ☐ Ecological, evolutionary & environmental sciences

For a reference copy of the document with all sections, see nature.com/documents/nr-reporting-summary-flat.pdf

Life sciences study design

All studies must disclose on these points even when the disclosure is negative.

Sample size	A total of 145 human patients were included. For animal studies, a total of 175 mice were used across all experiments (66 Lkb1-aKD mice and 109 miR-31/Cre mice). Sample sizes were determined based on prior experience with the experimental models and expected effect sizes from previous studies using the same models. No formal statistical sample-size calculation was performed. Animal numbers were selected to minimise animal use while maintaining adequate power to detect biologically meaningful differences between groups. Individual experiments were performed using available subsets of this cohort, as indicated by the corresponding n values in the figures and figure legends.
Data exclusions	In Figure 1M (set 2 immunoblots), one paired donor sample (Donor 1, D1) was excluded because the negative-control sample showed evidence of contamination/technical artefact; this donor was also identified as an outlier by statistical testing. In Figure 5H, one paired donor sample was excluded from the analysis because of technical issues affecting the GAPDH loading-control band in the negative-control sample. All exclusions are documented in the raw blot source-data file.
Replication	Key findings were independently reproduced across multiple biological systems and complementary experimental approaches. The effects of miR-31-5p modulation on CTR expression were confirmed using luciferase reporter assays, quantitative PCR, immunoblotting and RNA-binding protein immunoprecipitation. Fibrotic remodelling was assessed using scar-in-a-jar assays, immunoblotting, hydroxyproline assays, Masson's trichrome staining, gene-expression analyses and RNA sequencing, which yielded concordant results. Findings obtained in primary

human atrial fibroblasts from multiple independent donors were further validated in two independent mouse models (miR31/Cre and Lkb1-aKD) using histological, molecular, electrophysiological, telemetry and MRI-based approaches. RNA-sequencing findings were validated by quantitative PCR. Representative images and immunoblots shown in the figures were obtained from biologically independent samples included in the corresponding quantitative analyses. Independent repeat experiments reproduced the principal conclusions of the study. No reported findings failed independent replication; however, some individual readouts showed variability in effect size between experiments, as expected for studies using primary human cells derived from different donors.

Randomization

Randomization was not applicable to the human cohort, as participants were grouped according to clinical rhythm status (sinus rhythm or persistent atrial fibrillation). In animal studies, mice were assigned to experimental groups based on genotype and treatment. Where appropriate, animals were age- and sex-matched and compared with controls.

Blinding

Blinding was applied where feasible. Histological analyses, echocardiographic image analysis, and electrophysiological assessments were performed with investigators blinded to genotype and treatment group. Blinding was not applicable to all experimental procedures due to the nature of genetic and clinical group assignments.

Reporting for specific materials, systems and methods

We require information from authors about some types of materials, experimental systems and methods used in many studies. Here, indicate whether each material, system or method listed is relevant to your study. If you are not sure if a list item applies to your research, read the appropriate section before selecting a response.

Materials & experimental systems

n/a	Involved in the study	
☐	☒	Antibodies
☐	☒	Eukaryotic cell lines
☒	☐	Palaeontology and archaeology
☐	☒	Animals and other organisms
☐	☒	Clinical data
☒	☐	Dual use research of concern
☒	☐	Plants

Methods

n/a	Involved in the study	
☒	☐	ChIP-seq
☒	☐	Flow cytometry
☒	☐	MRI-based neuroimaging

Antibodies

Antibodies used

Anti-BAP1, Cell Signaling Technology, 13187S, clone D1W9B
Anti-CALCR, Bio-Rad, AHP635
Anti-CALCR, Bio-Rad, AHP3115
Anti-COL1, Merck, 234167
Anti-COL3A1, Novus Biologicals, NBP2-15946
Anti-FGF1, Abcam, ab207321, clone EPR19989
Anti-FLNA, Merck, MAB1680-C, clone TI10
Anti-FN1, Sigma, F3648
Anti-GAPDH (HRP-conjugated), Sigma, G9295, clone GAPDH-71.1
Anti-αSMA, Sigma, A5228, clone 1A4
Anti-STK40, Novus Biologicals, NBP1-33391
Anti-TNS1, Novus Biologicals, NBP1-84129
Clone identifiers are provided for monoclonal antibodies where available. Polyclonal antibodies do not have clone designations. Lot numbers were not recorded for all antibodies and are therefore not available.
Dilution factor for all primary antibodies was 1/1000, for secondary antibodies the dilution was 1/2500.

Validation

Anti-BAP1 - https://www.cellsignal.com/products/primary-antibodies/bap1-d1w9b-rabbit-monoclonal-antibody/13187
Anti-CALCR, AHP635 - https://www.bio-rad-antibodies.com/static/datasheets/ahp63/rat-calcitonin-receptor-antibody-ahp635.pdf; in house testing (PMID: 33149301)
Anti-CALCR, AHP3115 - https://images.bio-rad-antibodies.com/datasheets/datasheet-AHP3115.pdf
Anti-COL1, 234167 - https://www.sigmaaldrich.com/GB/en/product/mm/234167
Anti-COL3A1, NBP2-15946 - https://www.novusbio.com/products/collagen-iii-alpha-1-col3a1-antibody_nbp2-15946
Anti-FGF1, ab207321 - https://www.abcam.com/en-us/products/primary-antibodies/fgf1-antibody-epr19989-ab207321
Anti-FLNA, MAB1680-C - https://www.sigmaaldrich.com/GB/en/product/mm/mab1680c
Anti-FN1, F3648 - https://www.sigmaaldrich.com/GB/en/product/sigma/f3648
Anti-GAPDH (HRP-conjugated), G9295 - https://www.sigmaaldrich.com/GB/en/product/sigma/g9295
Anti-αSMA, Sigma, A5228 - https://www.sigmaaldrich.com/GB/en/product/sigma/a5228
Anti-STK40, NBP1-33391 - https://www.novusbio.com/products/stk40-antibody_nbp1-33391
Anti-TNS1, NBP1-84129 - https://www.novusbio.com/products/tensin-1-antibody_nbp1-84129

Eukaryotic cell lines

Policy information about [cell lines and Sex and Gender in Research]

Cell line source(s)

Primary human atrial cardiofibroblasts (ACFs) were isolated from atrial biopsies obtained from patients undergoing clinically

Cell line source(s)	indicated cardiac surgery at the John Radcliffe Hospital, Oxford, following informed consent. In addition, commercially available human primary atrial fibroblasts (CC-2903) were obtained from Lonza. HEK293 cells (ATCC CRL-1573) were used for reporter assays. The sex of primary human cell donors was not specified for individual experiments.
Authentication	HEK293 cells were obtained directly from the American Type Culture Collection (ATCC). No additional cell line authentication was performed. Primary human atrial fibroblasts were used at early passages (P3–P4) to minimize culture-induced phenotypic changes.
Mycoplasma contamination	Cell cultures were monitored by DAPI staining; no evidence of mycoplasma contamination was observed.
Commonly misidentified lines (See ICLAC register)	No commonly misidentified cell lines listed in the ICLAC register were used in this study.

Animals and other research organisms

Policy information about studies involving animals; ARRIVE guidelines recommended for reporting animal research, and Sex and Gender in Research

Laboratory animals	The study involved laboratory mice. A total of 175 mice were used, comprising Lkb1-aKD mice (n = 66) and miR31/Cre mice (n = 109). The study involved laboratory mice (Mus musculus). Lkb1fl/fl mice were obtained from The Jackson Laboratory (JAX strain #014143) and maintained on a C57BL/6J genetic background, where the suffix "J" denotes the C57BL/6J substrain maintained by The Jackson Laboratory. The fibroblast-specific tamoxifen-inducible Col1a2-CreERT strain (Tg(Col1a2-cre/ERT,-ALPP)7Cpd/J) was obtained from The Jackson Laboratory and maintained on a C57BL/6J background. Pre-miR31-Inv transgenic mice were generated by the Davies Laboratory, University of Oxford (WTCHG Transgenic Core Facility), in C57BL/6J mice. Unless otherwise stated, experiments using miR31/Cre mice were performed in age-matched 12-week-old male and female animals. For the Lkb1-aKD model, experiments were performed in either 12-week-old mice (young cohort) or 24-week-old mice (MRI cohort), as indicated in the corresponding Methods sections, figures and figure legends. Mice were housed under a 12 h light/12 h dark cycle (06:00-18:00) at 68-72°F (20-22°C) and approximately 50% humidity. Cardiac MRI was performed in a subset of 24-week-old Lkb1-aKD mice using a Bruker Biospec 9.4T scanner and the collagen-targeted contrast agent EP3533 for longitudinal assessment of atrial fibrosis.
Wild animals	This study did not involve wild animals.
Reporting on sex	Animals of both sexes were included in all experiments. Experimental groups were age-matched and compared with genotype- and treatment-appropriate controls. Male and female animals were analysed together unless otherwise indicated; sex-stratified analyses were performed for selected experiments and are reported in the figures, figure legends and supplementary data.
Field-collected samples	This study did not involve samples collected from the field.
Ethics oversight	All animal breeding, handling, and experimental procedures were approved and conducted in accordance with local Animal Care and Ethics Committee guidance at Baylor College of Medicine (USA) and the University of Oxford (UK). Animal studies were performed in compliance with the UK Home Office Animals (Scientific Procedures) Act 1986, incorporating Directive 2010/63/EU of the European Parliament, and in accordance with NIH guidelines.

Note that full information on the approval of the study protocol must also be provided in the manuscript.

Clinical data

Policy information about clinical studies

All manuscripts should comply with the ICMJE guidelines for publication of clinical research and a completed CONSORT checklist must be included with all submissions.

Clinical trial registration	This study was not a clinical trial and therefore was not registered in a clinical trials registry.
Study protocol	This was a prospective observational study of patients undergoing elective cardiac surgery. Participants were classified according to rhythm status (sinus rhythm or persistent atrial fibrillation) and atrial tissue was collected for downstream molecular analyses.
Data collection	Human atrial tissue and associated clinical data were collected from patients undergoing elective cardiac surgery following written informed consent and approval by the South Central Berkshire B Research Ethics Committee (18/SC/0404). Participants were classified according to rhythm status (sinus rhythm or persistent atrial fibrillation). A total of 145 patients were included (70 SR and 75 persistent AF). Age and sex distributions are reported in Table 2 of the manuscript. Participants were recruited from a single tertiary referral cardiac surgery centre, and because enrolment was restricted to patients undergoing clinically indicated cardiac surgery, the cohort may not fully represent the broader atrial fibrillation population.
Outcomes	Molecular, cellular and histological measures of calcitonin receptor signalling, atrial fibrosis and arrhythmia-related phenotypes in human samples and experimental models. This study was not a clinical trial.

Plants

Seed stocks	N/A
Novel plant genotypes	N/A
Authentication	N/A
