# Supplementary material for: Selective blockade of microRNA-31-5p−calcitonin receptor interaction reverses established atrial fibrosis and atrial arrhythmia burden
Source: Nat Cardiovasc Res. 2026 Oct 30;5(11):1231–49. doi: 10.1038/s44161-026-00897-1 (PMC13759305; doi:10.1038/s44161-026-00897-1)
Supplement: Supplementary file 2 [file 44161_2026_897_MOESM8_ESM.pdf]

Figure 1J

Raw blots – set 1

Fig. 1J_miR31 mimic hACFs

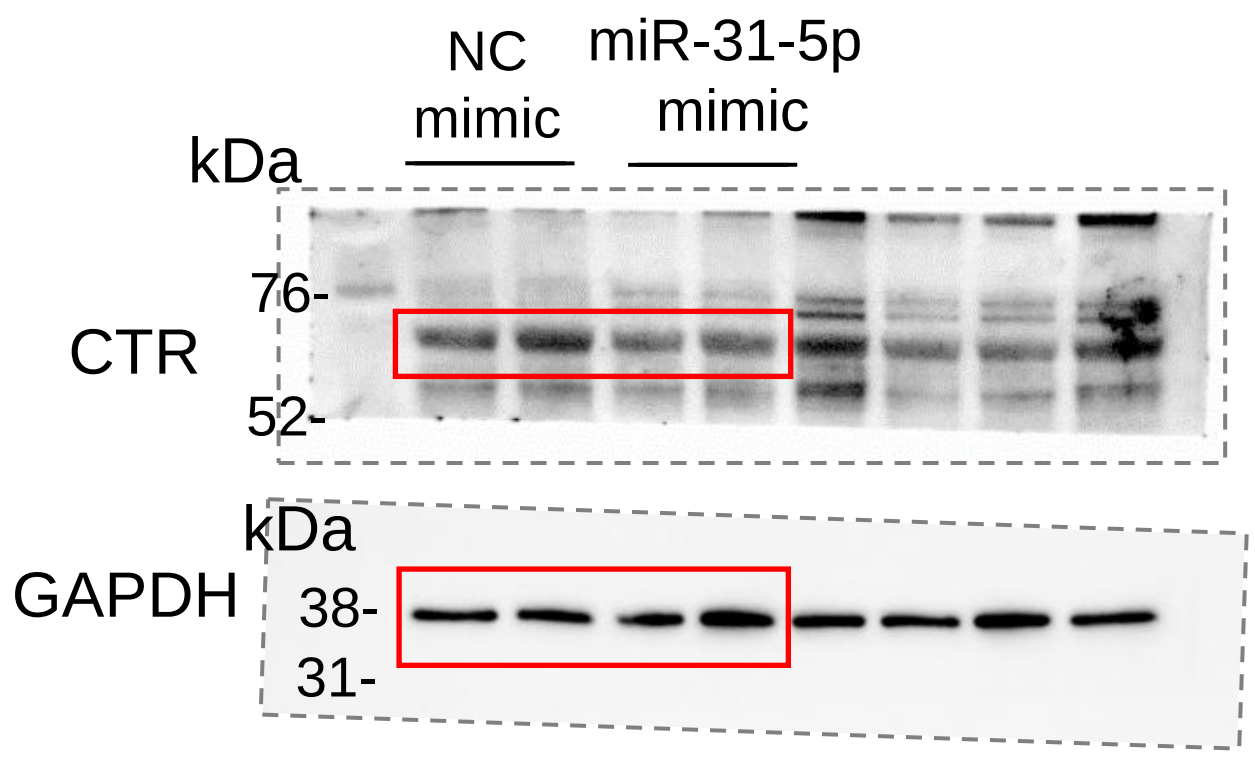

Raw blots – set 2

Fig. 1J_miR31 mimic hACFs

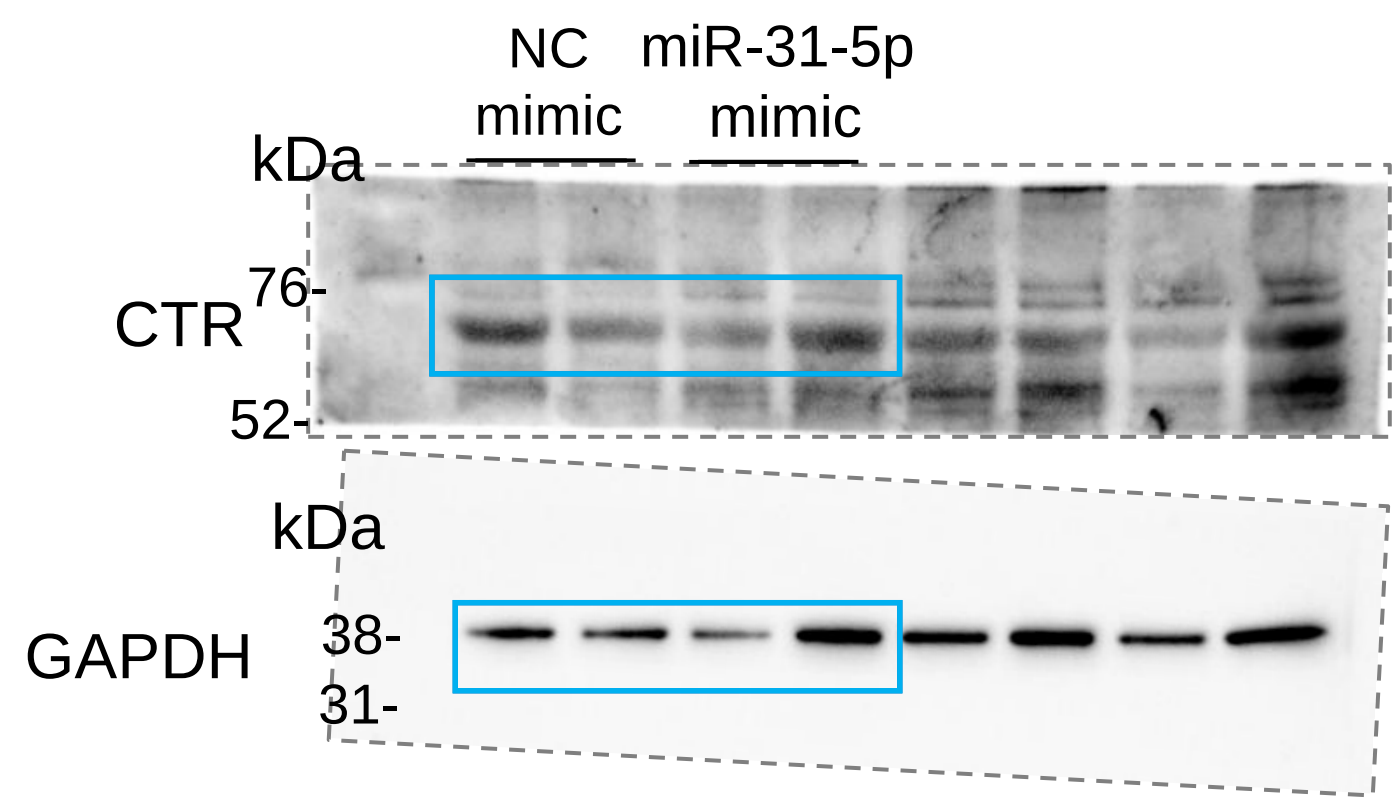

Raw blots – set 3

Fig. 1J_miR31 mimic hACFs

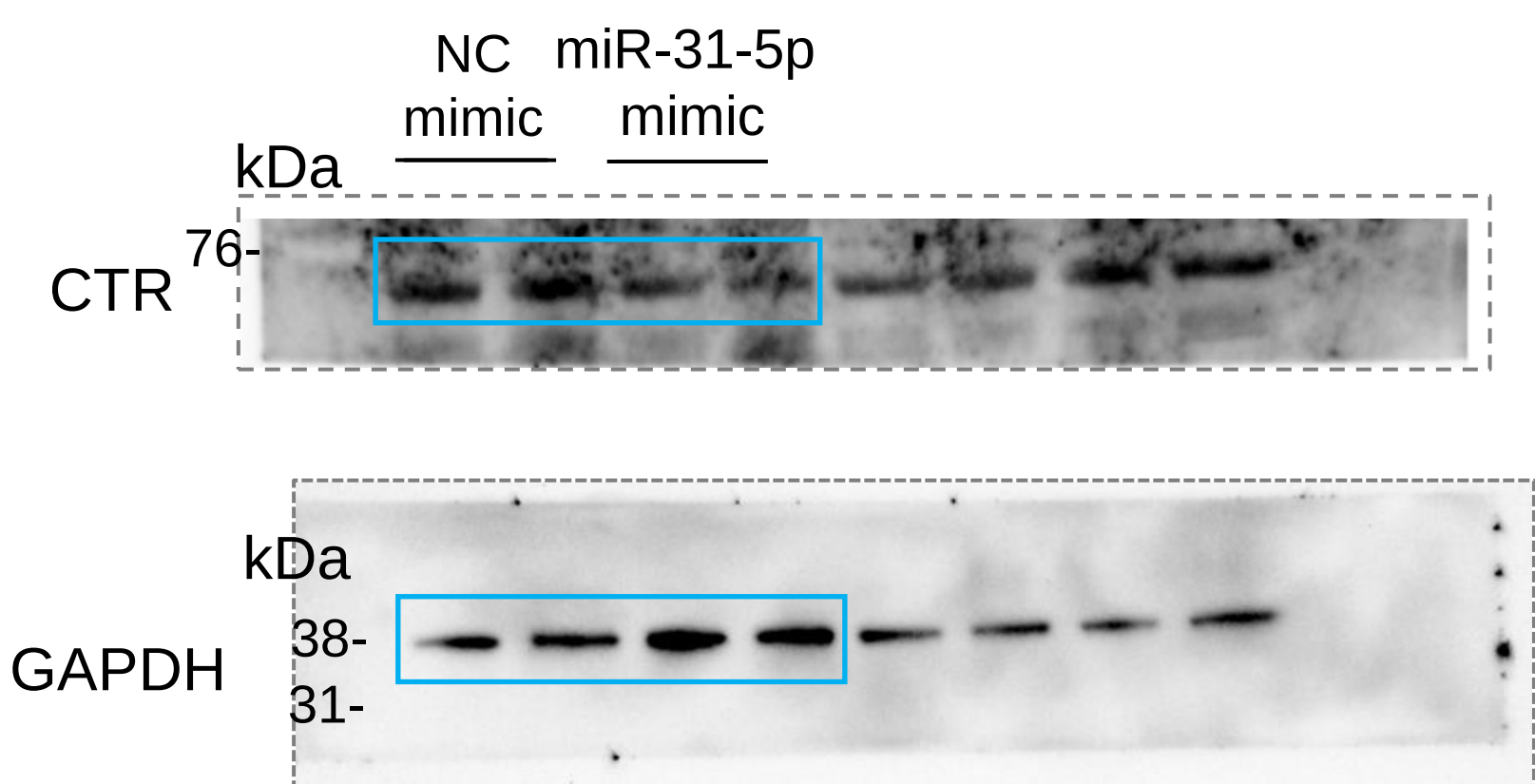

In red - included in the manuscript figure panels and quantification
In blue - included in the quantification graphs
Grey dotted borders: full uncropped blot scans.

Figure 1M

Raw blots – set 1

Fig. 1M_miR31inh. hACFs

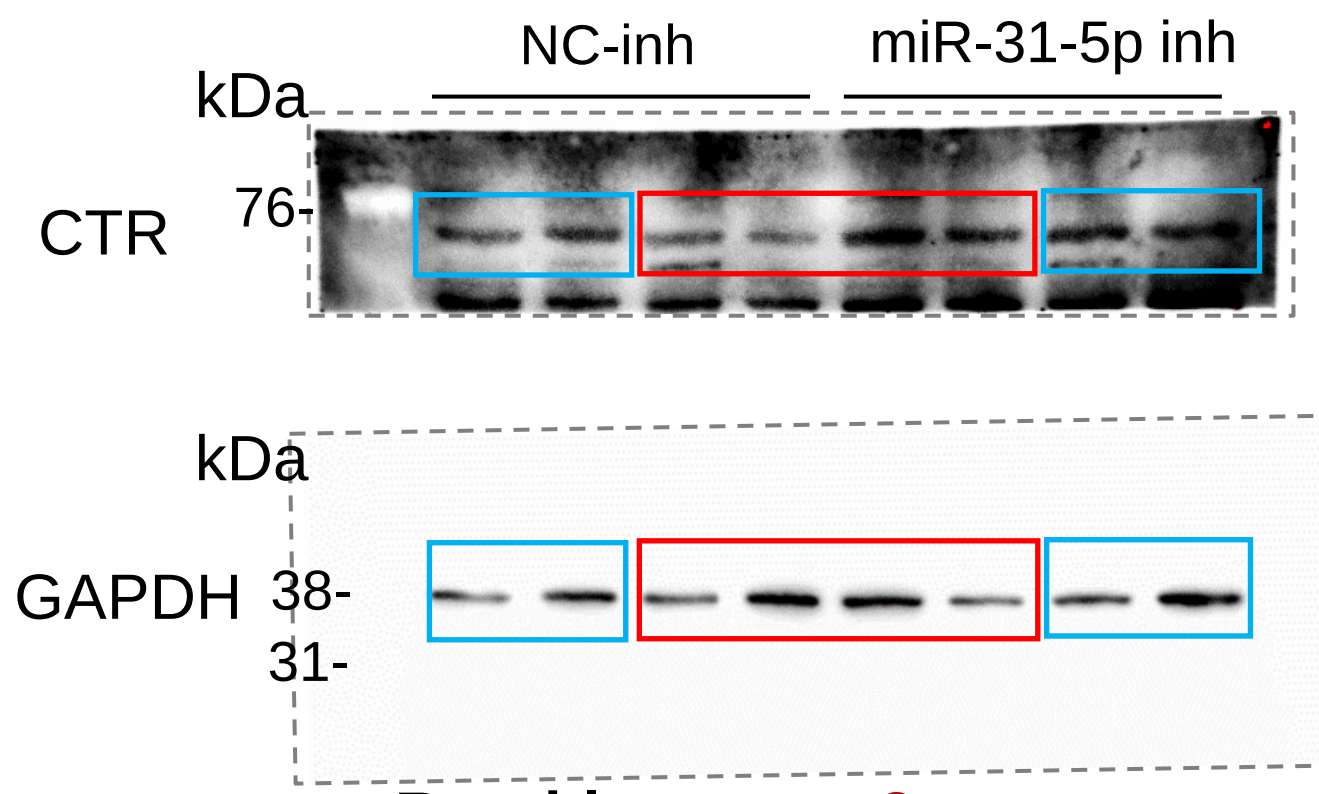

Raw blots – set 2

Fig. 1M_miR31inh. hACFs

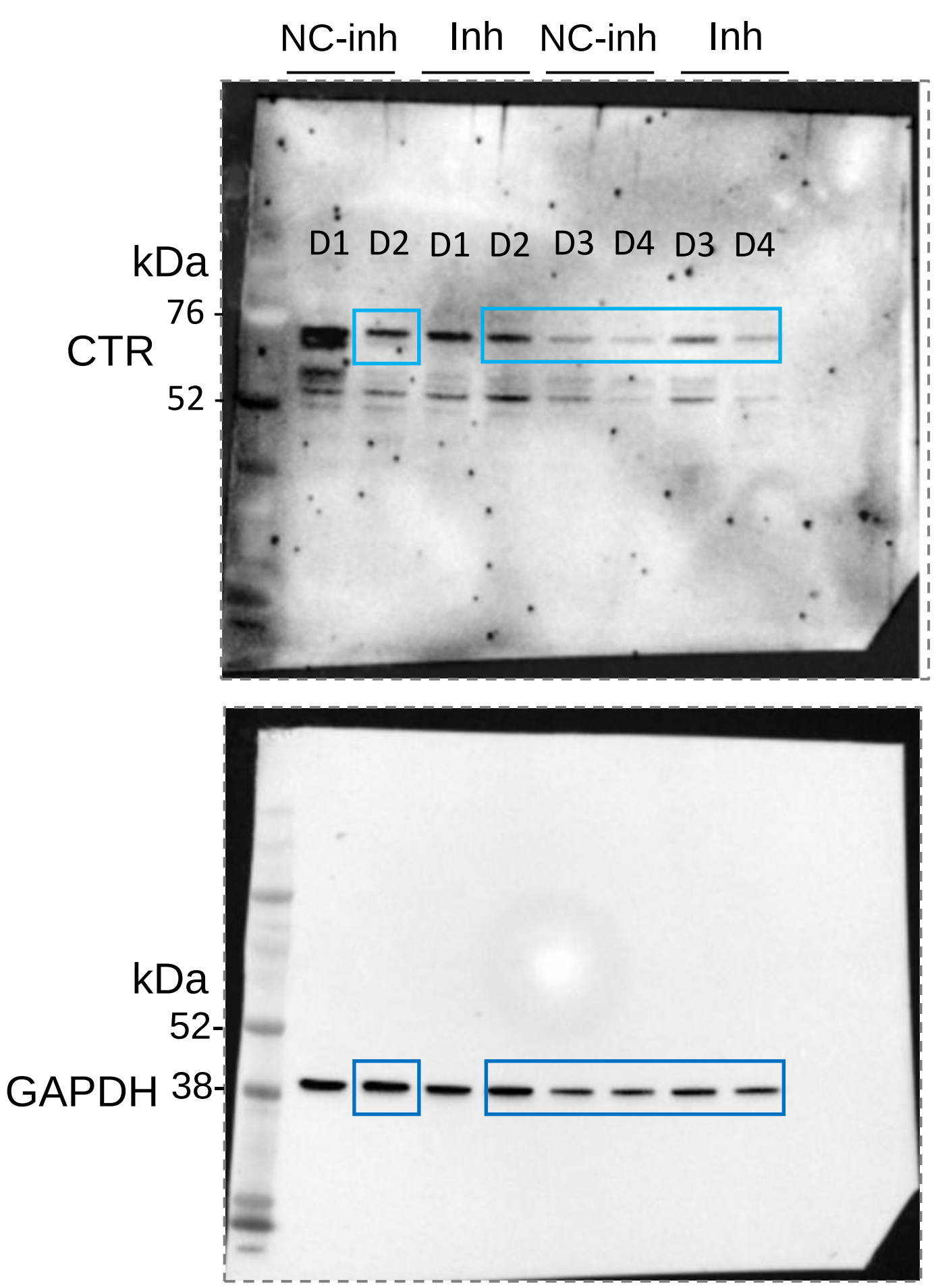

In red - included in the manuscript figure panels and quantification
In blue - included in the quantification graphs
Grey dotted borders: full uncropped blot scans
* Raw blots - set 2: Donor 1 (D1) paired samples have been excluded from the analysis due to suspected contamination in NC; the outlier test for this donor is also positive.

Figure 3E

Raw blots – set 1
Fig. 3E_αSMA in hACFs

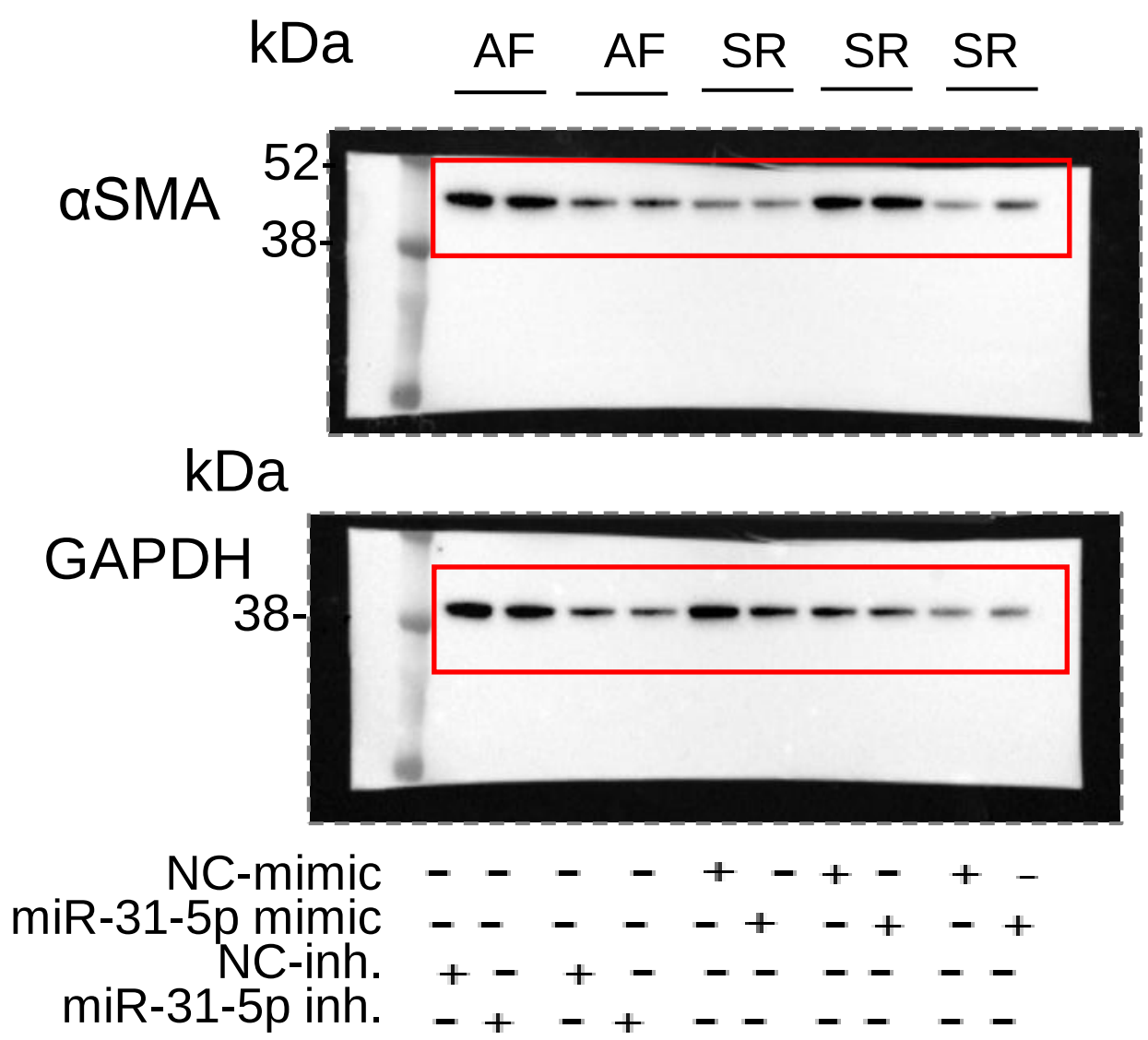

Raw blots – set 2
Fig. 3E_αSMA in hACFs

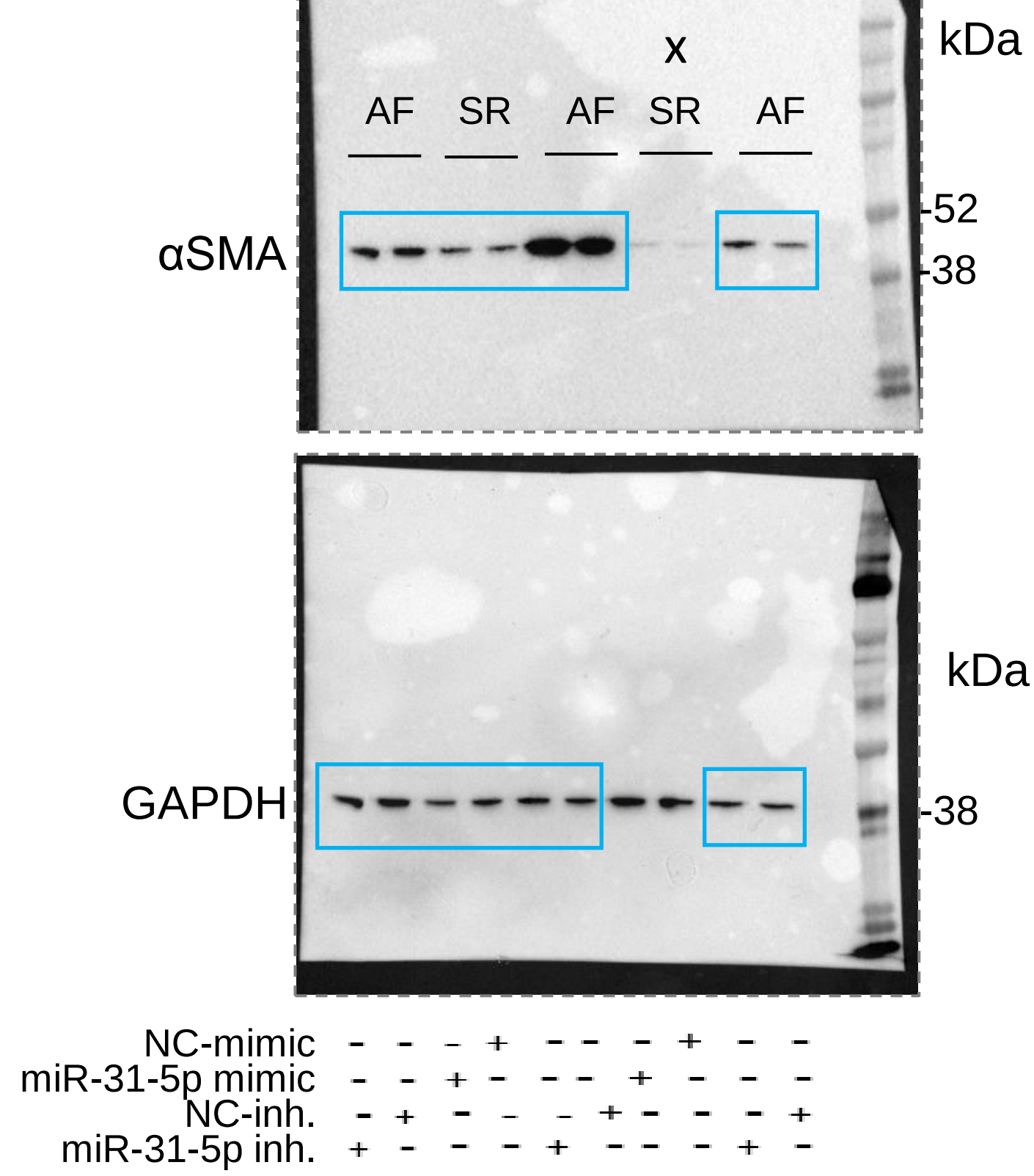

In red - included in the manuscript figure panels and quantification
In blue - included in the quantification graphs
Grey dotted borders: full uncropped blot scans.
X - repeated donor (not included)
Inh – inhibitor

Figure 4M

Raw blots – set 1

Fig. 4M_TSB Lkb1-aKD mice

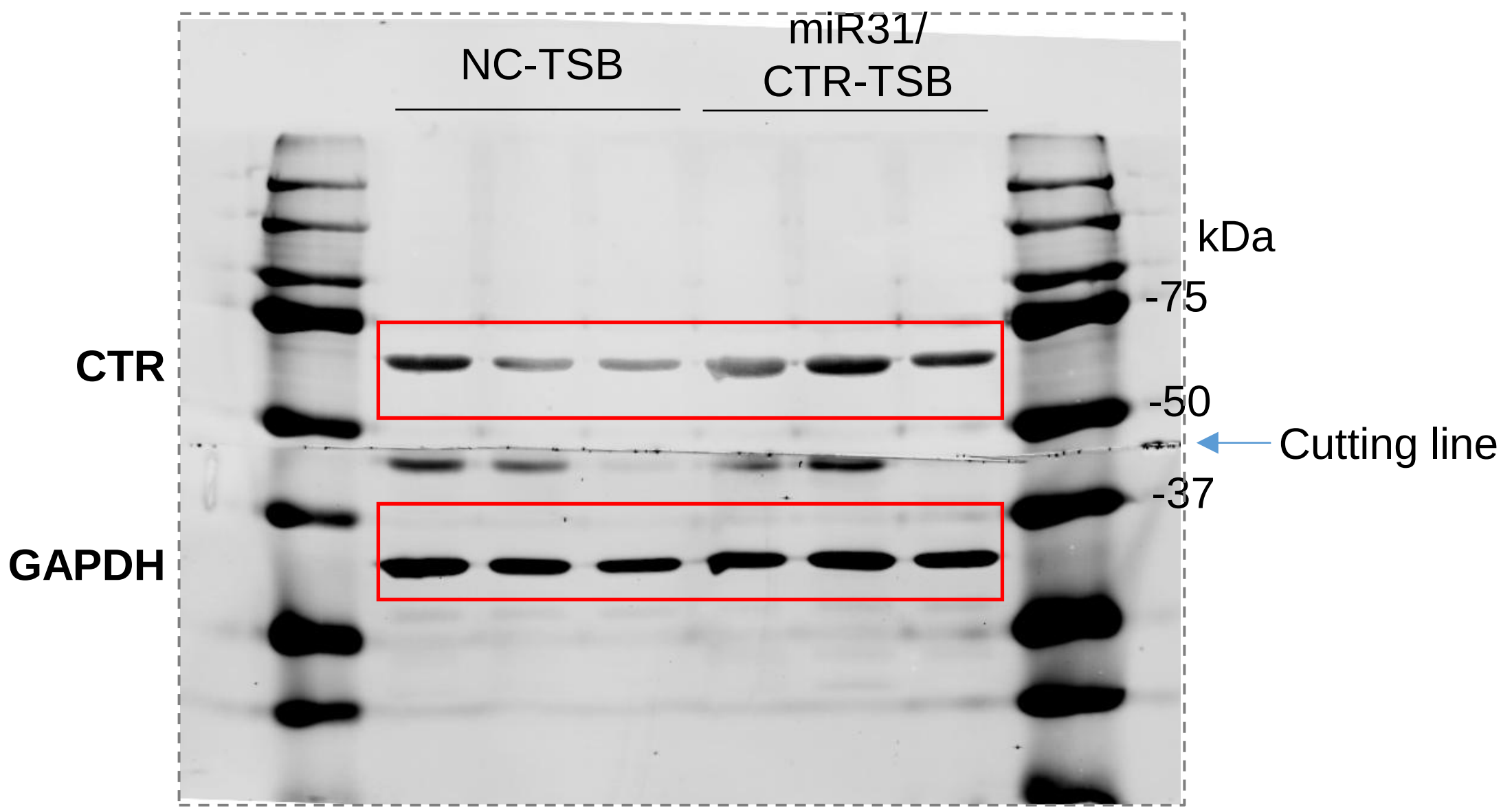

Raw blots – set 2

Fig. 4M_TSB Lkb1-aKD mice

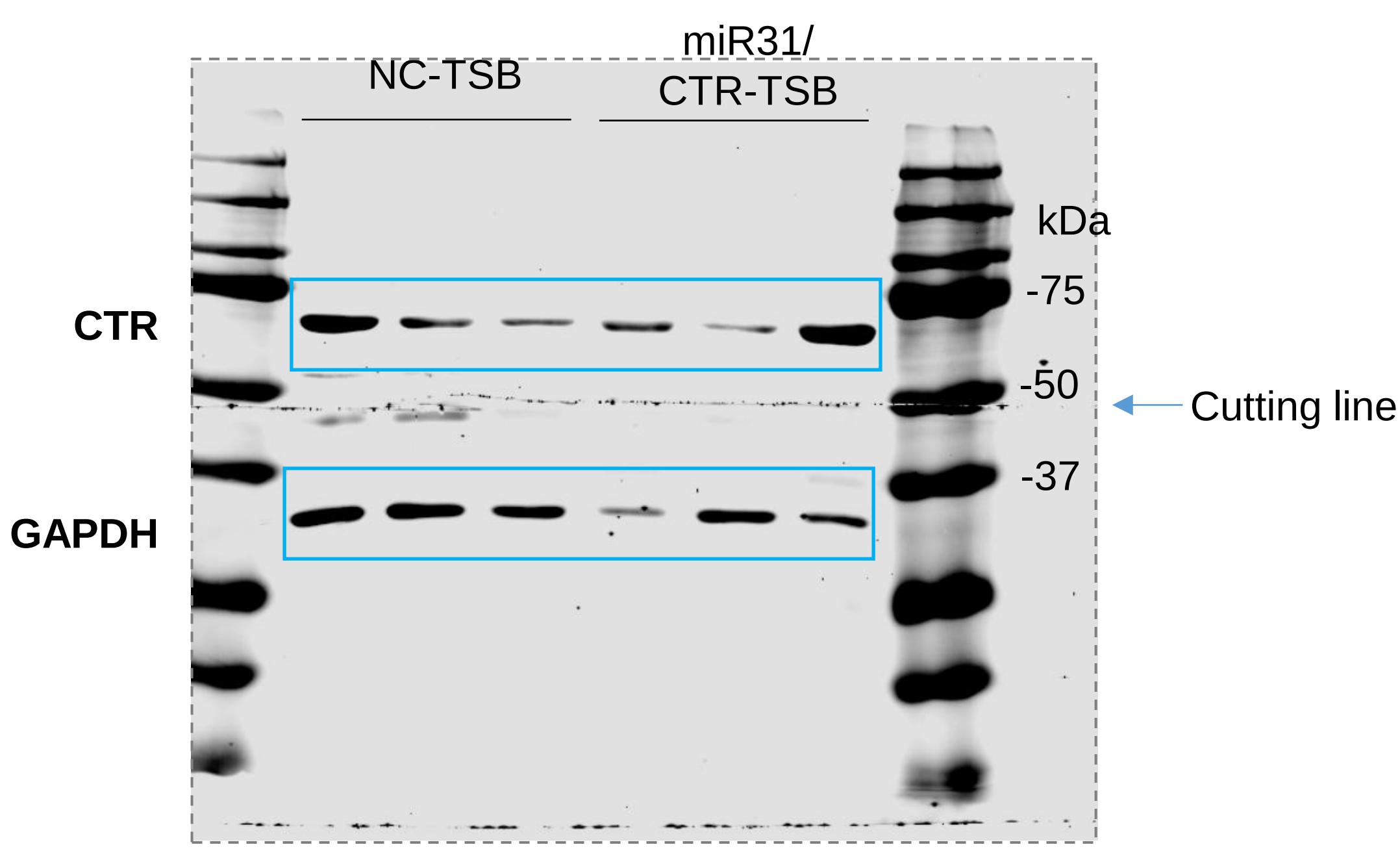

In red - included in the manuscript figure panels and quantification
In blue - included in the quantification graphs
Grey dotted borders: full uncropped blot scans.

Figure 5H

Raw blots – set 1

Fig. 5H_TSB in hACFs

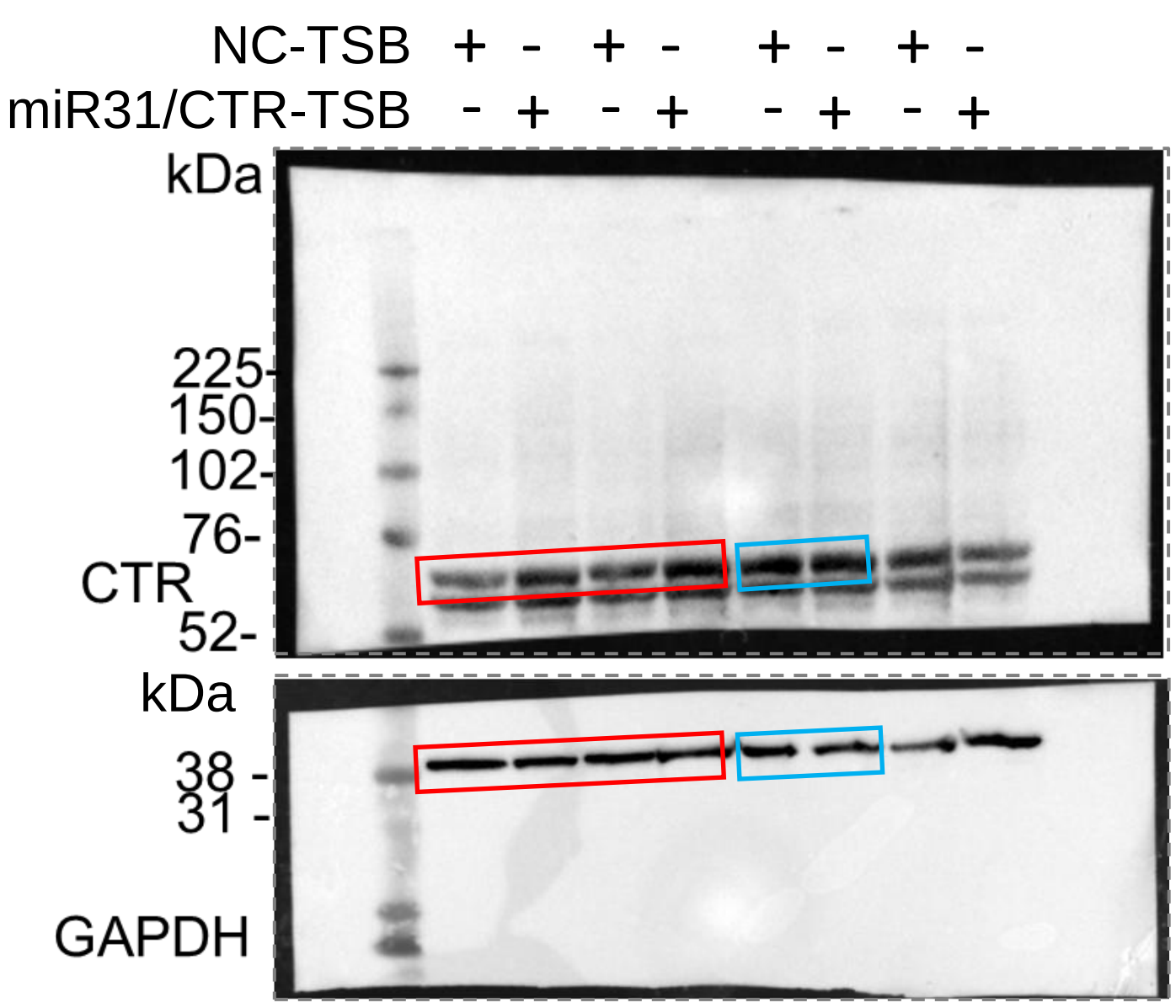

Raw blots – set 2

Fig. 5H_TSB in hACFs

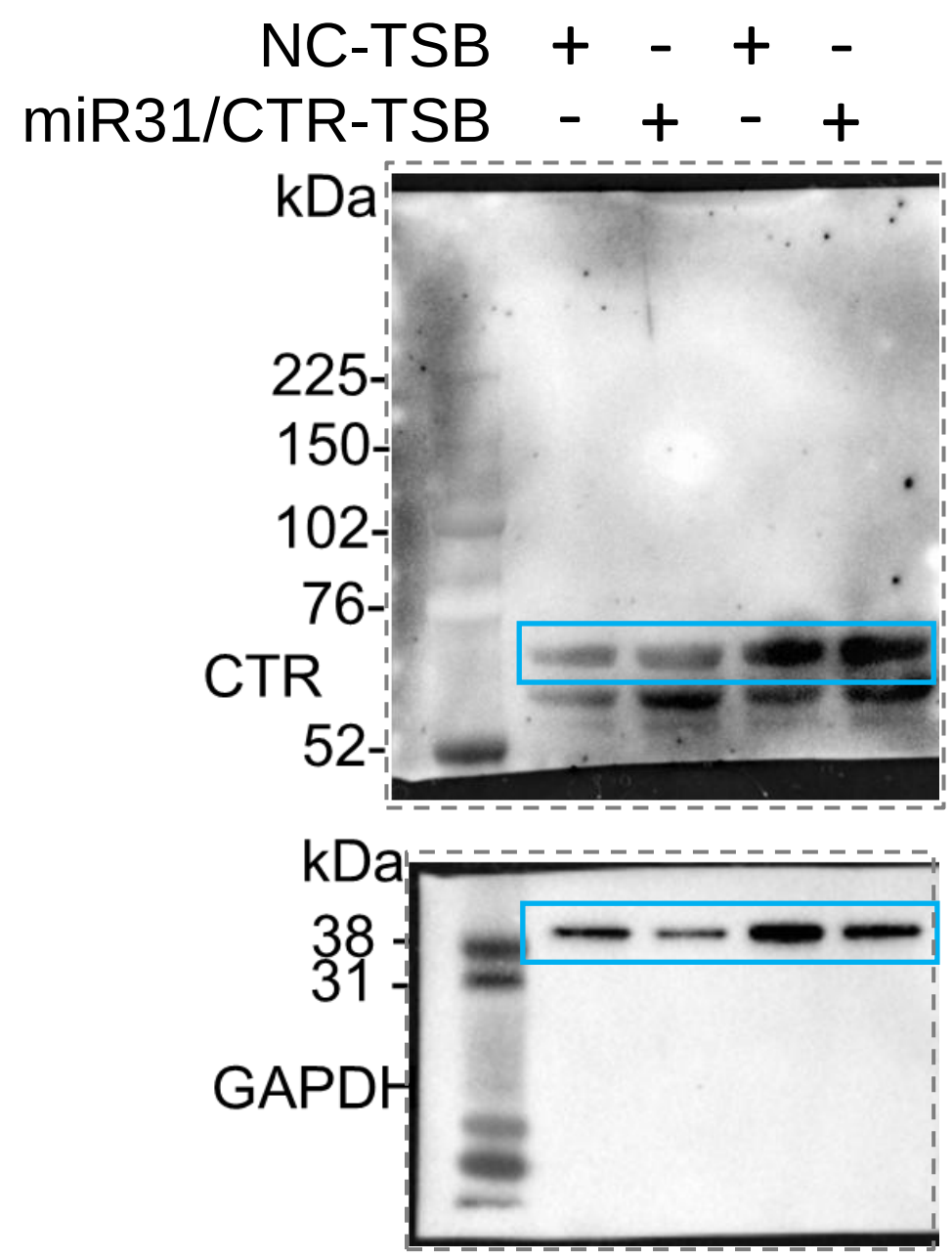

In red - included in the manuscript figure panels and quantification
In blue - included in the quantification graphs
Grey dotted borders: full uncropped blot scans.
* Raw blot - set1: the last paired donor was not included in the analysis due to technical issues in GAPDH band in NC-treated donor's cells.

Figure 5I

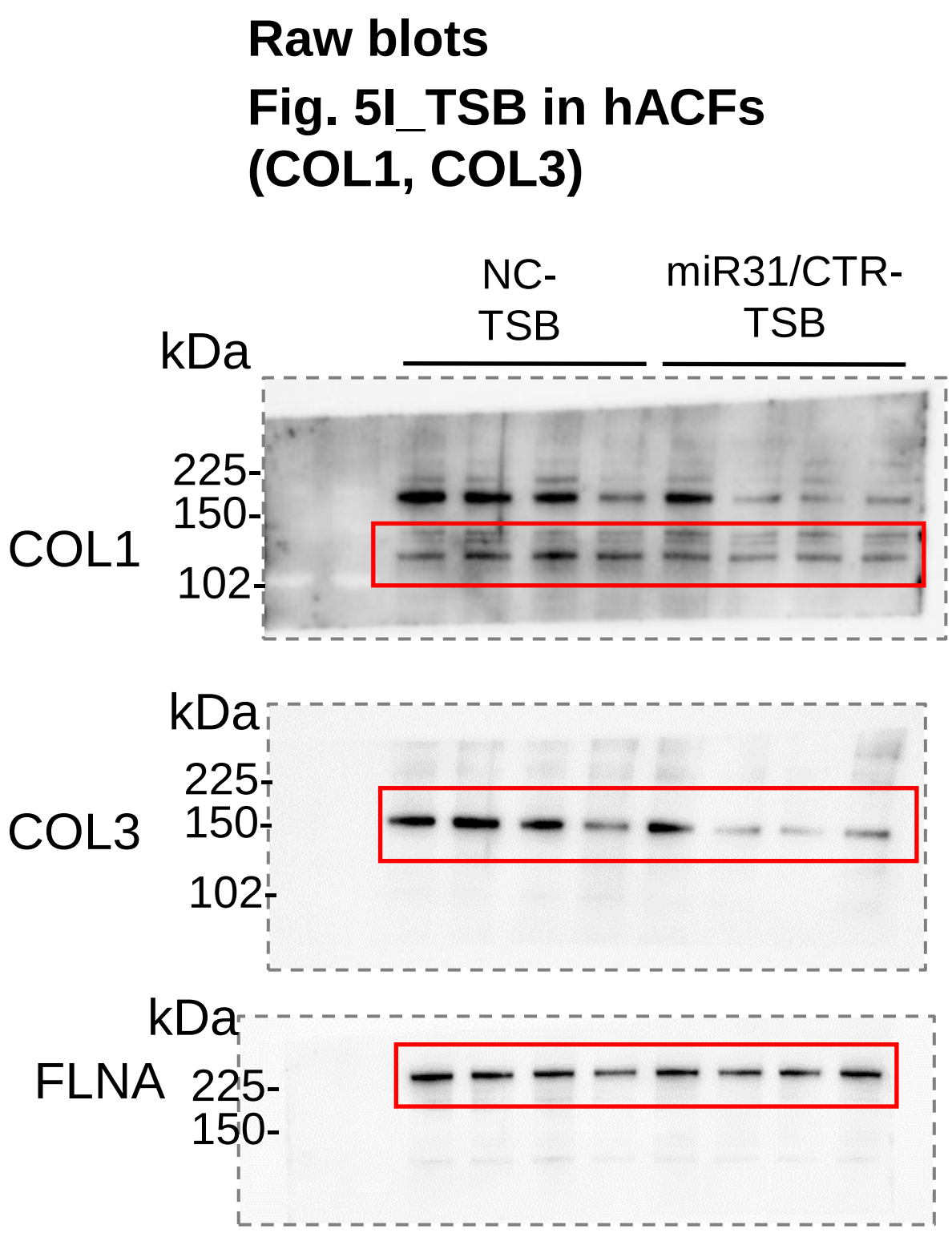

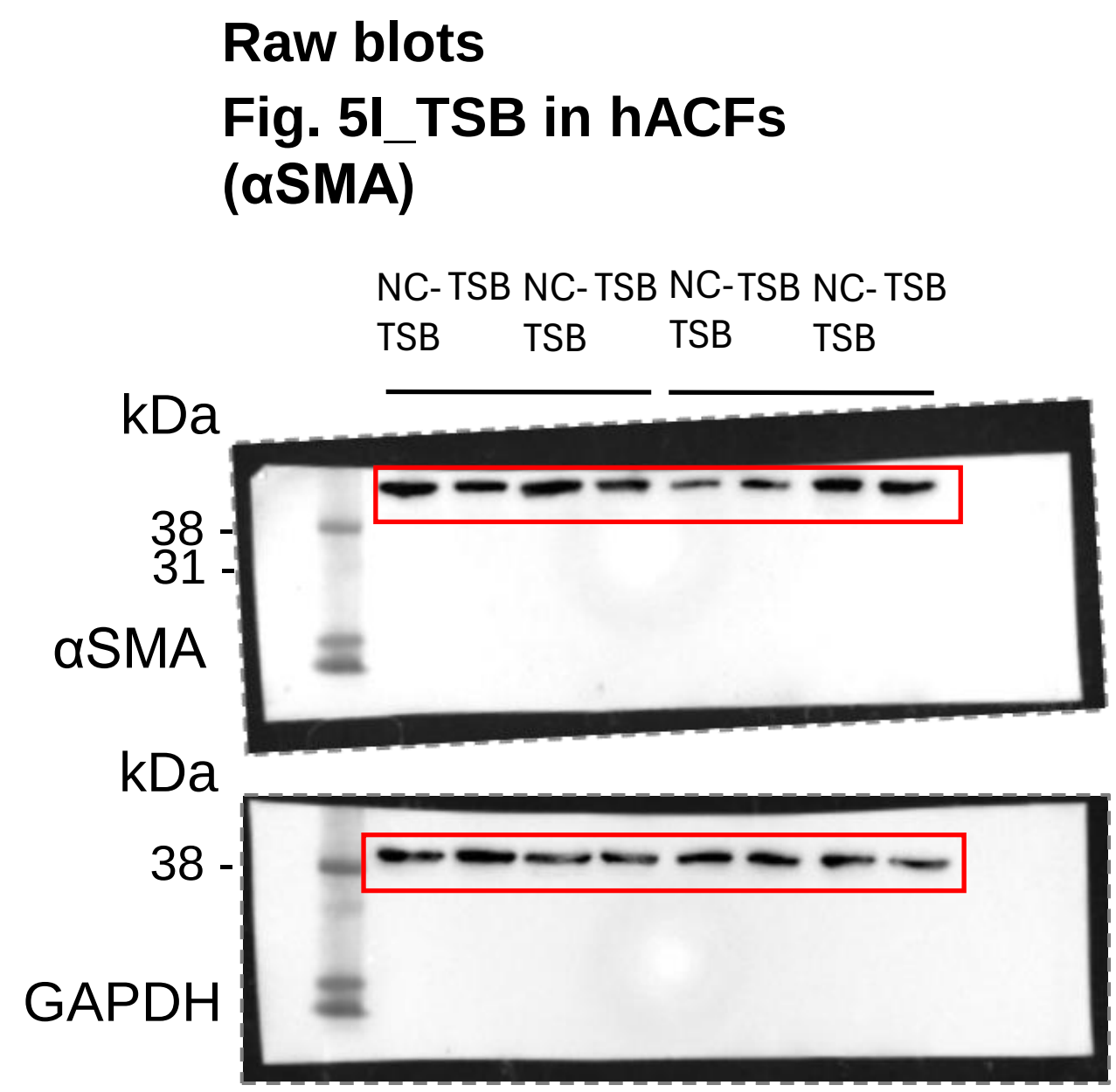

In red - included in the manuscript figure panels and quantification
Grey dotted borders: full uncropped blot scans.

Extended Data Figure 2B

Raw PCR gel – set 1

miR31-TG Heterozygous X WT example litter

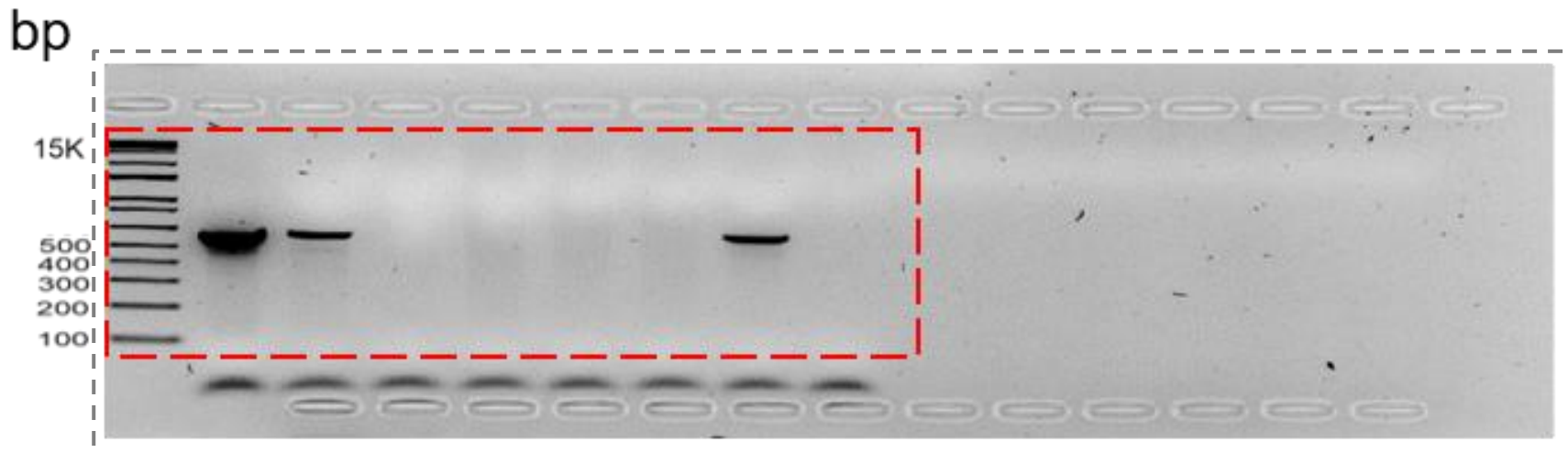

Raw PCR gel – set 1

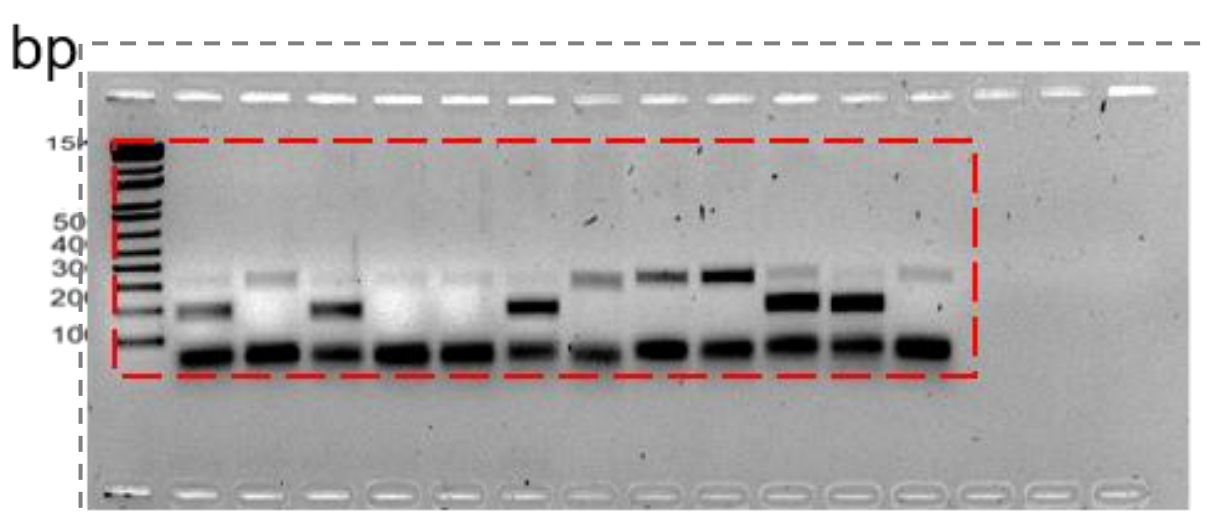

Raw PCR gel – set 1

Col1a2-Cre-ER(T)-Tg Hemizygous X WT example litter

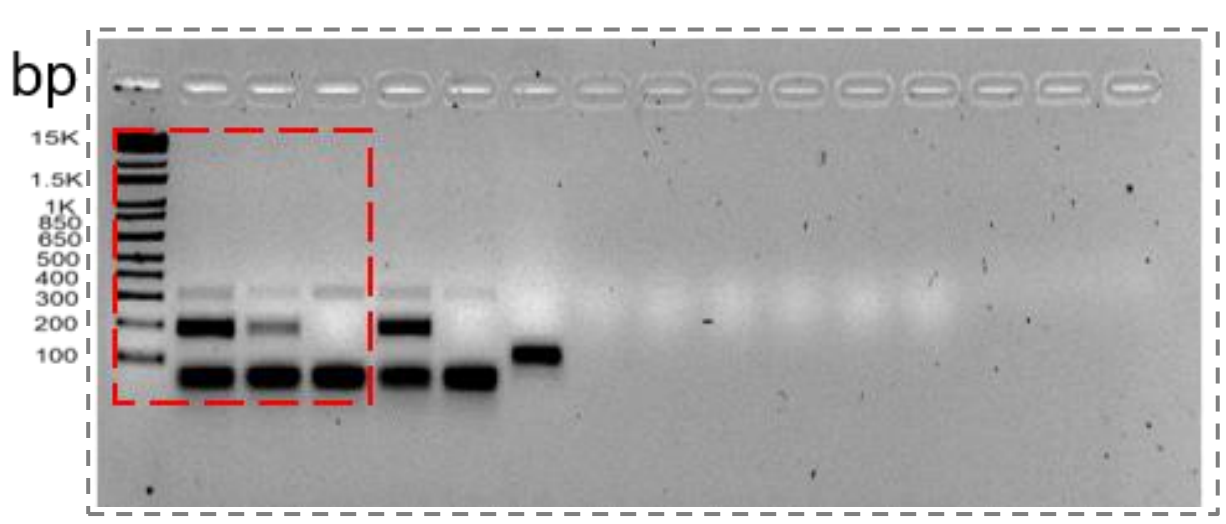

Red sections are included in the manuscript figure panels
Grey dotted borders: full uncropped blot scans

Extended Data Figure 2C

Raw PCR gel

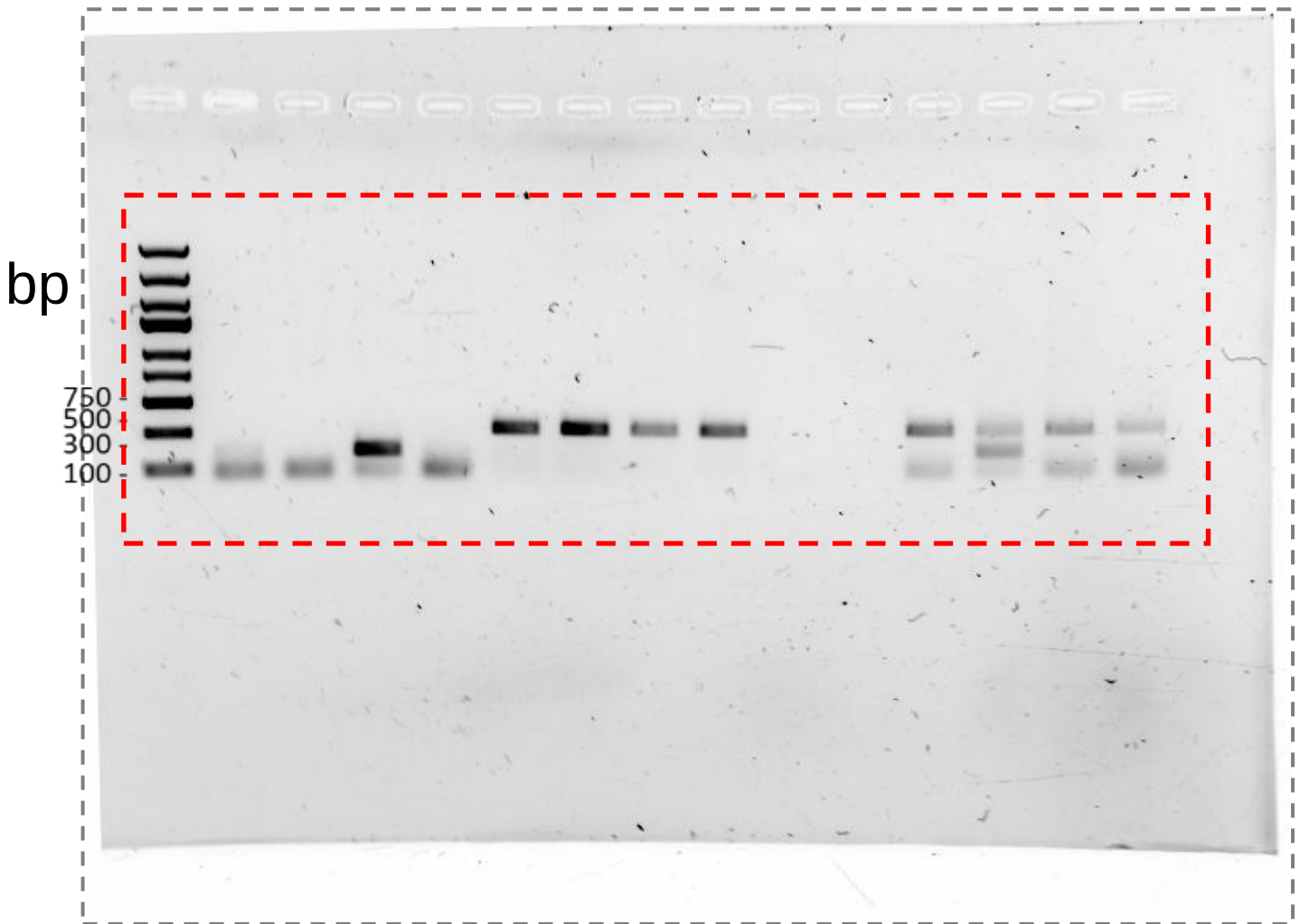

Red sections are included in the manuscript figure panels
Grey dotted borders: full uncropped blot scans

Extended Data Figure 3A

Raw PCR gel – set 1

Panel A

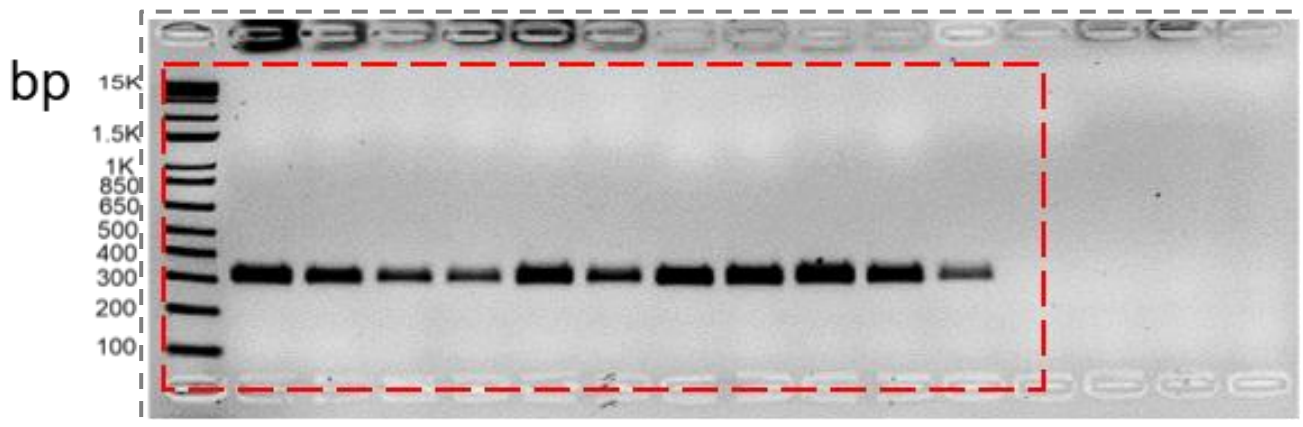

Raw PCR gel – set 1

Panel D

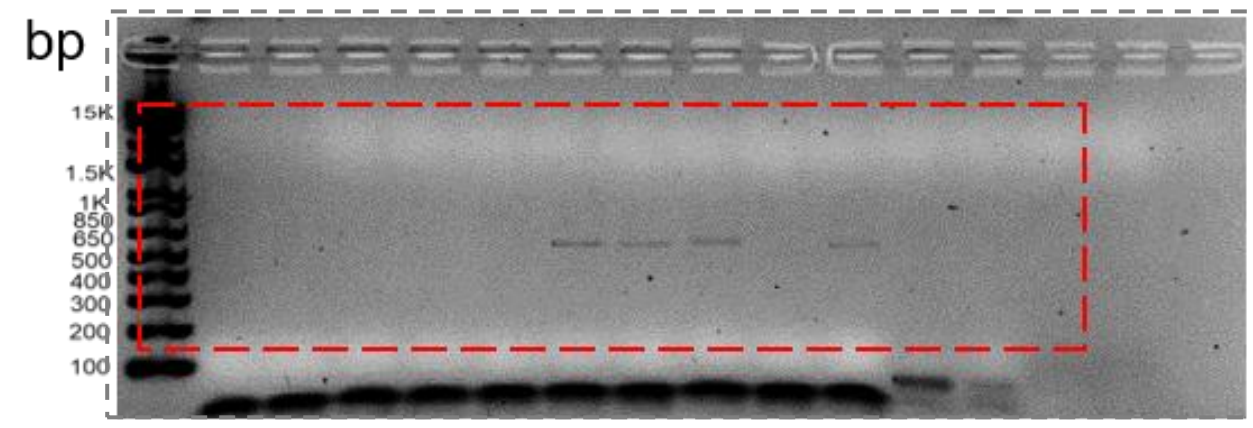

Raw PCR gel – set 1

Panel B

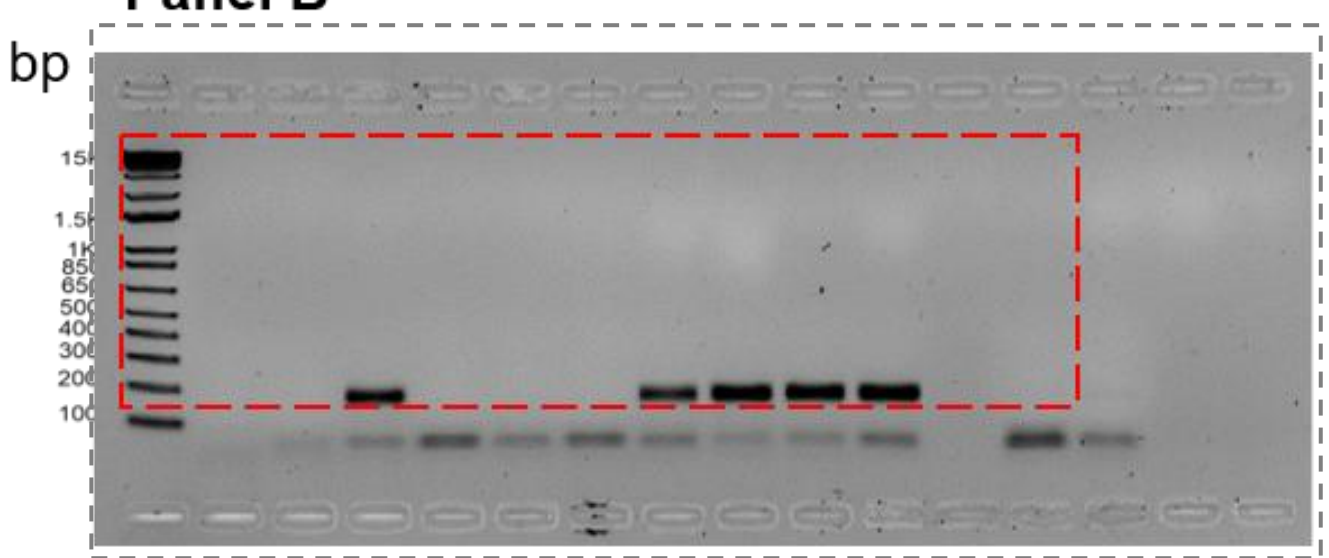

Raw PCR gel – set 1

Panel E

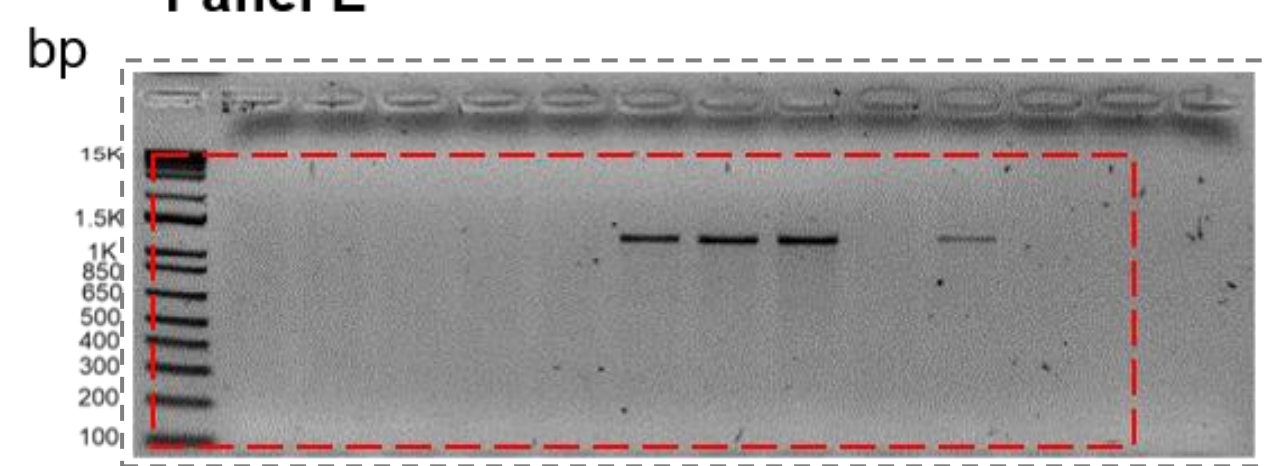

Raw PCR gel – set 1

Panel C

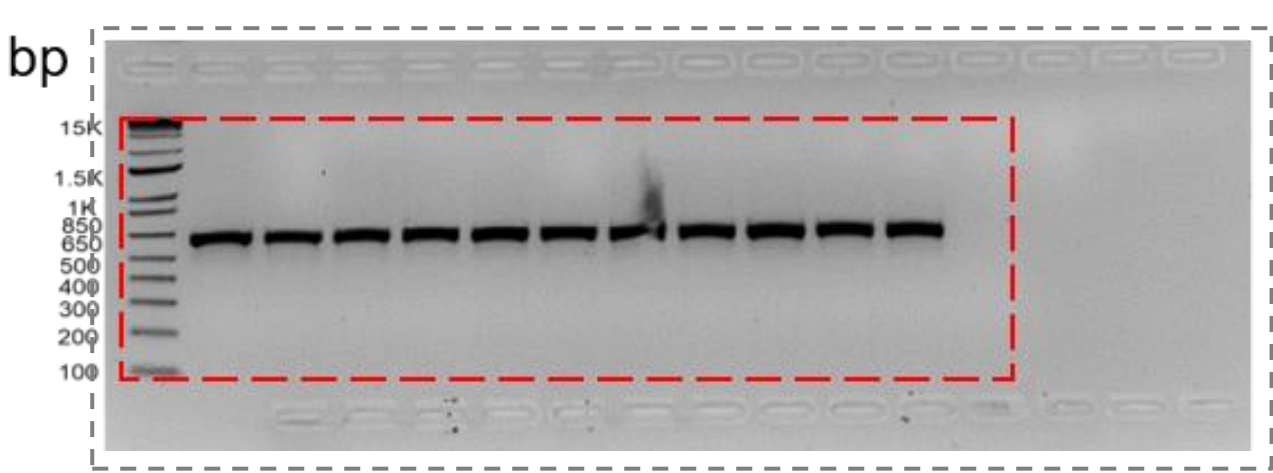

Red sections are included in the manuscript figure panels
Grey dotted borders: full uncropped blot scans

Extended Data Figure 3A

Raw PCR gel – set 1
Panel F

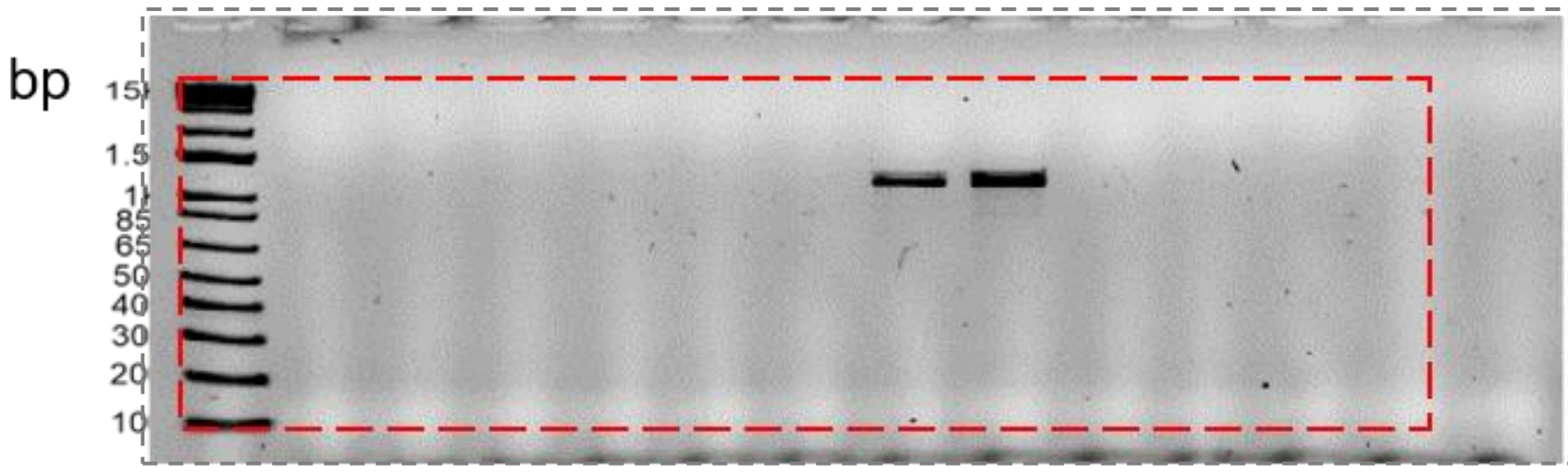

Raw PCR gel – set 1
Panel G

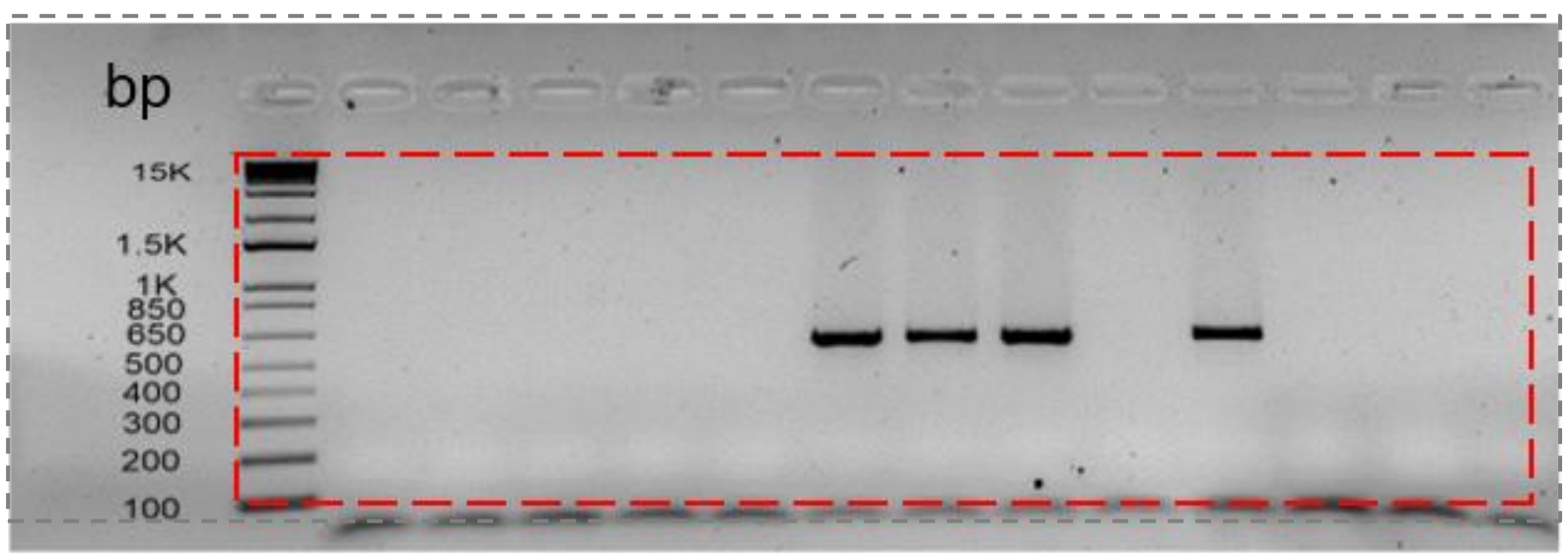

Raw PCR gel – set 1
Panel H

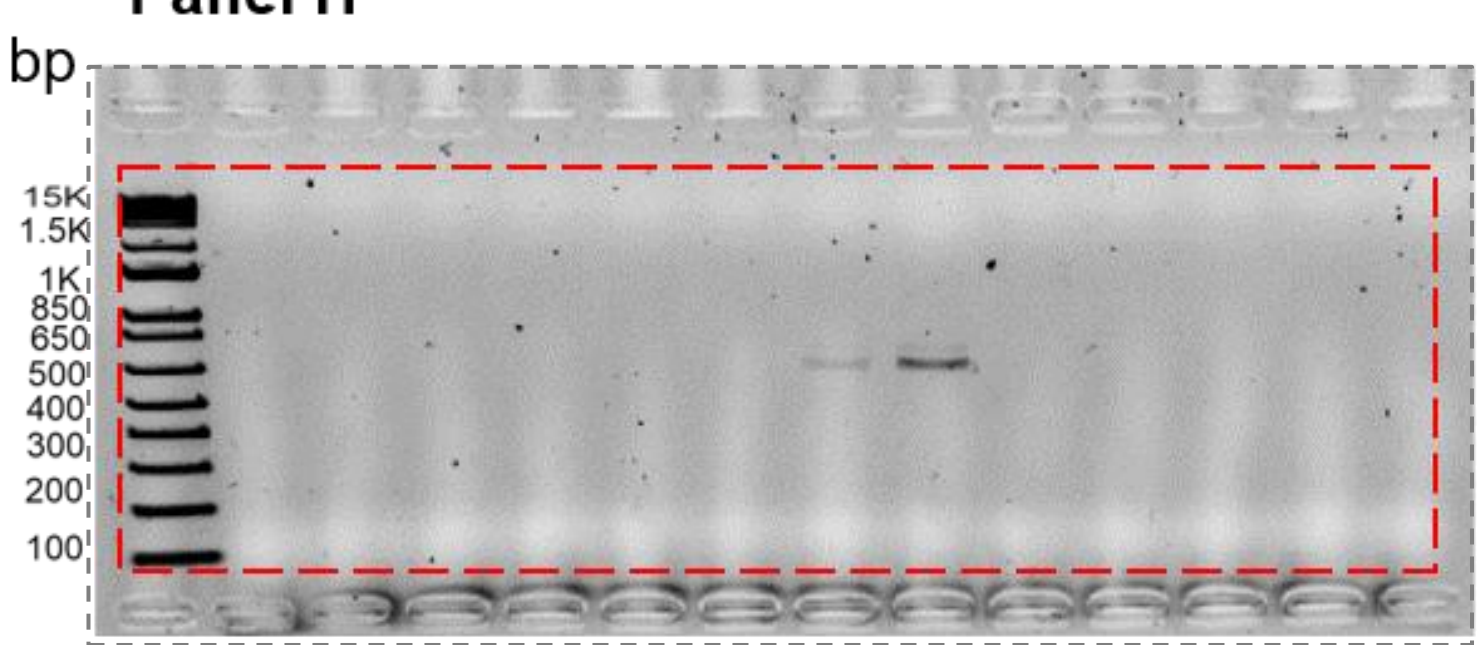

Raw PCR gel – set 1
Panel I

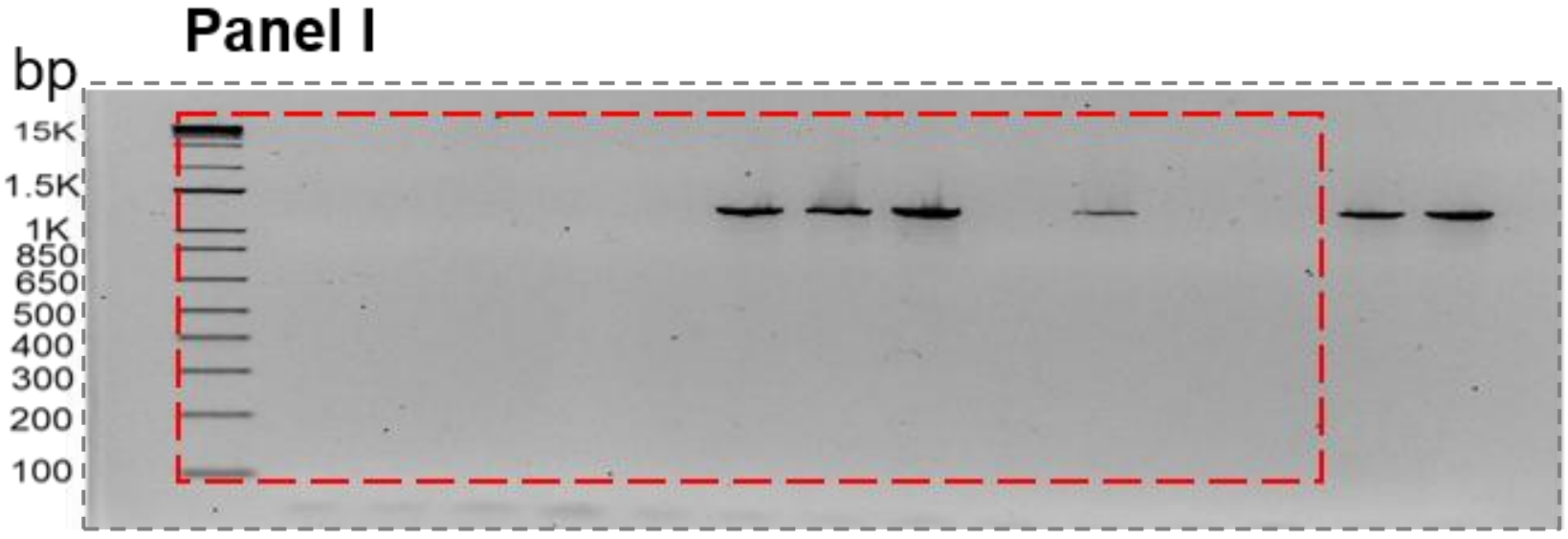

Red sections are included in the manuscript figure panels
Grey dotted borders: full uncropped blot scans

Extended Data Figure 3A

Raw PCR gel – set 1
Panel J

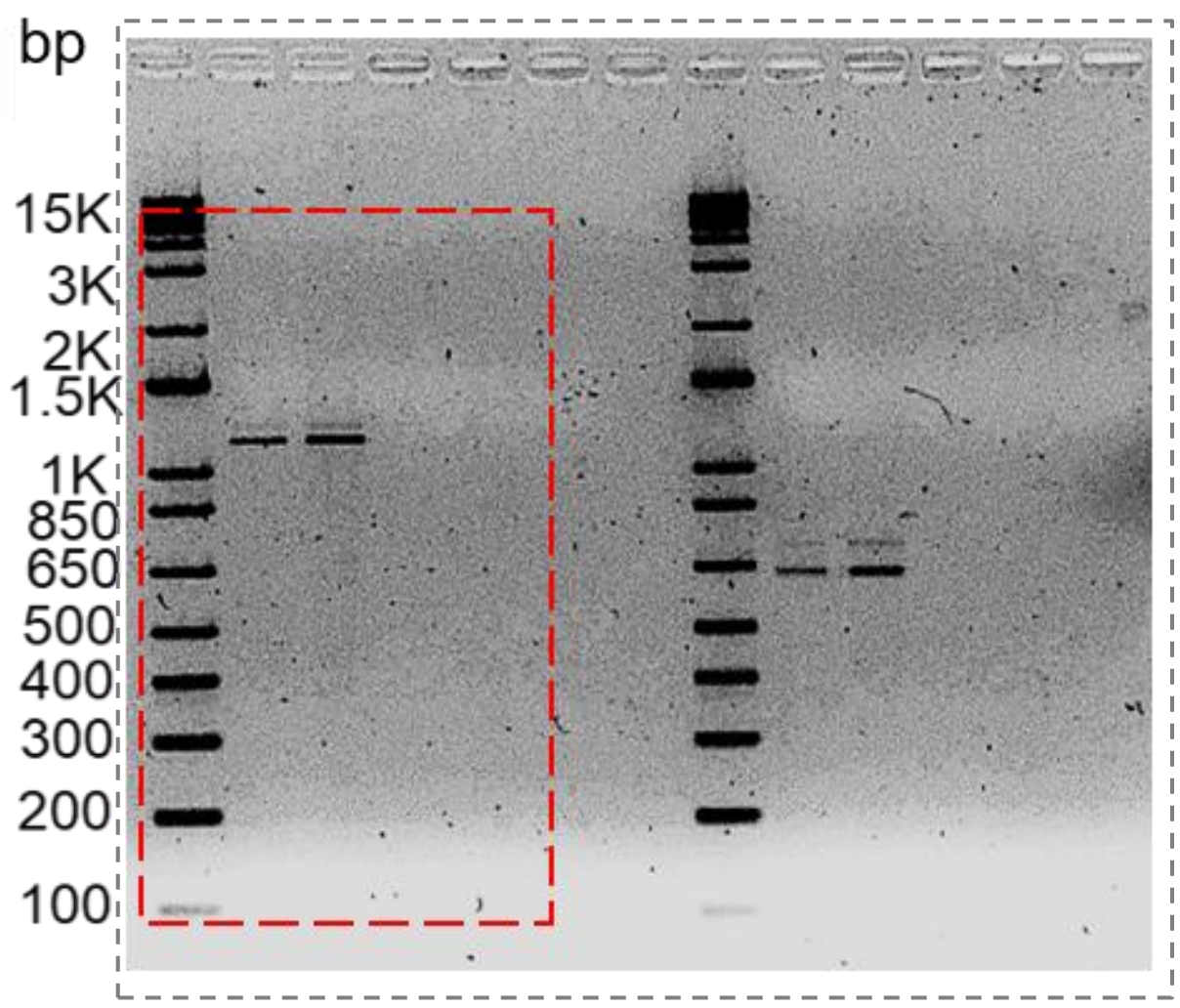

Raw PCR gel – set 1
Panel K

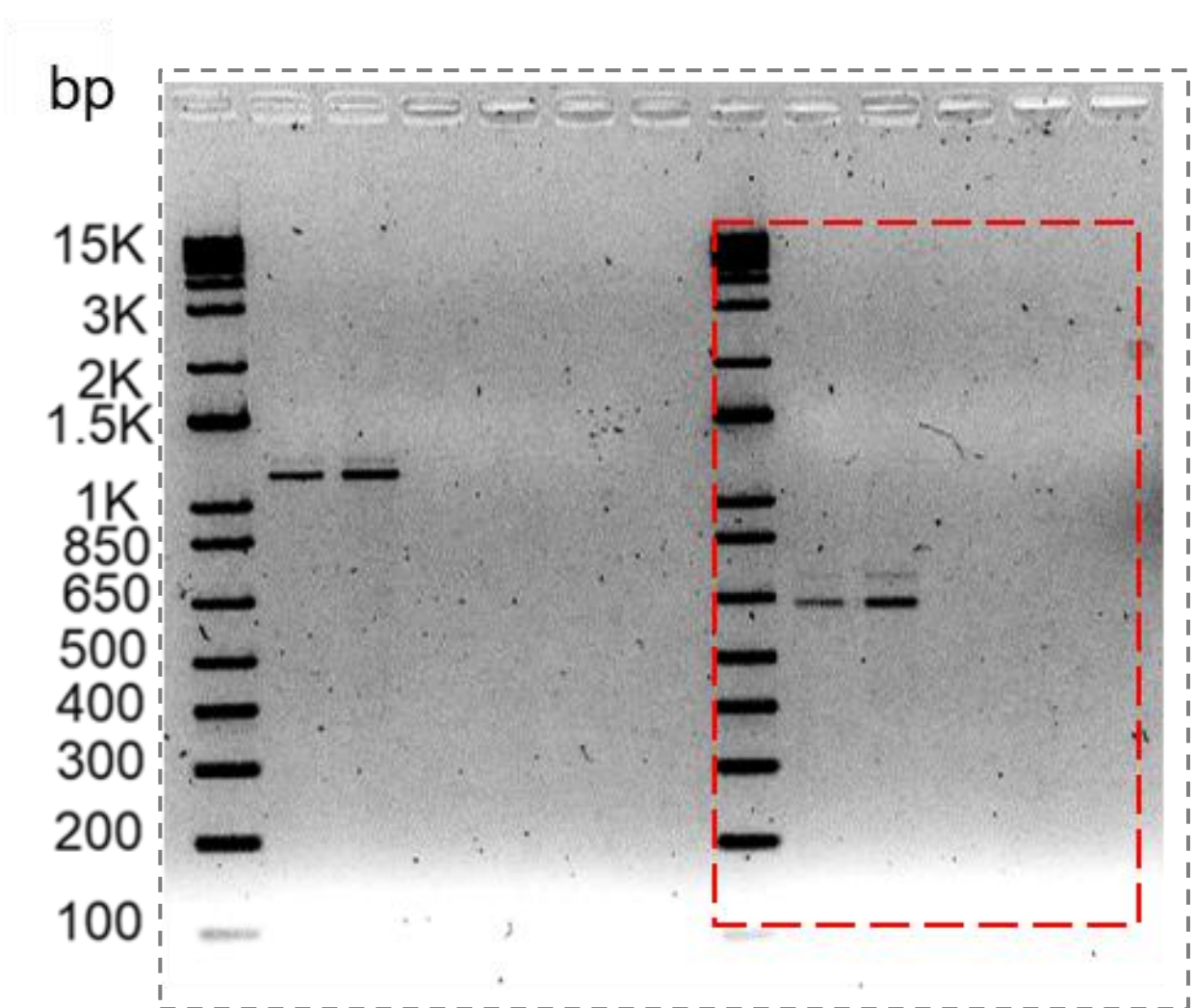

Red sections are included in the manuscript figure panels
Grey dotted borders: full uncropped blot scans

Figure S5A

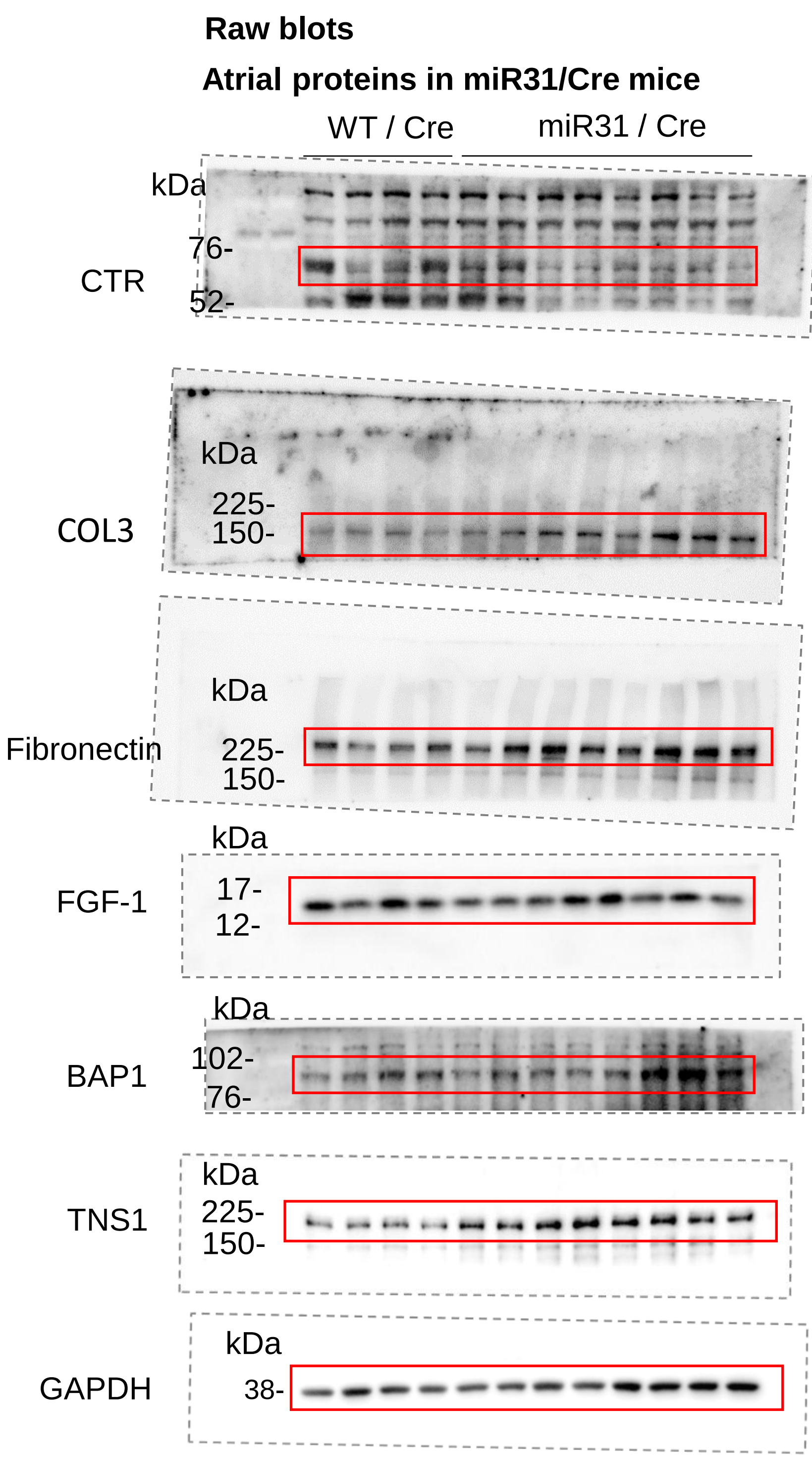

In red - included in the manuscript figure panels and quantification
Grey dotted borders: full uncropped blot scans

Extended Data Figure 5B

Raw blots
Atrial proteins in miR31/Cre mice

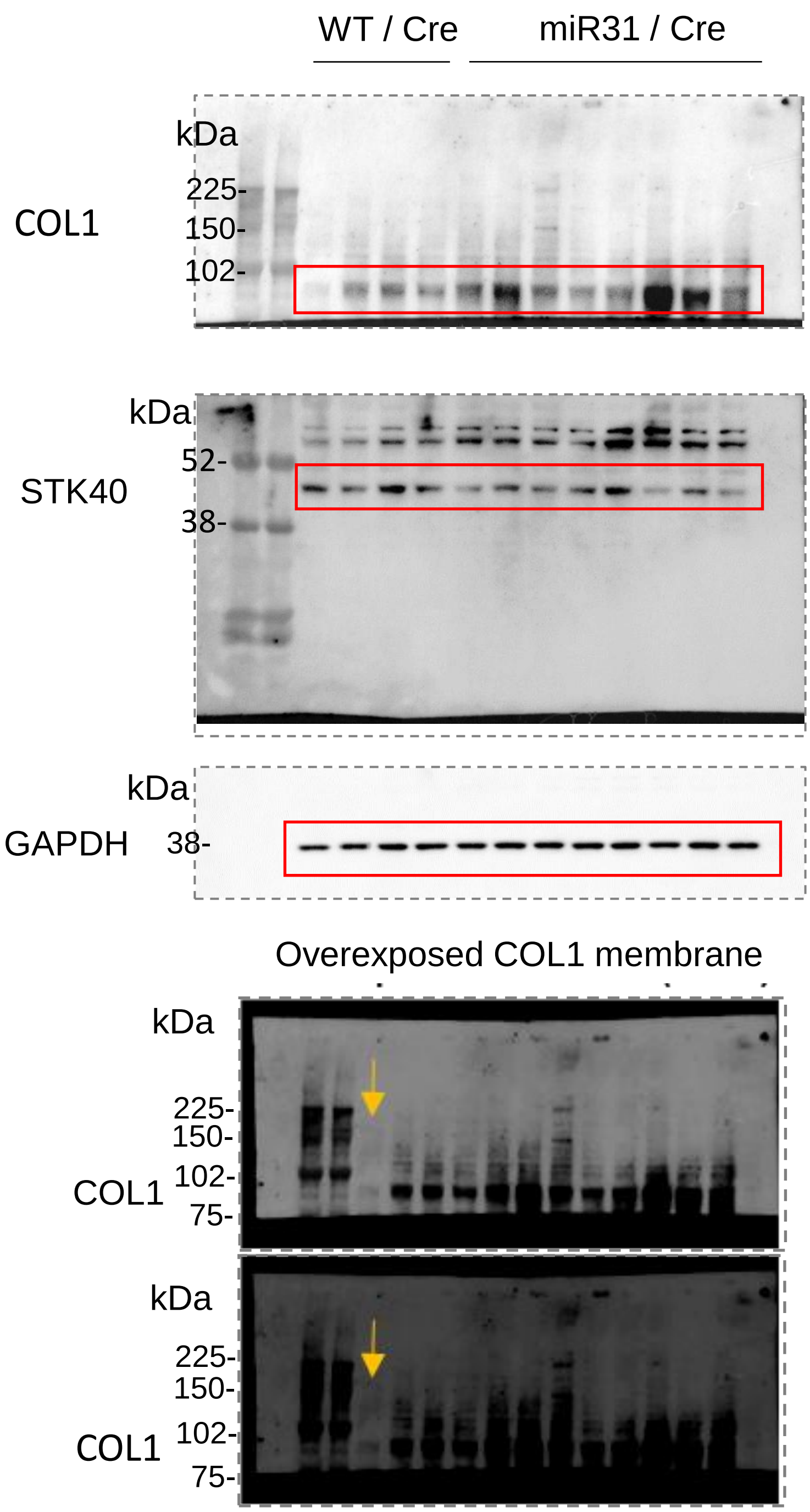

In red - included in the manuscript figure panels and quantification
Grey dotted borders: full uncropped blot scans

Extended Data Figure 5C

Raw blots
Atrial αSMA in miR31/Cre mice

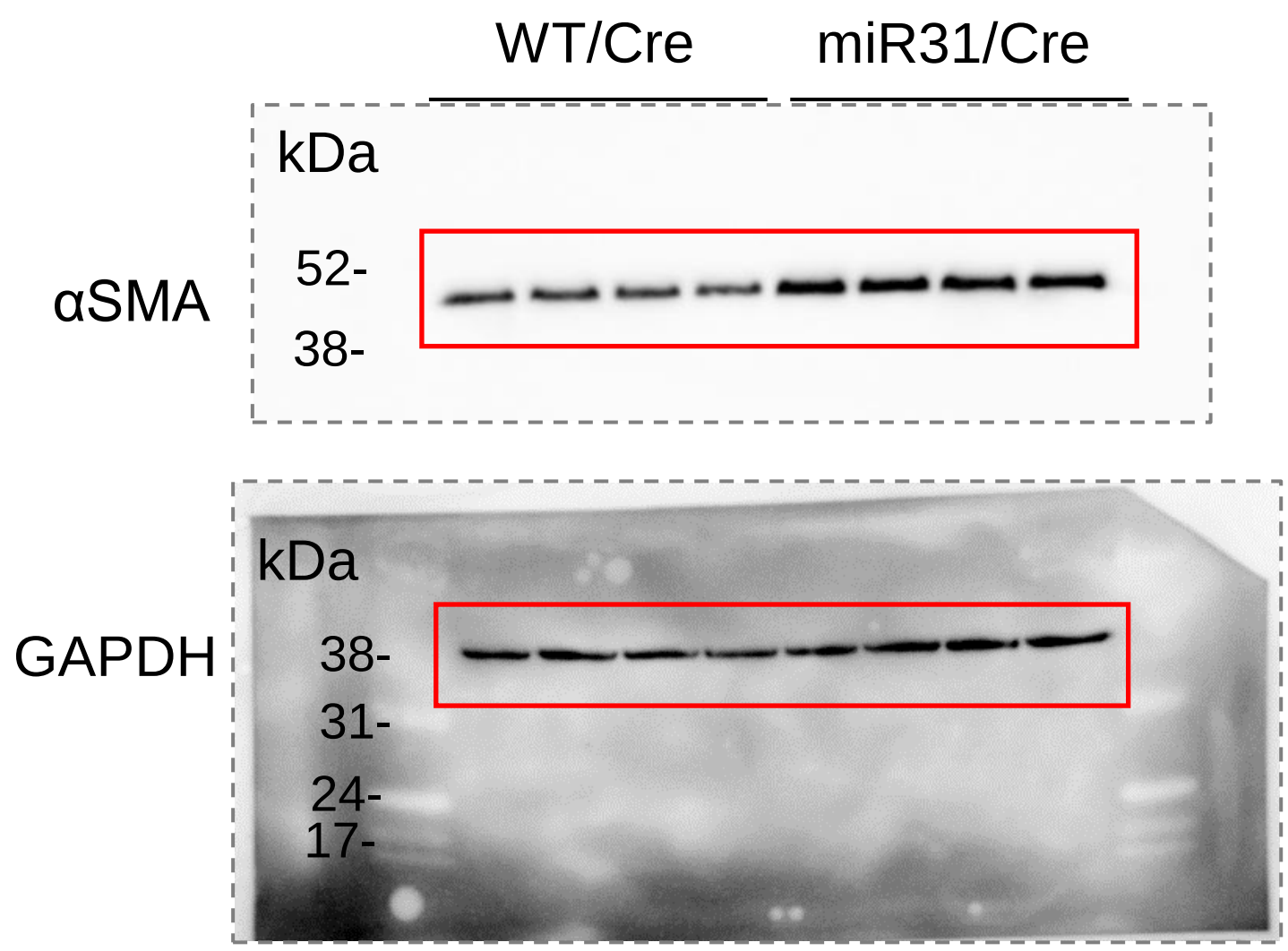

In red - included in the manuscript figure panels and quantification
Grey dotted borders: full uncropped blot scans

Extended Data Figure 5H

Raw blots

Left ventricular proteins in miR31/Cre mice

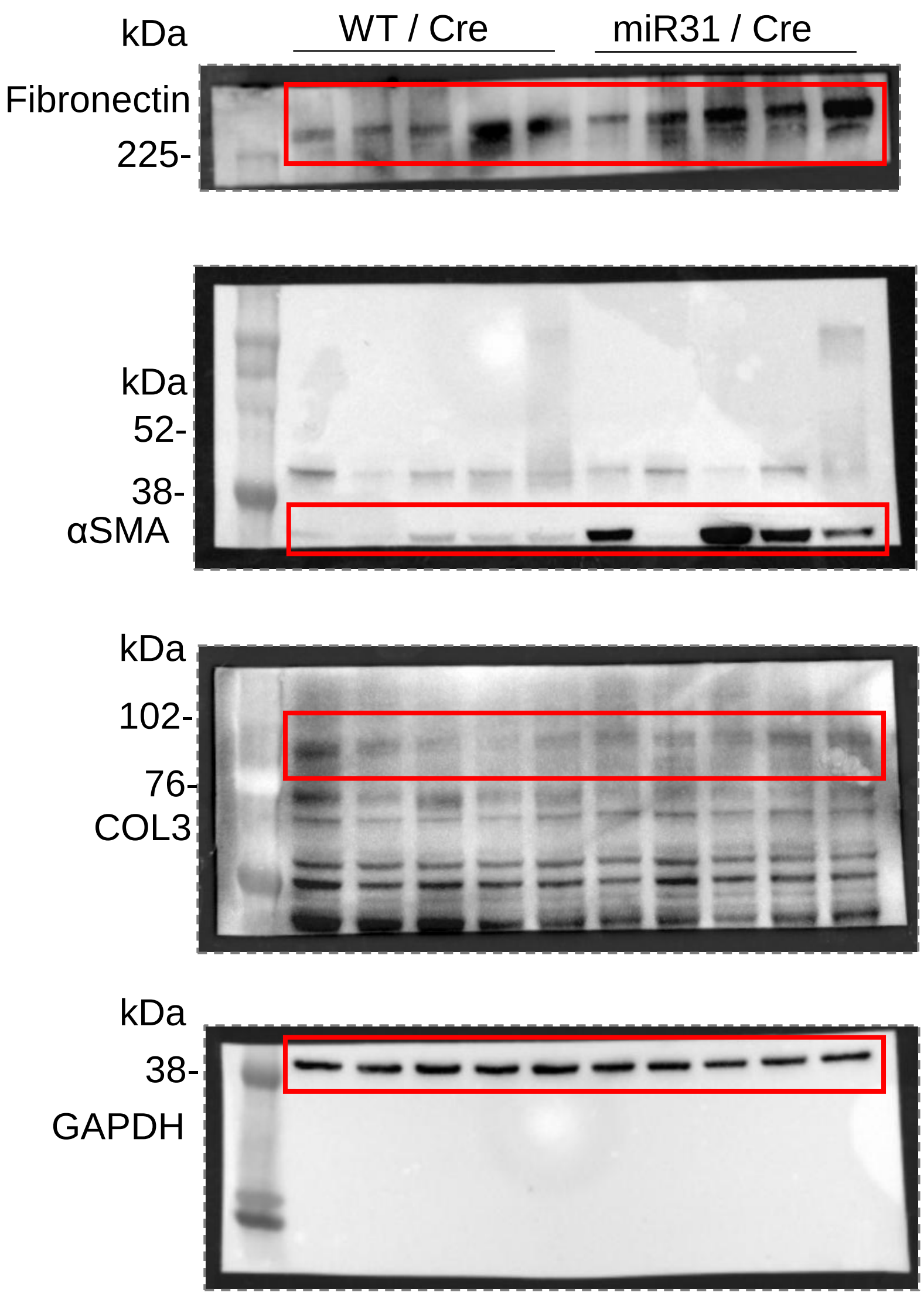

In red - included in the manuscript figure panels and quantification
Grey dotted borders: full uncropped blot scans

Extended Data Figure 5I

Raw blots

Left ventricular proteins in miR31/Cre mice

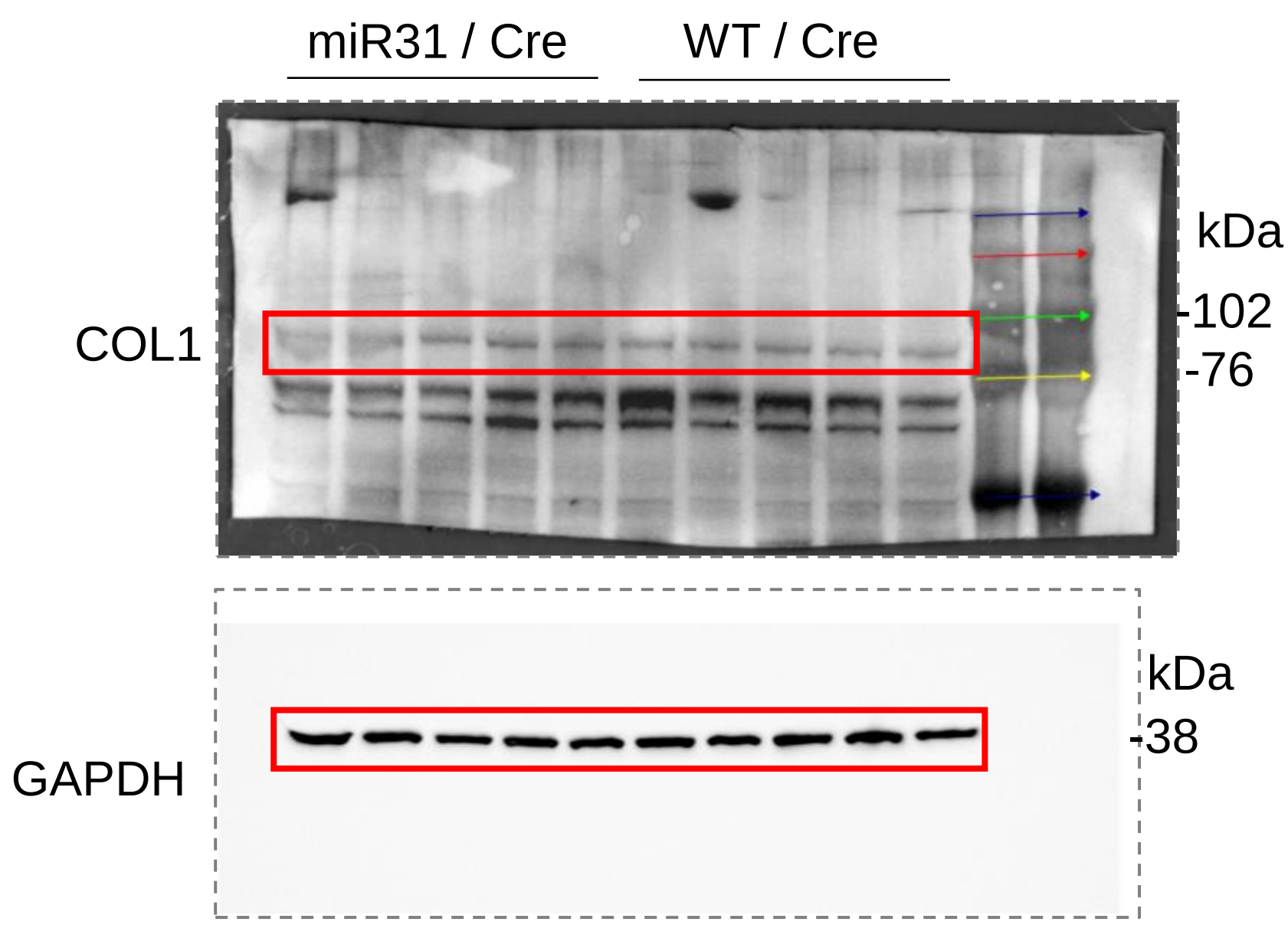

In red - included in the manuscript figure panels and quantification
Grey dotted borders: full uncropped blot scans
